# Comparative evaluation of large language models for esophageal cancer patient education: quality, reliability, readability, understandability, and actionability

**DOI:** 10.3389/fmed.2026.1973075

**Published:** 2026-10-14

**Authors:** Ying Ying Hang, Rui Yuan, Man Lu, Fang Wen Long, His Quan Hsu, Lu Bai, Jun Qian

**Affiliations:** 1 Department of Oncology, Affiliated Hospital of Nanjing University of Chinese Medicine, Nanjing, Jiangsu, China; 2 The First Clinical Medical College of Nanjing University of Chinese Medicine, Nanjing, China; 3 Jiangsu Collaborative Innovation Center of TCM Cancer Prevention and Treatment, Nanjing, China

**Keywords:** esophageal cancer, health information quality, large language models, patient education, readability

## Abstract

**Background:**

Large language models (LLMs) are increasingly used as sources of health information. This study evaluated the quality, reliability, readability, understandability, and actionability of responses generated by three mainstream LLMs to esophageal cancer-related questions.

**Methods:**

Thirty esophageal cancer-related questions covering six domains were identified from Google Trends, clinical practice guidelines, and social media and submitted to three LLMs, yielding 90 responses. Responses were evaluated using the Global Quality Scale (GQS), modified DISCERN (mDISCERN), Ensuring Quality Information for Patients (EQIP), Quality Assessment of Medical Artificial Intelligence (QAMAI), Patient Education Materials Assessment Tool (PEMAT), and six readability indices. Differences among the models were assessed using repeated-measures ANOVA or the Friedman test according to data distribution.

**Results:**

Crucial information missing was the most common content deficiency (8.9%). Overall GQS, mDISCERN, QAMAI, and EQIP scores were 4.00 (IQR: 4.00–4.00), 4.00 (IQR: 3.50–4.00), 22.00 (IQR: 21.50–25.00), and 71.88 (IQR: 62.50–75.00), respectively. DeepSeek performed best overall, followed by ChatGPT and Doubao. None of the three models met the recommended sixth-grade reading level, and all had PEMAT-A scores below the recommended 70% threshold. Readability was generally not significantly associated with quality or reliability, except for a few weak negative correlations.

**Conclusions:**

LLMs demonstrated generally favorable quality and reliability when answering esophageal cancer-related questions, but readability and actionability remained limited. They may serve as supplementary tools for patients seeking esophageal cancer-related health information but should not replace professional medical consultation.

## 1 Introduction

Esophageal cancer remains one of the most lethal malignancies worldwide, with persistently high incidence and mortality rates. According to the 2022 GLOBOCAN statistics, esophageal cancer ranked 11th in global cancer incidence and 7th among cancer-related causes of death. Approximately 511,000 new cases and 445,000 deaths were attributed to esophageal cancer globally (1). In China, the disease burden is particularly substantial, with approximately 224,000 new cases and 187,500 deaths, accounting for 43.8% of global incidence and 42.1% of global mortality, respectively (2). As a solid tumor with an exceedingly poor prognosis, esophageal cancer has an overall 5-year survival rate of less than 30% (3). Its clinical trajectory is protracted and complex, frequently involving multidisciplinary comprehensive treatment encompassing early screening, endoscopic or surgical intervention, chemoradiotherapy, and immunotherapy or targeted therapy, with patients confronting vastly different symptom burdens and decision-making challenges at each stage. However, patients' thirst for disease-related knowledge has long outpaced what limited consultation time can cover. Moreover, traditional patient education materials, whether paper-based or web-based, are often hampered by outdated content, inadequate readability, and lack of interactivity, rendering them insufficient to meet the pressing needs for timely, personalized health information across the disease trajectory (4).

In recent years, conversational artificial intelligence systems powered by large language models (LLMs), such as ChatGPT and DeepSeek, have rapidly emerged as novel channels for public health information seeking, offering distinct advantages including real-time responsiveness, 24/7 accessibility, and multi-turn interactive dialogue capabilities. Prior studies have preliminarily explored the application potential of LLMs in oncology. In lung cancer, LLMs were found to provide more concise and higher-quality patient education information compared to public websites (5). In prostate cancer, clinicians rated LLM responses as generally relevant and correct, yet readability across all models exceeded recommended levels (6). In colorectal cancer, gastric cancer, and head and neck cancer, LLM-generated patient education materials were comparable in quality to professional resources, though readability remained a key limitation (7–9). Additional studies have also been reported in breast cancer and cancer survivorship care (10, 11).

In esophageal cancer, prior studies have preliminarily evaluated the effectiveness of LLMs in patient education, yet they have primarily focused on earlier model versions and relied on limited assessment tools (12–14). However, none of these studies have employed the newly released DeepSeek-V4 Flash and ChatGPT-5.5 Instant models, nor have they assessed the understandability and actionability of the responses, namely whether patients can truly comprehend the information and act upon it. This knowledge gap implies that numerous patients with esophageal cancer and their caregivers may be exposed, without professional oversight, to recommendations of varying quality, potential inaccuracies, or critical omissions, thereby undermining the foundation of shared decision-making and potentially adversely affecting treatment choices.

This study evaluated the quality, reliability, readability, understandability, and actionability of responses generated by three mainstream large language model chatbots, namely Doubao, DeepSeek-V4 Flash, and ChatGPT-5.5 Instant, to common questions raised by patients with esophageal cancer. This study aimed to evaluate the performance and limitations of large language models in providing health information on esophageal cancer, and to provide preliminary evidence for their potential value as supplementary tools for patient education.

## 2 Methods

### 2.1 Question development

First, the research team used the search term “esophageal cancer” in Google Trends (https://trends.google.com) to identify global search trends over the preceding 5 years and extracted the most frequently searched related keywords and phrases. Second, we systematically reviewed authoritative clinical practice guidelines for esophageal cancer, including the ESMO Clinical Practice Guideline (2022) (15), the NCCN Guidelines for Esophageal and Esophagogastric Junction Cancers (Version 2.2023) (16), the Japan Esophageal Society Esophageal Cancer Practice Guidelines (2022; Part 1 and Part 2) (17, 18), the British Society of Gastroenterology Guidelines for the Management of Oesophageal and Gastric Cancer (2011) (19), and the ASCO Guideline for Treatment of Locally Advanced Esophageal Carcinoma (2020) (20). Two researchers with backgrounds in clinical oncology independently extracted relevant questions and reached consensus through discussion. Core guideline recommendations were subsequently reformulated into patient friendly questions. Finally, to ensure that the included questions reflected the real world concerns of patients and caregivers, Zhihu was selected as the social media data source. Zhihu has a large user base for health information sharing in China and can, to some extent, reflect the information needs of patients and caregivers in real world settings. Using “esophageal cancer” as the search term, the research team identified the 20 most highly discussed relevant posts and extracted common questions, major concerns, and potential information gaps.

All candidate questions were then screened according to predefined inclusion and exclusion criteria. The inclusion criteria were as follows: (1) directly related to esophageal cancer; (2) reflected common patient information needs or issues of clinical importance; and (3) were clearly phrased and could be understood and answered without additional contextual information. The exclusion criteria were as follows: (1) unrelated to esophageal cancer; (2) duplicated or highly similar to other candidate questions; (3) overly broad or ambiguous, making it difficult to generate a meaningful response; (4) contained advertising or promotional content; or (5) consisted primarily of a personal medical history or a detailed description of an individual clinical situation.

The final question set comprised 30 questions and was compiled as a standalone document (Table 1). All questions were presented in English, and all prompts were thus submitted to the models in English. Questions derived from Zhihu, which were originally in Chinese, were independently translated into English by two bilingual researchers, with any discrepancies resolved through discussion to ensure semantic equivalence. The questions covered six domains, including etiology, clinical manifestations, diagnosis, treatment, prognosis, and daily care. Each question was formulated as an independent and self contained prompt without any additional contextual information to simulate real world patient interactions with large language model chatbots. All prompts were entered verbatim as single turn queries to reflect typical public use of chatbot interfaces. No iterative prompt refinement or follow up prompting was performed during the study.

**Table 1:** List of common patient questions about esophageal cancer used as exact prompts across all LLM platforms.

Question No.	Question Category	Question
1	A. Etiology	What are the main risk factors for esophageal cancer?
2	A. Etiology	What are the differences in causes between esophageal squamous cell carcinoma and adenocarcinoma?
3	A. Etiology	What are the precancerous lesions of esophageal cancer?
4	A. Etiology	Is esophageal cancer hereditary?
5	A. Etiology	What is the peak age of onset for esophageal cancer?
6	B. Clinical Manifestations	What early symptoms of esophageal cancer should I be alert for?
7	B. Clinical Manifestations	What are the clinical manifestations of esophageal cancer?
8	B. Clinical Manifestations	How can I tell whether my esophageal cancer symptoms are worsening?
9	B. Clinical Manifestations	Which organs does esophageal cancer most commonly spread to, and what symptoms occur after metastasis?
10	B. Clinical Manifestations	What emergency situations in patients with esophageal cancer require immediate visit to the emergency department?
11	C. Diagnosis	Which department should I visit after being diagnosed with esophageal cancer?
12	C. Diagnosis	What is the gold standard for diagnosing esophageal cancer?
13	C. Diagnosis	After being diagnosed with esophageal cancer, what additional tests are needed to determine the severity of the disease?
14	C. Diagnosis	What stage is my esophageal cancer?
15	C. Diagnosis	What do the HER2, PD-L1, dMMR/MSI, and Claudin18.2 indicators mean in the esophageal cancer pathology report?
16	D. Treatment	Is endoscopic resection suitable for early-stage esophageal cancer?
17	D. Treatment	What are the surgical approaches for esophageal cancer?
18	D. Treatment	Why is chemoradiotherapy needed before surgery for esophageal cancer?
19	D. Treatment	What are the most commonly used chemotherapy, immunotherapy, and targeted therapy drugs for advanced esophageal cancer?
20	D. Treatment	What treatment options are available for esophageal cancer from early to advanced stages?
21	D. Treatment	On what basis does the doctor formulate my treatment plan for esophageal cancer?
22	E. Prognosis	What is the probability of cure for early-stage esophageal cancer?
23	E. Prognosis	What is the approximate survival rate for my stage of esophageal cancer?
24	E. Prognosis	Can esophageal cancer recur after treatment is completed?
25	E. Prognosis	What major factors affect the long-term prognosis of esophageal cancer patients?
26	F. Daily Care	What are the common side effects during chemotherapy, radiotherapy, targeted therapy, or immunotherapy for esophageal cancer, and how should I manage them?
27	F. Daily Care	How should I maintain my nutrition during treatment for esophageal cancer?
28	F. Daily Care	How should I gradually resume eating after esophageal cancer surgery?
29	F. Daily Care	How should the pain caused by esophageal cancer be managed?
30	F. Daily Care	How often should I have follow-up examinations after treatment for esophageal cancer, and what items are included?

### 2.2 Model selection and response collection

Three publicly accessible LLM chatbots were selected for evaluation based on their widespread use in China and relevance to patient education: Doubao (ByteDance), DeepSeek-V4 Flash (DeepSeek AI), and ChatGPT-5.5 Instant (OpenAI).

Doubao, developed by ByteDance, was officially released on May 15, 2024, at the Volcano Engine FORCE Conference. By the end of 2025, Doubao had achieved 226 million monthly active users, making it one of the largest native AI applications in China. DeepSeek-V4 Flash was released as a preview version by DeepSeek AI on April 24, 2026, and was simultaneously open-sourced globally. It has 284 billion total parameters with 13 billion activated parameters and natively supports a 1-million-token context window, achieving substantial improvements in long-text processing efficiency. ChatGPT-5.5 Instant was released by OpenAI on May 5, 2026, replacing GPT-5.3 Instant as the default conversational model for ChatGPT. This version features optimizations for medical Q&A scenarios, including improved recognition of urgent care needs, contextual inquiry, and uncertainty communication. All models were accessed via their official web interfaces in their default configurations without task-specific fine-tuning, parameter optimization, retrieval-augmented generation, or external post-processing. For proprietary models, detailed disclosures regarding training corpora and internal architectures were not publicly available; therefore, performance evaluation was based on capabilities as described in official vendor documentation.

Responses were collected on July 22, 2026, in a controlled environment to ensure consistency. Each query was entered into a new chat session after clearing browser cache to minimize possible context carryover effects. Each question was submitted independently to each of the three models, yielding 90 responses for analysis (30 questions  ×  3 models). All responses were generated using the models' default settings to reflect real-world patient use conditions.

All chatbot responses were captured verbatim and stored without modification. Prior to expert evaluation, all responses were de-identified and assigned numerical codes to mask model identity. Evaluators were blinded to the chatbot system used during both quantitative scoring and qualitative error analysis. The complete chatbot response set is provided in Supplementary Table 1. No personal or identifiable information was involved in the dataset.

### 2.3 Evaluation instruments

This study used multiple evaluation instruments to assess the quality, reliability, readability, understandability, and actionability of responses generated by large language models.

#### 2.3.1 Quality and reliability

Four instruments were used to evaluate the quality and reliability of the responses. Their full item wording is provided in Supplementary Tables 2 to 5.

The Global Quality Scale (GQS) is a 5-point Likert-type scale used to assess the overall quality of health information, including its completeness, content quality, and organization (21). Scores range from 1 (poor quality) to 5 (excellent quality), with higher scores indicating better overall information quality.

The modified DISCERN (mDISCERN) instrument comprises five items evaluating the reliability of consumer health information, assessing whether: (1) the aim is clear, concise, and understandable; (2) the sources of information are reliable; (3) the information is presented in a balanced and unbiased manner; (4) additional sources of information are listed for patient reference; and (5) areas of uncertainty are mentioned (22). Each item is scored as 0 or 1 (0 = no, 1 = yes), yielding a total score ranging from 0 to 5, with higher scores indicating greater reliability.

The Ensuring Quality Information for Patients (EQIP) scale is a 20-item instrument measuring the quality of patient information across content, structure, and presentation domains (23). Full compliance is scored as 1 point (“yes”), partial compliance as 0.5 points (“partly”), and non-compliance as 0 points (“no”). The final score is expressed as a percentage, calculated as [(Yes  ×  1 + Partly  ×  0.5 + No × 0)/20] × 100. Quality classification is as follows: 0%–25%, severe quality problems; 26%–50%, serious quality issues; 51%–75%, good quality with minor issues; 76%–100%, well written.

The Quality Assessment of Medical Artificial Intelligence (QAMAI) tool is a 6-item instrument specifically designed to evaluate the quality of AI-generated medical information across six domains: accuracy, clarity, relevance, completeness, provision of sources and references, and usefulness (24). Each item is scored on a 0–5 scale, yielding a total score ranging from 0 to 30, with higher scores indicating better quality.

#### 2.3.2 Readability

A free online readability application (https://readabilityformulas.com) was used to assess the reading ease of each response, utilizing six validated indices: the Automated Readability Index (ARI), the Flesch Reading Ease Score (FRES), the Gunning Fog Index (GFI), the Flesch Kincaid Grade Level (FKGL), the Coleman Liau Index (CLI), and the Simple Measure of Gobbledygook (SMOG) index (25). The ARI estimates the U.S. grade level required to comprehend the text based on characters per word and words per sentence. The FRES produces a score from 0 to 100, with higher scores indicating easier readability; scores below 30 are considered “very difficult” to read. The FKGL converts the text to a U.S. grade-level equivalent. The FRES and FKGL readability categories and grade level estimates are provided in Supplementary Table 6. The GFI estimates the years of formal education required to understand the text on first reading. The CLI estimates grade level based on characters per word and sentences per 100 words. The SMOG index estimates the years of education needed to fully comprehend the text. The formulas used to calculate each index are provided in Supplementary Table 7.

#### 2.3.3 Understandability and actionability

The Patient Education Materials Assessment Tool (PEMAT) was used to assess the understandability and actionability of the responses. The PEMAT was administered according to the scoring instructions provided on the Agency for Healthcare Research and Quality website (https://www.ahrq.gov/health-literacy/patient-education/pemat-p.html) (26). The PEMAT comprises two subscales: PEMAT-Understandability (PEMAT-U), which evaluates how easily patients can process and explain the key messages, and PEMAT-Actionability (PEMAT-A), which assesses the extent to which the materials identify actionable steps that patients can take. Both subscales are scored as percentages, with higher scores indicating better performance. A score of ≥70% is generally considered acceptable for both domains.

### 2.4 Evaluation process

Two independent evaluators, one attending physician and one senior nurse, each with over 10 years of clinical experience in esophageal cancer, independently assessed all 90 de-identified responses using a double-blinded approach, with evaluators blinded to the model identity of the response sources. Prior to formal evaluation, both evaluators received standardized training on the GQS, mDISCERN, EQIP, QAMAI, and PEMAT scoring criteria, and completed calibration using 10 pilot responses (26). The clinical practice guidelines on esophageal cancer reviewed during question development also served as the clinical reference standard for all assessments (15–20). When substantial disagreement occurred, a third senior clinician with more than 20 years of clinical experience was consulted to resolve the discrepancy and ensure data quality; however, the final scores used for analysis were the mean of the two independent raters' scores (27). Intraclass correlation coefficients (ICCs) were calculated using the original independent ratings to assess inter-rater reliability. The overall study workflow is illustrated in Figure 1.

**Figure 1:**
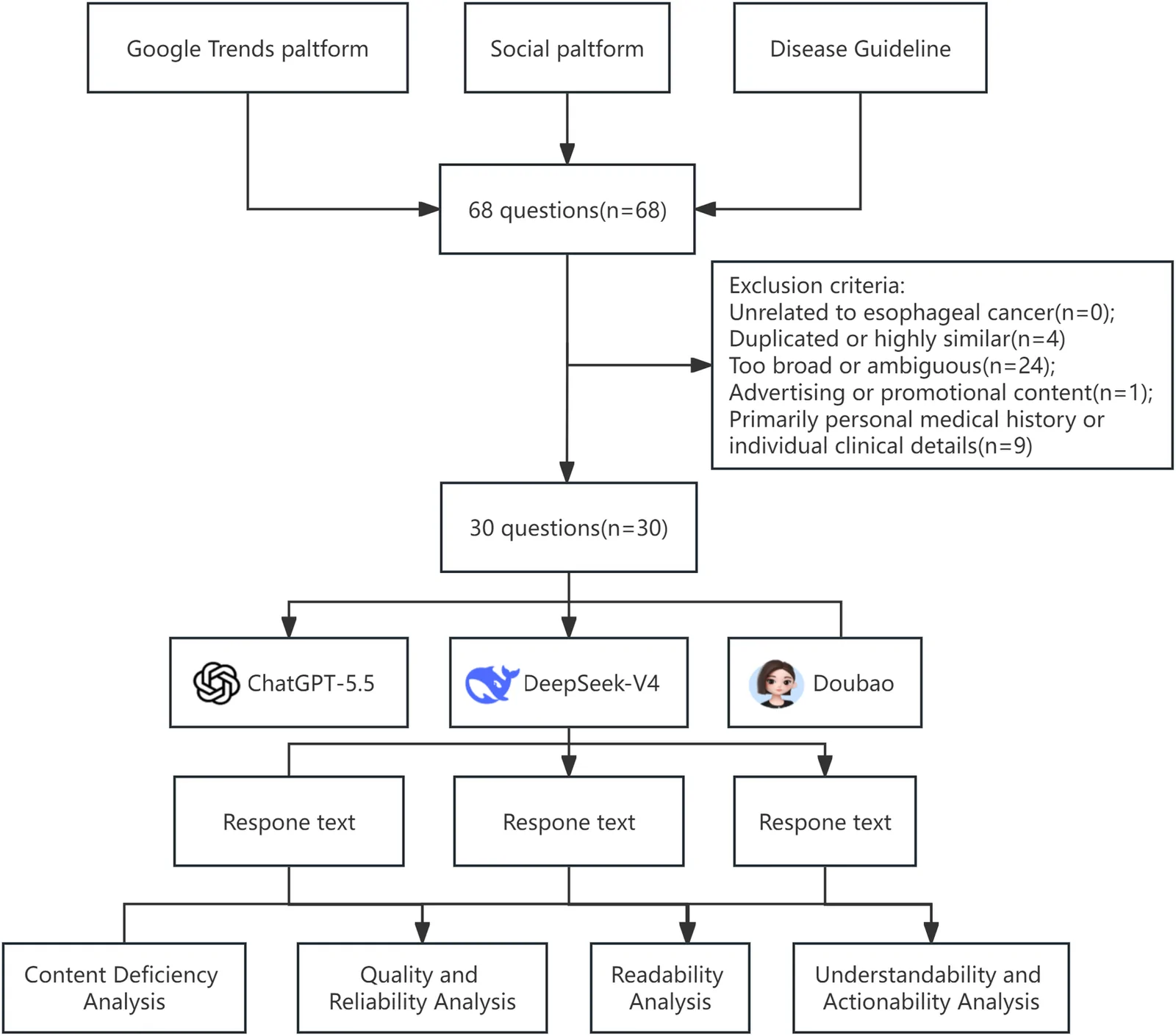
Study workflow illustrating the question development, model selection, response collection, and evaluation process.

### 2.5 Statistical analysis

All statistical analyses were performed using R software (version 4.3.2). Descriptive statistics were calculated for all evaluation instruments and readability indices. Model comparisons were conducted within a repeated-measures design, in which each question was answered by all three models. Normally distributed variables were presented as the mean ± standard deviation (SD) and compared using one-way repeated-measures analysis of variance (RM-ANOVA). When the overall test reached statistical significance, *post hoc* pairwise comparisons were performed, with *P*-values adjusted using the Bonferroni correction. Non-normally distributed variables were presented as the median and interquartile range (IQR). When the overall test reached statistical significance, *post hoc* pairwise comparisons were performed, with *P*-values adjusted using the Bonferroni correction. Inter-rater reliability was assessed using the ICC with a two-way random-effects, absolute agreement, single-measurement model. A reference value of 6 was used for the ARI, CLI, FKGL, GFI, and SMOG, whereas a reference value of 80 was used for the FRES. Associations among evaluation metrics were examined using Spearman's rank correlation coefficient. All tests were two-sided, and *P* < 0.05 was considered statistically significant.

## 3 Results

### 3.1 Inter-rater reliability

Inter-rater reliability between the two independent evaluators across all six measurement instruments is presented in Table 2. The ICC for GQS was 0.858 (95% CI: 0.755–0.960). The ICC for mDISCERN was 0.847 (95% CI: 0.718–0.977). The ICC for EQIP was 0.833 (95% CI: 0.717–0.898). The ICC for QAMAI was 0.849 (95% CI: 0.778–0.898). The ICC for PEMAT-U was 0.836 (95% CI: 0.609–0.918). The ICC for PEMAT-A was 0.851 (95% CI: 0.768–0.903). All ICC values exceeded 0.80, with *P* < 0.01 for all instruments, indicating excellent and statistically significant inter-rater reliability across all assessment tools.

**Table 2:** Intraclass correlation coefficient between two raters for different parameters.

Metric	ICC	CI lower	CI upper	*p*
GQS	0.858	0.755	0.960	<0.01
mDISCERN	0.847	0.718	0.977	<0.01
EQIP	0.833	0.717	0.898	<0.01
QAMAI	0.849	0.778	0.898	<0.01
PEMAT-U	0.836	0.609	0.918	<0.01
PEMAT-A	0.851	0.768	0.903	<0.01

### 3.2 Qualitative deficiencies

Qualitative expert review identified four categories of content related deficiencies in the 90 chatbot generated responses: crucial information missing (responses lacking guideline recommended information essential for patient understanding or safety), factual inaccuracies (responses containing incorrect statements or misrepresented facts), hallucinations (fabricated or unsupported content not grounded in evidence or guidelines), and provided outdated medical advice (information that is obsolete or no longer recommended). A single response could be assigned more than one category when multiple distinct deficiencies were identified. The distribution of these deficiencies is presented in Table 3 and Figure 2.

**Table 3:** Distribution and proportion of qualitative deficiencies in response content.

Content	N(%)
Factual inaccuracies	6 (6.7)
Hallucination	5 (5.6)
Crucial information missing	8 (8.9)
Provided outdated medical advice	2 (2.2)

**Figure 2:**
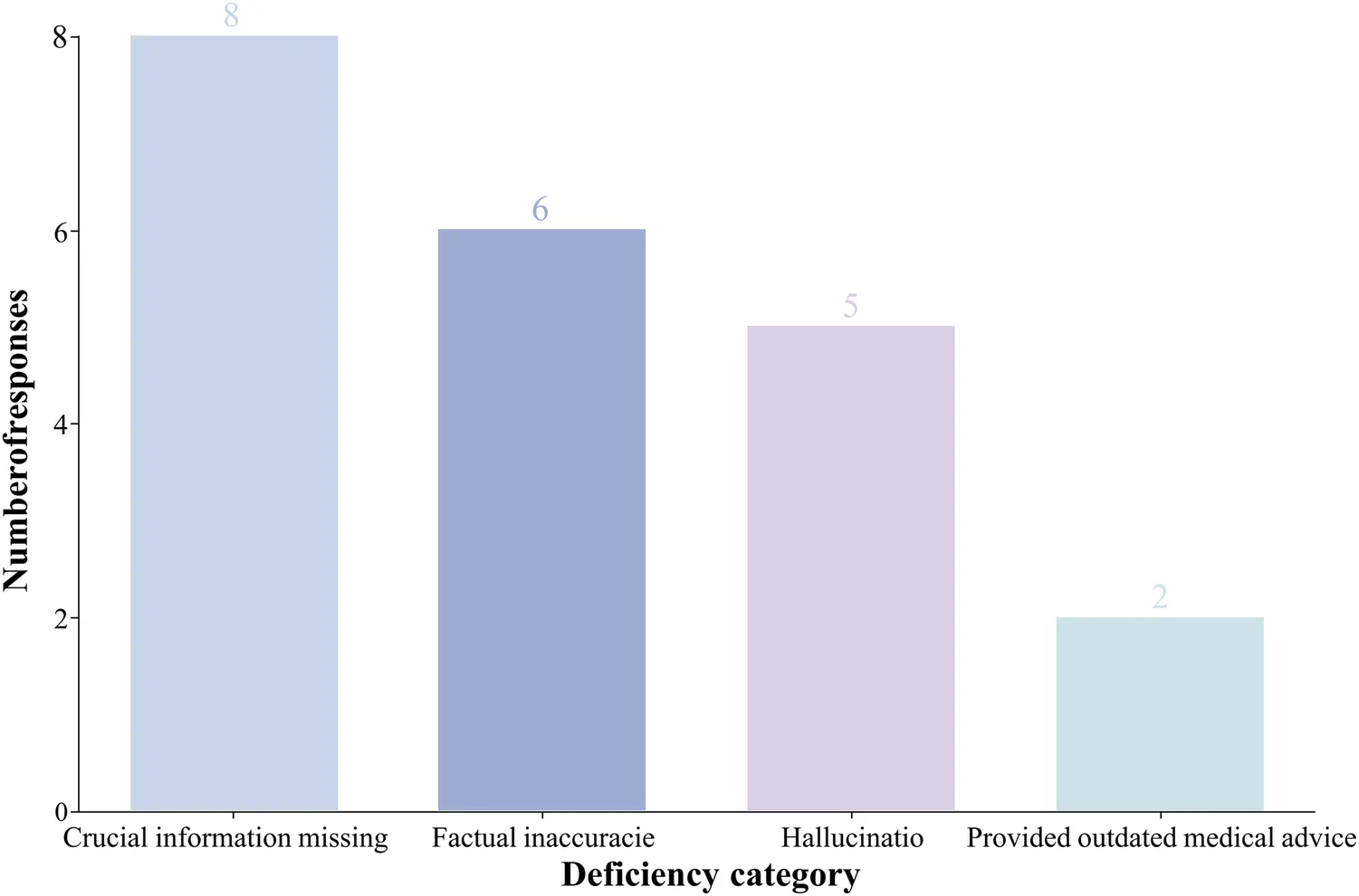
Distribution of qualitative deficiencies across all responses.

Crucial information missing was the most prevalent, occurring in 8.9% of all responses. Factual inaccuracies occurred in 6.7% of responses, hallucinations in 5.6%, and outdated medical advice was provided in 2.2%. The distribution of deficiencies varied across question categories (Table 4). Crucial information missing was most prevalent in diagnosis questions (37.5%). Factual inaccuracies were most prevalent in daily care questions (50.0%). Hallucinations were most frequently observed in prognosis questions (40.0%). Provided outdated medical advice was identified in both diagnosis and treatment questions (each at 50.0%).

**Table 4:** Distribution and proportion of qualitative deficiencies by question domain.

Question Category	Provided outdated medical advice N(%)	Hallucinations N(%)	Crucial information missing N(%)	Factual Inaccuracies N(%)
Etiology	0 (0.0)	1 (20.0)	0 (0.0)	0 (0.0)
Clinical Manifestations	0 (0.0)	0 (0.0)	1 (12.5)	0 (0.0)
Diagnosis	1 (50.0)	0 (0.0)	3 (37.5)	1 (16.7)
Treatment	1 (50.0)	1 (20.0)	0 (0.0)	1 (16.7)
Prognosis	0 (0.0)	2 (40.0)	2 (25.0)	1 (16.7)
Daily Care	0 (0.0)	1 (20.0)	2 (25.0)	3 (50.0)

### 3.3 Overall performance across assessment domains

The overall characteristics of the LLM-generated responses across all assessment domains are summarized in Table 5. For quality and reliability metrics, GQS and mDISCERN both achieved median scores of 4.00 (IQR: 4.00–4.00 and 3.50–4.00, respectively). The median QAMAI score was 22.00 (IQR: 21.50–25.00), and the median EQIP score was 71.88 (IQR: 62.50–75.00). The PEMAT-U scores had a median of 80.00 (IQR: 73.00–84.00). In contrast, the PEMAT-A scores had a median of 60.00 (IQR: 58.50–67.00), substantially below the recommended threshold of 70%. Readability assessment showed that the mean ARI was 15.16 ± 3.02, the FRES was 21.22 ± 17.14, the GFI was 15.72 ± 2.97, the FKGL was 14.31 ± 3.24, the CLI was 17.00 ± 3.08, and the SMOG index was 11.84 ± 2.42.

**Table 5:** Overall characteristics of the quality, reliability, readability, understandability, and actionability of large language model responses.

Variables	Total
GQS, M (Q₁, Q₃)	4.00 (4.00, 4.00)
mDISCERN, M (Q₁, Q₃)	4.00 (3.50, 4.00)
QAMAI, M (Q₁, Q₃)	22.00 (21.50, 25.00)
EQIP, M (Q₁, Q₃)	71.88 (62.50, 75.00)
PEMAT-U, M (Q₁, Q₃)	80.00 (73.00, 84.00)
PEMAT-A, M (Q₁, Q₃)	60.00 (58.50, 67.00)
ARI, Mean ± SD	15.16 ± 3.02
FRES, Mean ± SD	21.22 ± 17.14
GFI, Mean ± SD	15.72 ± 2.97
FKGL, Mean ± SD	14.31 ± 3.24
CLI, Mean ± SD	17.00 ± 3.08
SMOG, Mean ± SD	11.84 ± 2.42

### 3.4 Comparison of quality and reliability across platforms

The distributions of GQS, mDISCERN, QAMAI, and EQIP scores across the three models are shown in Figures 3A–D, respectively. For responses generated by DeepSeek, the median GQS score was 4.00 (IQR: 4.00–4.88), the median mDISCERN score was 4.00 (IQR: 4.00–4.00), the median QAMAI score was 25.50 (IQR: 25.12–26.50), and the median EQIP score was 75.00 (IQR: 72.50–77.50). Detailed results for the other two models are presented in Table 6. Compared with DeepSeek, Doubao had significantly lower scores for GQS (*P* < 0.05, Figure 4A), mDISCERN (*P* < 0.05, Figure 4B), QAMAI (*P* < 0.05, Figure 4C), and EQIP (*P* < 0.05, Figure 4D). In addition, DeepSeek achieved the highest scores among the three models for both QAMAI (*P* < 0.05, Figure 4C) and EQIP (*P* < 0.05, Figure 4D).

**Figure 3:**
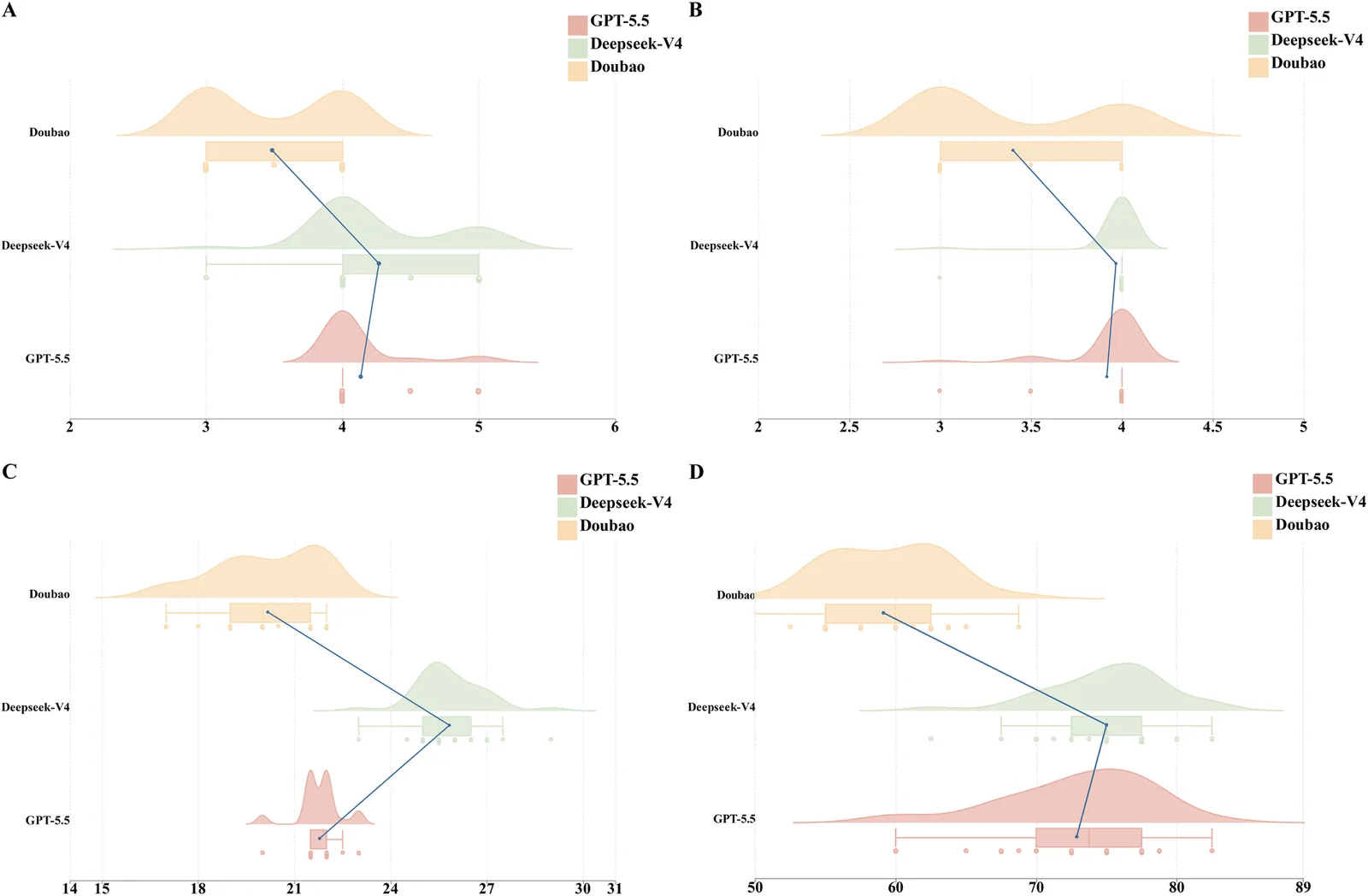
Distribution of GQS, mDISCERN, QAMAI, and EQIP scores across the three LLM platforms. **(A)** GQS scores. **(B)** mDISCERN scores. **(C)** QAMAI scores. **(D)** EQIP scores.

**Table 6:** Comparison of response quality and reliability across different large language models.

Variables	Doubao	DeepSeek	ChatGPT	*p*
GQS, M (Q₁, Q₃)	3.50 (3.00,4.00)	4.00 (4.00,4.88)	4.00 (4.00,4.00)	< 0.001
mDISCERN, M (Q₁, Q₃)	3.00 (3.00,4.00)	4.00 (4.00,4.00)	4.00 (4.00,4.00)	< 0.001
QAMAI, M (Q₁, Q₃)	20.00 (19.00,21.50)	25.50 (25.12,26.50)	22.00 (21.50,22.00)	< 0.001
EQIP, M (Q₁, Q₃)	60.00 (55.00,62.50)	75.00 (72.50,77.50)	73.75 (70.00,77.50)	< 0.001

**Figure 4:**
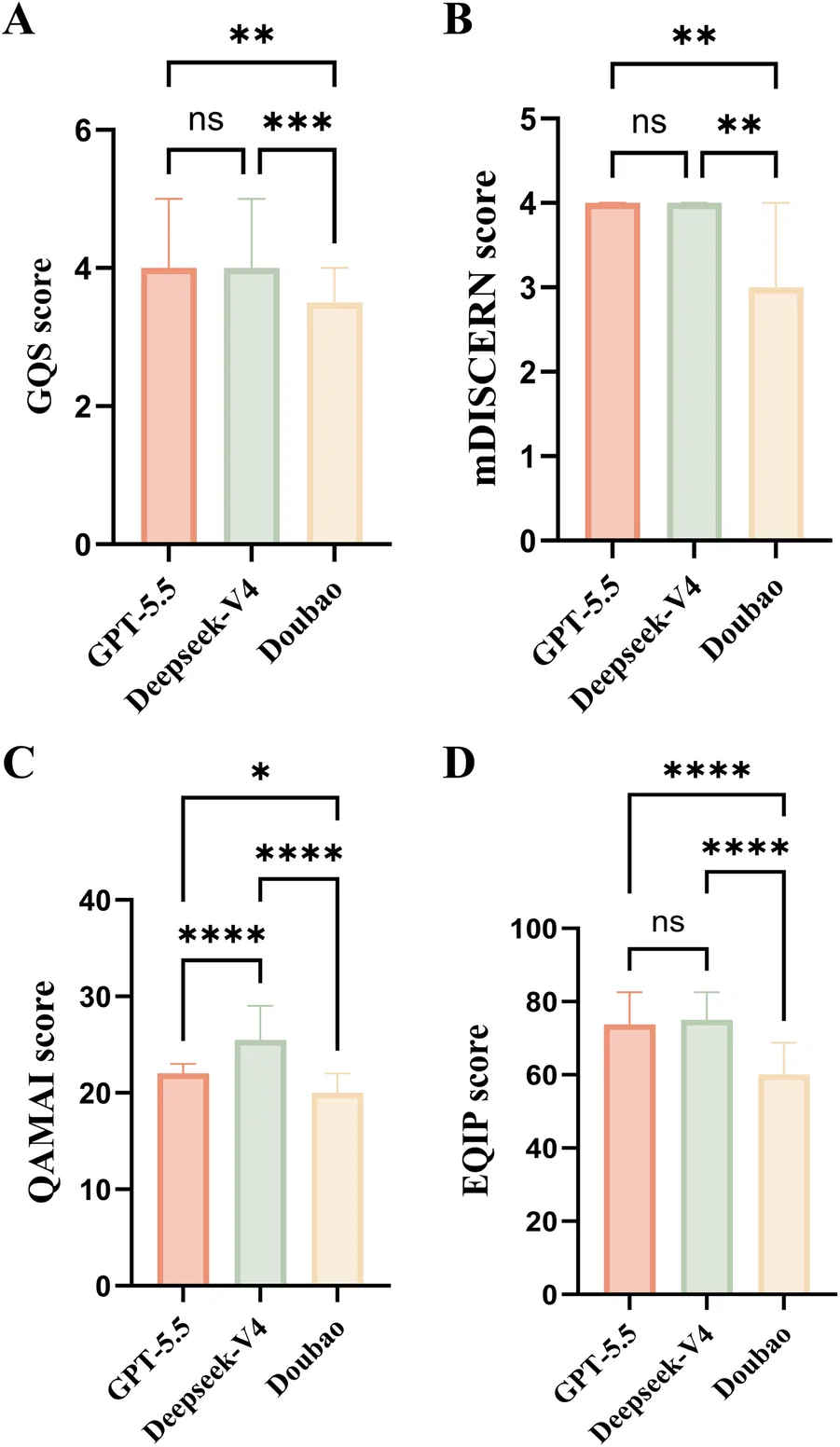
Comparison of GQS, mDISCERN, QAMAI, and EQIP scores across the three LLM platforms. **(A)** GQS scores. **(B)** mDISCERN scores. **(C)** QAMAI scores. **(D)** EQIP scores.

### 3.5 Comparison of readability across platforms

Table 7 and Figure 5A presents a radar plot comparing the six readability indices across the three models. DeepSeek showed the greatest variability across the readability measures, whereas Doubao exhibited the most consistent readability profile. Figure 5B further stratified readability by question domain. Across all models, responses to daily care questions were the easiest to read, whereas responses to treatment questions required higher reading levels. Compared with ChatGPT, Doubao had significantly higher ARI scores (16.39 ± 3.01 vs. 14.27 ± 2.85, *P* < 0.05, Figure 5C), GFI scores (17.13 ± 3.18 vs. 14.97 ± 2.51, *P* < 0.05, Figure 5D), FKGL scores (15.68 ± 3.58 vs. 13.55 ± 2.84, *P* < 0.05, Figure 5E), and SMOG scores (12.64 ± 2.44 vs. 11.01 ± 2.41, *P* < 0.05, Figure 5F), indicating that Doubao's responses were significantly more difficult to read. Compared with DeepSeek, Doubao showed poorer performance for GFI (17.13 ± 3.18 vs. 15.08 ± 2.77, *P* < 0.05, Figure 5D), FKGL (15.68 ± 3.58 vs. 13.68 ± 2.89, *P* < 0.05, Figure 5E), FRES (16.13 ± 17.76 vs. 26.00 ± 18.20, *P* < 0.05, Figure 5G), and CLI (18.17 ± 3.67 vs. 15.93 ± 2.82, *P* < 0.05, Figure 5H). Overall, although readability differed across the three models, none reached the recommended sixth-grade reading level in the United States.

**Table 7:** Comparison of response readability across different large language models.

Variables	Doubao	DeepSeek	ChatGPT	*p*
ARI, Mean ± SD	16.39 ± 3.01	14.82 ± 2.89	14.27 ± 2.85	0.0058
FRES, Mean ± SD	16.13 ± 17.76	26.00 ± 18.20	21.53 ± 14.29	0.0012
GFI, Mean ± SD	17.13 ± 3.18	15.08 ± 2.77	14.97 ± 2.51	< 0.001
FKGL, Mean ± SD	15.68 ± 3.58	13.68 ± 2.89	13.55 ± 2.84	< 0.001
CLI, Mean ± SD	18.17 ± 3.67	15.93 ± 2.82	16.89 ± 2.24	< 0.001
SMOG, Mean ± SD	12.64 ± 2.44	11.86 ± 2.18	11.01 ± 2.41	0.0105

**Figure 5:**
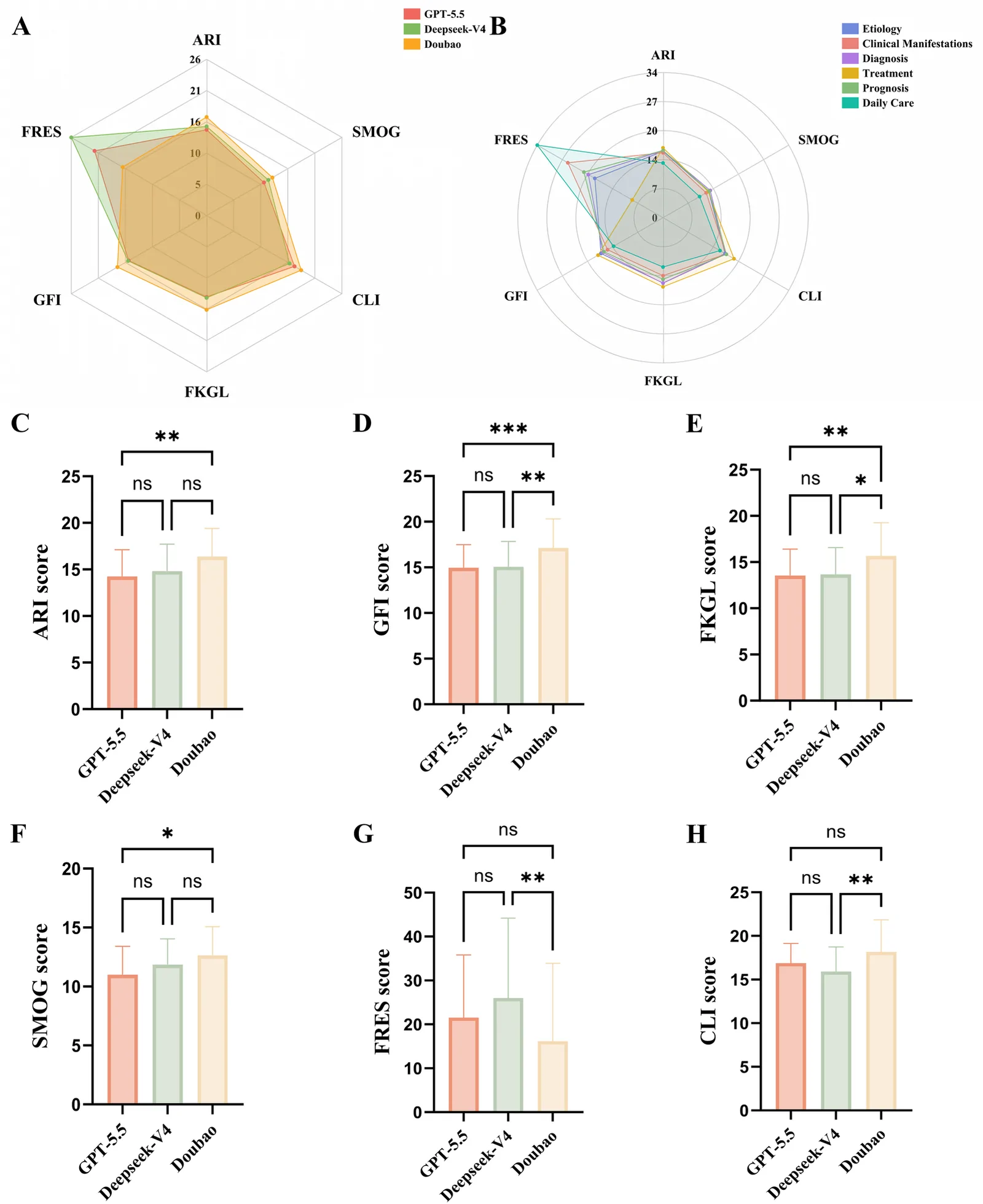
Comparison of readability scores across the three LLM platforms. **(A)** Radar plots of six readability indices across the three models (Doubao, DeepSeek, and ChatGPT). For the FRES axis, higher scores indicate better readability; for the ARI, GFI, FKGL, CLI, and SMOG axes, higher scores indicate lower readability (i.e., greater Reading difficulty). **(B)** Radar plots of readability indices stratified by question domain (etiology, clinical manifestations, diagnosis, treatment, prognosis, and daily care). **(C)** Automated Readability Index (ARI) scores. **(D)** Gunning Fog Index (GFI). **(E)** Flesch-Kincaid Grade Level (FKGL). **(F)** Simple Measure of Gobbledygook (SMOG) index. **(G)** Flesch Reading Ease Score (FRES). **(H)** Coleman-Liau Index (CLI).

### 3.6 Comparison of understandability and actionability across platforms

Table 8 summarizes the comparison of understandability (PEMAT-U) and actionability (PEMAT-A) across the three LLM platforms. Statistically significant differences were observed for both domains (*P* < 0.001). The distributions of these scores are shown in Figures 6A,6C, respectively. For PEMAT-U, DeepSeek scored 85.50 (IQR: 80.00–87.00), followed by ChatGPT at 80.00 (IQR: 76.50–83.88) and Doubao at 67.00 (IQR: 63.62–73.00) (Figure 6B). For PEMAT-A, DeepSeek scored 67.00 (IQR: 67.00–69.25), followed by ChatGPT at 60.00 (IQR: 60.00–65.25) and Doubao at 50.00 (IQR: 50.00–60.00) (Figure 6D). All three models fell below the recommended threshold of 70% for actionability.

**Table 8:** Comparison of response understandability and actionability across different large language models.

Variables	Doubao	DeepSeek	ChatGPT	*p*
PEMAT-U, M (Q₁, Q₃)	67.00 (63.62,73.00)	85.50 (80.00,87.00)	80.00 (76.50,83.88)	< 0.001
PEMAT-A, M (Q₁, Q₃)	50.00 (50.00,60.00)	67.00 (67.00,69.25)	60.00 (60.00,65.25)	< 0.001

**Figure 6:**
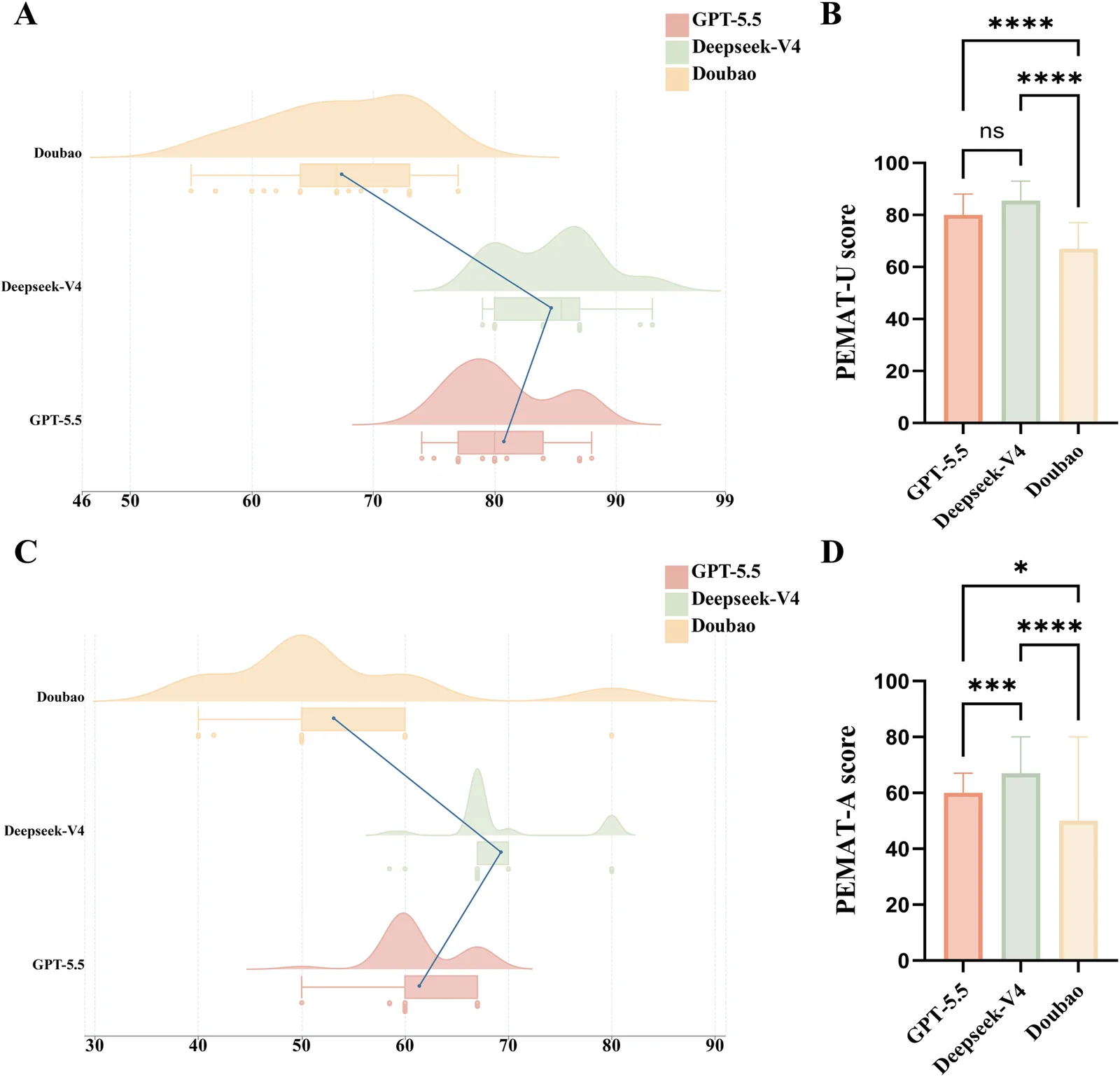
Distribution and comparison of understandability and actionability scores across the three LLM platforms. **(A)** Distribution of PEMAT-U scores. **(B)** Comparison of PEMAT-U scores. **(C)** Distribution of PEMAT-A scores. **(D)** Comparison of PEMAT-A scores.

### 3.7 Correlation analysis of key metrics

Significant positive correlations were observed among GQS, mDISCERN, QAMAI, EQIP, PEMAT-U, and PEMAT-A scores (*P* < 0.05). The readability indices ARI, GFI, FKGL, CLI, and SMOG were also positively correlated with one another (*P* < 0.05), whereas FRES was negatively correlated with ARI, GFI, FKGL, CLI, and SMOG (*P* < 0.05). In addition, GQS, mDISCERN, QAMAI, EQIP, PEMAT-U, and PEMAT-A showed either no significant associations or weak negative correlations with the readability indices (Figure 7).

**Figure 7:**
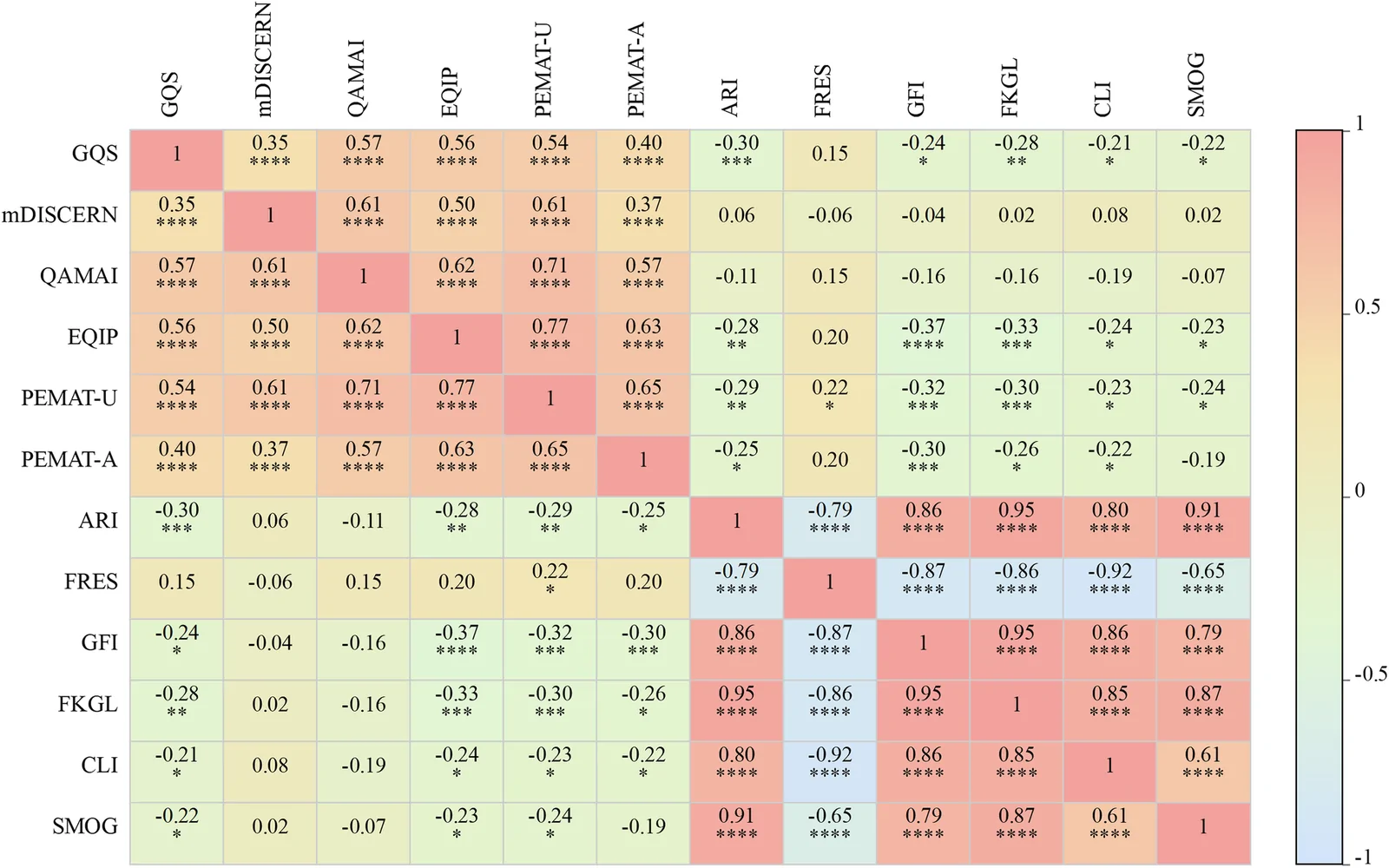
Spearman correlation matrix of quality, reliability, readability, understandability, and actionability metrics.

## 4 Discussion

This study evaluated the quality, reliability, readability, understandability, and actionability of responses generated by three large language models for esophageal cancer patient education. Overall, the generated responses demonstrated favorable quality and reliability, whereas readability and actionability remained notable limitations. DeepSeek showed superior performance in terms of response quality and reliability. In addition, no significant association was observed between the quality and reliability of LLM-generated responses and their readability. In the future, patients may be encouraged to use large language models as supplementary tools for obtaining basic health information about esophageal cancer; however, qualified healthcare professionals should still be consulted before making diagnostic or treatment-related decisions.

### 4.1 Deficiencies in response content

Content analysis of the 90 responses identified four categories of qualitative deficiencies, with crucial information missing being the most frequent overall**.** The models were generally able to provide first level, general information but tended to omit more specific, second level details. This pattern was most evident in diagnosis-related questions. For example, models frequently omitted recommendations for endoscopic screening in high-risk asymptomatic individuals (e.g., DeepSeek did not mention that high-risk individuals should consider endoscopic screening even without symptoms), the clinical applicability of PD-L1 CPS scoring in guiding immunotherapy decisions (e.g., neither ChatGPT nor Doubao mentioned CPS scoring), the essential role of molecular biomarker testing in treatment decision-making (e.g., Doubao did not mention HER2, PD-L1, or CLDN18.2 testing), and stage-specific survival reference data (e.g., Doubao only advised consulting a doctor without providing any stage-specific survival data). This pattern of “shallow coverage with deep omission” is not unique to the esophageal cancer domain. A MedBench-based evaluation of medical LLMs categorized response errors into eight types, identifying omissions as the most prevalent error type, with an occurrence rate of 96.3% in critical reasoning tasks (28). In head and neck oncology, a scoping review similarly found that patient-facing LLM outputs commonly suffer from omission of key clinical factors (29). Moreover, a large-scale evaluation of GPT-4.1 and DeepSeek-V3 in clinical guideline applications reported omission rates as high as 97% for DeepSeek-V3% and 46% for GPT-4.1 (30). These cross-cancer evidence suggest that the pattern of “shallow coverage with deep omission” is prevalent across LLM responses in oncology.

Other deficiencies, including factual inaccuracies, hallucinations, and provided outdated medical advice, were less common. However, their low frequency does not mean they are clinically negligible. Such errors included, but were not limited to, overly broad survival estimates without cited sources, overemphasis on single prognostic factors, and recommendations for interventions no longer supported by current evidence. These errors may lead patients to form inaccurate expectations about prognosis or to accept ineffective treatments. Therefore, even infrequent content deficiencies warrant verification against current guidelines before LLM outputs are used in patient education.

### 4.2 Quality, readability, and actionability: a mixed performance

#### 4.2.1 Acceptable quality with notable heterogeneity

Overall, the three LLMs demonstrated acceptable performance in quality and reliability for esophageal cancer patient education. The median scores for GQS and mDISCERN both reached relatively high levels, indicating that most responses met acceptable standards in overall quality and information reliability. These findings are consistent with previous observations in colorectal cancer (ChatGPT-4 GQS = 4.00) (7), endometrial cancer (ChatGPT and DeepSeek median GQS = 4.67 and 5.00) (31), and urological cancers (ChatGPT achieved GQS ≥5 in 64.6%–68.1% of questions) (32), suggesting that current mainstream LLMs have achieved a certain level of clinical usability in oncology patient education. However, the QAMAI and EQIP scores exhibited greater dispersion, indicating considerable heterogeneity across responses in structured presentation, evidence support, and practical guidance. Similar observations have been reported in myocardial infarction patient education, where EQIP revealed substantial inter-model variation despite comparable GQS scores (33). These findings suggest that LLMs may serve as supplementary sources of background information for patients; however, the observed heterogeneity in content quality and the lack of fine-grained guidance highlight the need for caution. These tools may be used for general disease education, whereas specific diagnostic and treatment decisions remain the responsibility of qualified healthcare professionals.

#### 4.2.2 The readability gap

Readability emerged as the most concerning finding of this study. All readability indices far exceeded the recommended sixth-grade reading level for patient education materials, corresponding to college-level reading difficulty. The American Medical Association and the National Institutes of Health explicitly recommend that patient-directed written materials should be at or below the sixth-grade level to promote patient understanding and improve health equity. Nevertheless, multiple prior studies have consistently demonstrated that both traditional online patient education materials and LLM-generated content universally exceed this standard (34, 35). This phenomenon may be attributed to several factors: LLM training corpora are predominantly composed of academic and encyclopedic texts; medical knowledge inherently possesses terminological density and conceptual hierarchy; and current mainstream LLMs lack adaptive readability control mechanisms for populations with limited health literacy. The significant variability in readability across responses further suggests a lack of consistency in the models' control over linguistic complexity. These findings underscore the need for future LLMs to optimize the readability of generated content, thereby improving the comprehensibility and accessibility of health information for effective application in patient education and health information dissemination.

#### 4.2.3 The understandability–actionability dissociation

For patient-centered metrics, the median PEMAT-U score was 80.00%, exceeding the recommended threshold of 70%, indicating that the generated content was generally understandable to patients. However, the median PEMAT-A score was only 60.00%, substantially below the recommended threshold, suggesting that the actionability of the recommendations was considerably deficient. This pattern of acceptable understandability yet insufficient actionability is not unique to this study. In research on retrograde cricopharyngeal muscle dysfunction, LLMs achieved a PEMAT understandability score of 79%, but actionability scores ranged from only 25% to 37% (36). In head and neck oncology, eight LLMs had a median PEMAT understandability score of 75.0 or higher, while the median PEMAT actionability score was 40 or lower (37). In dry eye disease, all LLM-generated materials met the understandability standard, yet none met the actionability threshold (38). This structural dissociation between understandability and actionability suggests that current LLMs prioritize information delivery over behavioral guidance. In the context of esophageal cancer, which is characterized by complex nutritional demands, multiple treatment pathways, and frequent emergencies, the gap between understanding and action may have tangible consequences for patient outcomes. Future model optimization efforts should therefore place greater emphasis on generating actionable content, such as specifying concrete steps, frequencies, and conditions for action, to bridge this dissociation and enhance the practical value of LLM-generated patient education materials.

### 4.3 Quality and readability gradients across models

The three models exhibited notable performance gradients across all evaluation metrics. In terms of quality and reliability, DeepSeek-V4 Flash achieved the highest scores on all four instruments, tying with ChatGPT on GQS and mDISCERN and ranking first outright on QAMAI and EQIP, followed by ChatGPT-5.5 Instant, with Doubao ranking lowest. DeepSeek's advantages were most evident in completeness and citation reliability. For diagnosis-related questions, DeepSeek systematically listed six categories of staging examinations and specified the role of each modality, whereas Doubao only briefly mentioned “staging examinations” without further elaboration. Similarly, for biomarker questions, DeepSeek explicitly distinguished the applicable scenarios for each biomarker and referenced guideline recommendations, while neither Doubao nor ChatGPT provided clear sources. ChatGPT-5.5 Instant's strengths lay in clarity and usefulness; it employed a progressive logical structure in responses to clinical manifestation questions and incorporated action-oriented statements in emergency scenarios, making the information easier for patients to understand and follow. These findings are consistent with broader evidence in medical AI evaluations. DeepSeek consistently outperforms comparator models in information completeness and citation reliability in breast cancer information retrieval, adverse event management, and anatomy question answering (39–41). Conversely, ChatGPT exhibits superior clarity and usefulness in patient education for lung cancer radiotherapy, cleft lip repair, and colorectal cancer (5, 7, 42). This performance differentiation may be attributed to differences in model architecture and training objectives. DeepSeek appears optimized for knowledge integration and evidence tracing, whereas ChatGPT prioritizes structured expression and patient-oriented actionable output. Doubao's substantial room for improvement across all dimensions suggests that model developers should focus on enhancing medical knowledge integration and structured output capabilities.

In terms of readability, DeepSeek achieved the highest FRES score, indicating relatively better readability among the three models, a finding also observed in ankylosing spondylitis patient education (43). However, even the best-performing model fell far below the sixth-grade reading level recommended by the American Medical Association. Doubao had the lowest FRES score and required the highest reading levels across all grade-level indices. DeepSeek exhibited the largest standard deviation in FRES, indicating the greatest variability in readability across different questions. This instability was also noted in breast cancer information retrieval (39), possibly reflecting the model's immature capacity to adaptively adjust linguistic style when handling questions of varying complexity. In contrast, Doubao demonstrated the greatest consistency in readability, but this was accompanied by the lowest FRES scores among the three models. This pattern may be partly attributable to the fact that Doubao was evaluated in English, whereas its primary user base is Chinese speaking. English only prompting may have placed Doubao at a disadvantage relative to its native language environment, and its lower quality and readability scores may partly reflect language mismatch rather than model capability alone. Notwithstanding this potential language related disadvantage, the consistently low readability across all three models indicates a shared limitation. These findings suggest that future LLM optimization should focus on improving both the stability and the overall level of readability while maintaining high-quality information output, particularly through the development of adaptive readability control mechanisms for populations with limited health literacy.

### 4.4 The independence of quality and readability

Correlation analysis revealed that readability and quality and reliability were largely independent dimensions. Most readability metrics showed no significant correlation with quality and reliability scores; the few exceptions were uniformly weak negative correlations. This pattern suggests that LLMs treat factual accuracy and linguistic complexity as separate objectives. The models are optimized for content completeness and evidence integration, while linguistic style is governed by a different set of mechanisms rooted in training data statistics. The lack of explicit coordination between these two processes allows responses to be informationally dense yet linguistically inaccessible, or simple to read yet lacking depth. This independence is not unique to esophageal cancer. Similar decoupling between quality and readability has been observed in gestational hypertension, skin cancer, and chronic heart failure patient education (44–46), suggesting that this is a general feature of LLM generated medical content. These findings challenge the assumption that improving readability necessarily comes at the cost of quality. Since the two dimensions can vary independently, there is no inherent trade off that precludes simultaneous optimization. This points toward strategies such as adaptive readability control mechanisms that adjust linguistic complexity based on patient health literacy levels, or the incorporation of patient centered evaluation metrics into the training pipeline. These approaches are theoretically feasible and not structurally precluded by current model architectures.

## 5 Limitations

First, this study had a cross-sectional design. Given the rapid and iterative evolution of LLMs, future longitudinal evaluations are needed to continuously monitor changes in performance across model updates and to identify emerging strengths and limitations in a timely manner. In addition, only three models were evaluated, and the knowledge cutoff dates and architectural details of some proprietary models were not fully disclosed, which may limit the generalizability of the findings. Finally, to control for baseline variability, all evaluations were conducted using English prompts. This English only design may limit the generalizability of the findings, particularly for Doubao, whose primary user base is Chinese speaking. Future studies should further examine whether these findings remain consistent across multilingual and cross-cultural settings.

## 6 Conclusions

This study systematically evaluated the performance of three mainstream large language models in esophageal cancer patient education. Although the models were able to provide basic information, they exhibited a common pattern of “shallow coverage with deep omission” and demonstrated consistently poor readability. No significant correlation was found between quality and readability, suggesting a structural tension between information accuracy and comprehensibility. Performance varied considerably across models: DeepSeek-V4 Flash led in quality and reliability, ChatGPT-5.5 Instant excelled in clarity and usability, while Doubao lagged behind across most dimensions. Overall, these tools have limited supplementary value and are not yet suitable for independent use. Future efforts should focus on improving information completeness and developing adaptive readability control mechanisms to better meet patient needs.

## Data Availability

The original contributions presented in the study are included in the article/Supplementary Material, further inquiries can be directed to the corresponding author/s.
