# Supplementary material for: Comparative evaluation of large language models for esophageal cancer patient education: quality, reliability, readability, understandability, and actionability
Source: Front Med (Lausanne). 2026 Oct 14;13:1973075. doi: 10.3389/fmed.2026.1973075 (PMC13713412; doi:10.3389/fmed.2026.1973075)
Supplement: Supplementary file 1 [file Table1.docx]

**Supplementary Table 1. Complete chatbot response set: verbatim outputs from Doubao, DeepSeek‑V4 Flash, and ChatGPT‑5.5 Instant for all 30 patient questions (90 responses in total). Responses are organized by model and question number.**

**ChatGPT**

Question1: What are the main risk factors for esophageal cancer?

Answer:
The main risk factors for esophageal cancer include the following:

1. Age and sex
   - The risk of esophageal cancer increases with age, and it is more common in people over 50 years old.
   - Men generally have a higher risk than women.
2. Smoking
   - Smoking is a major risk factor for esophageal cancer, particularly for esophageal squamous cell carcinoma.
3. Alcohol consumption
   - Heavy and long-term alcohol use increases the risk of esophageal cancer, especially when combined with smoking.
4. Dietary factors
   - Frequent consumption of pickled, smoked, or foods containing nitrosamines may increase risk.
   - Low intake of fruits and vegetables is also associated with a higher risk.
5. Obesity
   - Obesity, especially central obesity, increases the risk of esophageal adenocarcinoma.
6. Gastroesophageal reflux disease (GERD)
   - Chronic acid reflux can cause Barrett’s esophagus, which increases the risk of esophageal adenocarcinoma.
7. Barrett’s esophagus
   - Barrett’s esophagus is a precancerous condition caused by long-term acid exposure and is an important risk factor for esophageal adenocarcinoma.
8. Genetic factors and family history
   - A family history of esophageal cancer may increase an individual’s risk.
   - Certain inherited conditions can also increase susceptibility.
9. Esophageal disorders and chronic irritation
   - Conditions such as esophageal strictures, chronic inflammation, and long-term injury may increase the risk of malignant transformation.
10. Other factors

- Drinking very hot beverages may increase the risk of esophageal cancer.
- Nutritional deficiencies and environmental exposures in certain regions may also contribute to risk.

Question2: What are the differences in causes between esophageal squamous cell carcinoma and adenocarcinoma?

Answer:
Although esophageal squamous cell carcinoma (ESCC) and esophageal adenocarcinoma (EAC) both occur in the esophagus, their major causes and risk factors are different.

| Category | Esophageal Squamous Cell Carcinoma (ESCC) | Esophageal Adenocarcinoma (EAC) |
| --- | --- | --- |
| Common location | Middle and upper esophagus | Lower esophagus and gastroesophageal junction |
| Cell origin | Squamous epithelial cells lining the esophagus | Glandular cells or columnar cells arising from Barrett’s esophagus |
| Major risk factors | 1. Long-term tobacco smoking  2. Heavy alcohol consumption  3. Consumption of very hot beverages/foods  4. Nutritional deficiencies (low vitamins and micronutrients)  5. Exposure to carcinogens such as nitrosamines in preserved foods  6. Chronic irritation and inflammation of the esophagus | 1. Chronic gastroesophageal reflux disease (GERD)  2. Barrett’s esophagus (the most important precancerous condition)  3. Obesity, especially central obesity  4. Male sex and older age  5. Smoking  6. Long-term exposure to gastric acid and bile reflux |
| Main carcinogenic mechanism | Chronic chemical injury, tobacco/alcohol exposure, and inflammation cause genetic damage in squamous cells, leading to malignant transformation | Chronic acid reflux damages normal squamous epithelium, causing replacement by columnar epithelium (Barrett’s esophagus), which may progress to dysplasia and cancer |
| Geographic pattern | More common in China, Central Asia, and parts of Africa | More common in Western countries, especially Europe and North America |
| Associated conditions | Chronic esophagitis, nutritional deficiency, smoking and alcohol exposure | GERD and Barrett’s esophagus |

Summary:

- ESCC is mainly associated with tobacco, alcohol, dietary carcinogens, nutritional deficiency, and chronic irritation.
- EAC is mainly associated with gastroesophageal reflux disease, Barrett’s esophagus, and obesity.

Question3: What are the precancerous lesions of esophageal cancer?

Answer:
Precancerous lesions of esophageal cancer are abnormal pathological changes in the esophageal mucosa that have an increased risk of progressing to malignancy. The main precancerous lesions include:

1. Barrett’s esophagus
   - Usually associated with long-term gastroesophageal reflux disease (GERD).
   - Normal squamous epithelium in the lower esophagus is replaced by specialized intestinal metaplasia.
   - It is the major precancerous lesion for esophageal adenocarcinoma.
2. Esophageal squamous dysplasia
   - Characterized by abnormal proliferation and atypical changes of squamous epithelial cells.
   - It is classified as low-grade dysplasia or high-grade dysplasia depending on severity.
   - It is the major precancerous lesion for esophageal squamous cell carcinoma (ESCC).
3. Squamous epithelial metaplasia of the esophagus
   - Chronic irritation, such as tobacco use, alcohol consumption, consumption of very hot foods, and chronic inflammation, may cause abnormal epithelial changes.
   - Some cases may progress to dysplasia and eventually carcinoma.
4. Esophageal leukoplakia
   - Appears as localized white plaques on the esophageal mucosa.
   - Often associated with squamous epithelial hyperplasia and keratinization.
   - It carries a risk of malignant transformation.
5. Chronic inflammatory and injury-related esophageal lesions
   - Examples include chronic reflux esophagitis and caustic esophageal injury.
   - Persistent mucosal damage may increase the risk of esophageal cancer, particularly squamous cell carcinoma.

Summary:
The most important precancerous pathways are:

- Esophageal adenocarcinoma: Barrett’s esophagus → dysplasia → adenocarcinoma
- Esophageal squamous cell carcinoma: Squamous dysplasia → squamous cell carcinoma

Question4: Is esophageal cancer hereditary?

Answer:
Esophageal cancer is not typically considered a hereditary cancer, and most cases result from a combination of environmental factors, lifestyle factors, and chronic irritation. However, some individuals may have a genetic predisposition that increases their risk.

- Familial clustering: People with a first-degree relative (such as a parent or sibling) with esophageal cancer may have a higher risk, but this does not mean the disease is directly inherited.
- Inherited syndromes associated with increased risk: A small number of genetic disorders can increase the risk of esophageal cancer, including:
  - Fanconi anemia
  - Hereditary palmoplantar keratoderma (Tylosis, especially Howel-Evans syndrome), which is strongly associated with esophageal squamous cell carcinoma.
- Interaction between genes and environment: Certain genetic variations may make individuals more susceptible to carcinogens such as tobacco, alcohol, dietary factors, or other environmental exposures, increasing the likelihood of developing esophageal cancer.

Therefore, esophageal cancer is generally not directly inherited, but genetic factors can contribute to susceptibility and increase risk. Individuals with a strong family history or hereditary cancer syndromes may require closer surveillance and risk assessment.

Question5: What is the peak age of onset for esophageal cancer?

Answer:
The peak age of onset for esophageal cancer is usually between 60 and 70 years old, with most patients diagnosed at around 65 years of age. The risk of developing esophageal cancer increases with age, particularly after the age of 50. The age distribution may vary among different populations and regions. For example, esophageal squamous cell carcinoma commonly occurs in older adults in high-risk areas, while esophageal adenocarcinoma also mainly affects individuals over 60 years old.

Question6: What early symptoms of esophageal cancer should I be alert for?

Answer:
Early esophageal cancer often causes few noticeable symptoms and may be overlooked. Warning signs include:

1. Difficulty or discomfort when swallowing (dysphagia): Early symptoms may include a feeling that solid foods are sticking, slowing down, or not passing smoothly.
2. Discomfort or pain behind the breastbone: Some people may experience burning, pressure, or sharp pain in the chest, especially during eating.
3. A sensation of a lump or something stuck in the throat: Persistent throat discomfort without an obvious cause.
4. A feeling that food is delayed or trapped in the esophagus: A sensation that food does not move down normally after swallowing.
5. Unexplained weight loss: Losing weight without trying may be a warning sign.
6. Hoarseness, chronic cough, or repeated choking: These may occur if the tumor affects nearby structures.
7. Indigestion or acid reflux–like symptoms: Some patients may experience nonspecific digestive symptoms.

If you develop persistent swallowing difficulties, worsening swallowing problems, painful swallowing, or significant unexplained weight loss, you should seek medical evaluation. Tests such as upper endoscopy (gastroscopy) can help detect esophageal cancer, and early diagnosis improves treatment outcomes.

Question7: What are the clinical manifestations of esophageal cancer?

Answer:
The clinical manifestations of esophageal cancer depend on the tumor location, size, degree of invasion, and whether metastasis has occurred. Early-stage esophageal cancer often has no obvious symptoms, while symptoms may appear as the disease progresses.

1. Progressive dysphagia (the most common symptom)
   - Early symptoms may include mild discomfort or a sensation of something stuck when swallowing solid foods.
   - As the tumor grows, difficulty swallowing progresses from solid foods to soft foods and eventually even liquids.
2. Retrosternal discomfort or pain
   - Patients may experience burning, dull pain, sharp pain, or pressure behind the sternum.
   - Pain may worsen during swallowing.
3. Sensation of a foreign body or obstruction in the throat/chest
   - Patients may feel that food is blocked or does not pass smoothly through the esophagus.
4. Food regurgitation and vomiting
   - Narrowing of the esophagus can cause food retention, regurgitation, and sometimes vomiting.
5. Weight loss and malnutrition
   - Reduced food intake caused by swallowing difficulty may lead to weight loss, fatigue, and anemia.
6. Hoarseness
   - Tumor invasion or compression of the recurrent laryngeal nerve may cause persistent hoarseness.
7. Cough, breathing difficulties, or respiratory symptoms
   - Invasion of the trachea or bronchi, or formation of an esophagotracheal fistula, may cause coughing, choking, or shortness of breath.
8. Symptoms related to advanced metastasis
   - Advanced disease may cause enlarged cervical or supraclavicular lymph nodes, bone pain, jaundice, liver enlargement, or other metastatic symptoms.

Summary:
The hallmark symptom of esophageal cancer is progressive dysphagia. Persistent swallowing discomfort, difficulty swallowing, unexplained weight loss, or retrosternal pain should prompt medical evaluation, including endoscopy, for early detection and treatment.

Question8: How can I tell whether my esophageal cancer symptoms are worsening?

Answer:
Worsening symptoms of esophageal cancer may include the following signs:

1. Increasing difficulty swallowing (dysphagia): Difficulty progresses from solid foods to soft foods and eventually even liquids.
2. Worsening pain during swallowing (odynophagia): Pain behind the breastbone or along the esophagus becomes more frequent or severe when eating.
3. Unintentional weight loss: Continued weight loss without trying to lose weight may indicate reduced food intake or disease progression.
4. Reduced ability to eat or repeated vomiting: Difficulty swallowing may prevent normal eating and may cause food regurgitation or vomiting.
5. Increasing chest pain or discomfort: Persistent or worsening pain, pressure, or discomfort behind the breastbone.
6. Worsening hoarseness or cough: This may occur if the tumor affects nearby structures, such as the recurrent laryngeal nerve or airway.
7. Signs of bleeding or anemia: Vomiting blood, black stools, fatigue, dizziness, or pale skin may suggest bleeding.
8. Declining overall condition: Increasing weakness, fatigue, loss of appetite, or reduced quality of life may indicate disease progression.

Seek medical attention promptly if you experience rapidly worsening swallowing problems, inability to eat or drink, persistent vomiting of blood, breathing difficulties, severe chest pain, or rapid unexplained weight loss. Regular follow-up examinations, such as endoscopy and imaging studies, help determine whether the cancer is progressing or whether treatment is working.

Question9: Which organs does esophageal cancer most commonly spread to, and what symptoms occur after metastasis?

Answer:
Esophageal cancer has a high potential for invasion and metastasis. It can spread through lymphatic vessels, blood vessels, and direct invasion into nearby tissues. The most common metastatic sites include:

1. Lymph nodes (most common)
   - Esophageal cancer frequently spreads to cervical, mediastinal, perigastric, and abdominal lymph nodes.
   - Possible symptoms include:
     - A painless lump in the neck or above the collarbone;
     - Chest discomfort or pain;
     - Worsening difficulty swallowing;
     - Hoarseness due to involvement of the recurrent laryngeal nerve.
2. Liver
   - The liver is one of the most common sites of blood-borne metastasis.
   - Possible symptoms include:
     - Right upper abdominal discomfort or pain;
     - Loss of appetite and nausea;
     - Fatigue and weight loss;
     - Jaundice or abdominal fluid accumulation (ascites) in advanced cases.
3. Lungs
   - Lung metastasis is also relatively common.
   - Possible symptoms include:
     - Persistent cough;
     - Shortness of breath;
     - Chest pain;
     - Coughing up blood (in some cases).
4. Bones
   - Esophageal cancer may spread to the spine, pelvis, ribs, and other bones.
   - Possible symptoms include:
     - Persistent bone pain;
     - Limited movement;
     - Pathological fractures;
     - Numbness or weakness of the limbs if the spine is affected.
5. Brain (less common)
   - Brain metastasis is less frequent compared with liver, lung, and bone metastases.
   - Possible symptoms include:
     - Headaches;
     - Nausea and vomiting;
     - Vision or speech problems;
     - Weakness, seizures, or other neurological symptoms.

Advanced esophageal cancer may also directly invade nearby structures such as the trachea, bronchi, or aorta, causing:

- Severe cough or choking while eating;
- Breathing difficulties;
- Hoarseness;
- Chest pain.

Patients should seek medical evaluation if they develop rapidly worsening swallowing difficulty, unexplained weight loss, new persistent bone pain, jaundice, neurological symptoms, or breathing problems. Imaging tests such as CT, PET-CT, or MRI may be used to evaluate possible metastasis.

Question10: What emergency situations in patients with esophageal cancer require immediate visit to the emergency department?

Answer:
Patients with esophageal cancer should seek immediate emergency medical care if they experience any of the following situations:

1. Severe difficulty swallowing or complete inability to swallow
   - Inability to eat, drink, or even swallow saliva may indicate severe esophageal obstruction requiring urgent treatment.
2. Large amounts of vomiting blood or black stools
   - Vomiting bright red blood, coffee-ground material, or passing tarry black stools may indicate tumor-related bleeding and requires urgent evaluation.
3. Sudden chest pain or severe chest discomfort
   - Especially when accompanied by shortness of breath, sweating, palpitations, or altered consciousness, urgent assessment is needed to rule out serious complications such as esophageal perforation, infection, or cardiovascular emergencies.
4. Difficulty breathing, choking sensation, or persistent coughing
   - These symptoms may result from tumor compression of the airway, an esophagotracheal fistula, aspiration, or lung complications.
5. High fever, chills, or signs of infection
   - Persistent fever, chills, confusion, or worsening weakness may indicate a serious infection or sepsis requiring immediate treatment.
6. Severe abdominal pain, persistent vomiting, or inability to maintain food and fluid intake
   - These symptoms may suggest gastrointestinal obstruction, severe dehydration, or another acute abdominal condition.
7. Changes in consciousness or severe weakness
   - Confusion, loss of consciousness, extreme drowsiness, inability to stand, or sudden severe decline may indicate serious metabolic problems, infection, or disease progression.
8. Severe reactions after chemotherapy, radiotherapy, or immunotherapy
   - Emergency evaluation is needed for symptoms such as persistent high fever, severe diarrhea, significant bleeding, severe rash, breathing difficulty, or allergic reactions.
9. Sudden neurological symptoms
   - Weakness of an arm or leg, difficulty speaking, seizures, or severe headache may indicate brain metastasis or a stroke-like event and require urgent assessment.

If a patient with esophageal cancer develops any warning signs—especially inability to swallow, significant bleeding, breathing difficulty, altered consciousness, or severe pain—they should not wait for symptoms to improve on their own and should seek emergency medical care promptly.

Question11: Which department should I visit after being diagnosed with esophageal cancer?

Answer:
After being diagnosed with esophageal cancer, you should seek care from the oncology department, thoracic surgery department, or gastrointestinal surgery department (depending on the hospital’s organization) for further evaluation and treatment planning. The main departments involved include:

1. Thoracic Surgery / Gastrointestinal Surgery:
   - Evaluates whether the cancer can be surgically removed.
   - Patients with early-stage or locally advanced esophageal cancer may be considered for surgery, such as esophagectomy.
2. Medical Oncology:
   - Develops systemic treatment plans, including chemotherapy, immunotherapy, and targeted therapy.
   - This department plays a major role in treating advanced or unresectable esophageal cancer.
3. Radiation Oncology:
   - Provides assessment and treatment with radiotherapy.
   - Radiation therapy may be used before surgery, after surgery, or as the main treatment when surgery is not possible.
4. Multidisciplinary Team (MDT) Clinic:
   - Esophageal cancer treatment often requires collaboration among thoracic surgeons, oncologists, radiation oncologists, gastroenterologists, radiologists, and pathologists to create an individualized treatment plan.

If you have just been diagnosed with esophageal cancer, it is recommended to visit a hospital experienced in treating esophageal cancer, starting with the thoracic surgery or oncology department, and undergo appropriate staging evaluations (such as contrast-enhanced CT, PET-CT, or endoscopic ultrasound when indicated) to determine the most suitable treatment approach.

Question12: What is the gold standard for diagnosing esophageal cancer?

Answer:
The gold standard for diagnosing esophageal cancer is histopathological examination of biopsy specimens obtained during endoscopy (pathologic diagnosis).

Upper gastrointestinal endoscopy allows doctors to directly visualize abnormal areas in the esophagus, such as tumors, ulcers, strictures, or irregular mucosal changes, and obtain tissue samples for biopsy. Pathological examination confirms the presence of cancer cells and identifies the specific type of esophageal cancer (such as squamous cell carcinoma or adenocarcinoma), making it essential for definitive diagnosis.

Imaging studies, including contrast-enhanced CT, PET-CT, and endoscopic ultrasound, are mainly used to evaluate tumor invasion, lymph node involvement, and distant metastasis for staging purposes. However, they cannot replace pathological confirmation.

Question13: After being diagnosed with esophageal cancer, what additional tests are needed to determine the severity of the disease?

Answer:
After a diagnosis of esophageal cancer, additional staging tests are required to determine the extent of the tumor, whether lymph nodes or distant organs are involved, and the overall severity of the disease. These results help guide treatment decisions. Common tests include:

1. Contrast-enhanced CT scan of the chest and upper abdomen
   - Evaluates tumor size and whether the cancer has invaded surrounding structures.
   - Helps detect lymph node involvement and distant metastases in organs such as the lungs and liver.
2. Endoscopic ultrasound (EUS)
   - Assesses how deeply the tumor has invaded the layers of the esophageal wall (T stage).
   - Evaluates nearby lymph nodes for possible metastasis.
3. PET-CT (Positron Emission Tomography–Computed Tomography)
   - Helps identify hidden metastatic lesions throughout the body.
   - Is useful for assessing distant metastasis (M stage), especially in patients suspected of having advanced disease or those being considered for surgery.
4. Bronchoscopy (for selected patients)
   - May be recommended for tumors located in the upper or middle esophagus or when invasion of the trachea or bronchi is suspected.
5. Pathological examination and molecular testing
   - Confirms the cancer type (such as squamous cell carcinoma or adenocarcinoma) and tumor differentiation.
   - In advanced cases, tests such as HER2, PD-L1, and MSI may be performed to guide targeted therapy or immunotherapy.
6. Blood tests and overall health assessment
   - Includes complete blood count, liver and kidney function tests, electrolyte levels, and coagulation tests.
   - Evaluates nutritional status and the patient’s ability to tolerate surgery, chemotherapy, radiotherapy, or other treatments.

Based on these results, doctors determine the cancer stage using the TNM staging system (T = tumor invasion, N = lymph node involvement, M = distant metastasis). The stage helps classify the disease as early, intermediate, or advanced and guides individualized treatment planning.

Question14: What stage is my esophageal cancer?

Answer:
The stage of esophageal cancer cannot be determined based only on symptoms or endoscopy findings. Doctors determine the stage using the TNM staging system, which evaluates:

- T (Tumor): how deeply the cancer has invaded the esophageal wall or nearby tissues
- N (Nodes): whether and how many regional lymph nodes are involved
- M (Metastasis): whether the cancer has spread to distant organs

The general stages are:

- Stage 0 (carcinoma in situ): Cancer cells are limited to the superficial lining of the esophagus and have not invaded deeper layers.
- Stage I: An early-stage cancer usually confined to the esophageal wall, with no or minimal lymph node involvement.
- Stage II: The tumor has deeper invasion and/or limited regional lymph node involvement.
- Stage III: The cancer has invaded nearby structures or has more extensive regional lymph node involvement.
- Stage IV: The cancer has spread to distant organs, such as the liver, lungs, bones, or brain.

To determine the exact stage, doctors usually use:

- Upper endoscopy with biopsy (to confirm cancer type and characteristics)
- Contrast-enhanced CT of the chest and abdomen (to assess invasion and metastasis)
- PET-CT in selected patients (to detect hidden metastases)
- Endoscopic ultrasound (to evaluate depth of tumor invasion)
- Bronchoscopy in some patients with upper or middle esophageal tumors
- Evaluation of lymph nodes or metastatic lesions

Please provide your pathology report, imaging results (CT/PET-CT), and tumor location if you want a more accurate assessment of your specific esophageal cancer stage.

Question15: What do the HER2, PD-L1, dMMR/MSI, and Claudin18.2 indicators mean in an esophageal cancer pathology report?

Answer:
The HER2, PD-L1, dMMR/MSI, and Claudin18.2 indicators in an esophageal cancer pathology report are molecular biomarkers used to describe tumor characteristics and help determine whether a patient may benefit from targeted therapy or immunotherapy.

1. HER2 (Human Epidermal Growth Factor Receptor 2)

Meaning:
HER2 is a protein involved in cell growth signaling. Some cancers have HER2 overexpression or HER2 gene amplification, which may drive tumor growth.

Clinical significance:

- HER2-positive: Some patients, especially those with esophageal adenocarcinoma or gastroesophageal junction adenocarcinoma, may benefit from HER2-targeted therapy.
- HER2-negative: HER2-targeted drugs are generally less likely to be effective.

Common tests:

- Immunohistochemistry (IHC): Measures HER2 protein expression.
- ISH/FISH: Detects HER2 gene amplification.

2. PD-L1 (Programmed Death-Ligand 1)

Meaning:
PD-L1 is a protein expressed on tumor cells or immune cells that can help cancer cells evade immune system attacks.

Clinical significance:

- Higher PD-L1 expression may indicate a greater chance of benefit from immune checkpoint inhibitors (such as PD-1/PD-L1 inhibitors).
- PD-L1 negativity does not always mean immunotherapy cannot be used; treatment decisions depend on the cancer type, stage, and other factors.

Common scoring methods:

- CPS (Combined Positive Score): Commonly used in esophageal and gastroesophageal cancers to evaluate immunotherapy eligibility.
- TPS (Tumor Proportion Score): Measures the percentage of PD-L1-positive tumor cells.

3. dMMR / MSI (Mismatch Repair Deficiency / Microsatellite Instability)

Meaning:

- MMR: A DNA repair system that corrects genetic errors.
- dMMR: Indicates loss of function of this repair system.
- MSI: Indicates instability in repetitive DNA sequences caused by repair defects.

Clinical significance:

- Tumors with dMMR or MSI-high status often contain more mutations and may be more easily recognized by the immune system.
- These tumors may respond better to immunotherapy.

Common tests:

- Immunohistochemistry for MMR proteins (MLH1, MSH2, MSH6, PMS2).
- MSI molecular testing.

4. Claudin18.2 (CLDN18.2)

Meaning:
Claudin18.2 is a protein involved in cell junctions. It is usually limited in normal tissues but may be highly expressed in some gastric cancers, gastroesophageal junction cancers, and some esophageal adenocarcinomas.

Clinical significance:

- Patients with high Claudin18.2 expression may be candidates for Claudin18.2-targeted therapies.
- It is mainly used to identify patients who may benefit from emerging targeted treatments.

Testing method:

- Immunohistochemistry (IHC) to evaluate the proportion and intensity of Claudin18.2-positive tumor cells.

Summary Table:

| Marker | Main Purpose | Possible Meaning if Positive |
| --- | --- | --- |
| HER2 | Determines eligibility for HER2-targeted therapy | May benefit from anti-HER2 treatment |
| PD-L1 | Predicts potential benefit from immunotherapy | May have higher likelihood of response to immunotherapy |
| dMMR/MSI | Indicates tumor immune characteristics | May be more sensitive to immunotherapy |
| Claudin18.2 | Identifies candidates for new targeted therapies | May qualify for Claudin18.2-targeted treatment |

Important: These biomarkers do not determine treatment decisions by themselves. Doctors also consider the esophageal cancer subtype (squamous cell carcinoma vs adenocarcinoma), TNM stage, overall health status, and other molecular findings when developing an individualized treatment plan.

Question16: Is endoscopic resection suitable for early-stage esophageal cancer?

Answer:
Endoscopic resection (ER) can be a suitable treatment option for some patients with early-stage esophageal cancer. However, eligibility depends on factors such as the depth of tumor invasion, histological type, tumor differentiation, and the risk of lymph node metastasis.

Endoscopic resection is generally considered appropriate when:

- The cancer is confined to the mucosal layer of the esophagus (T1a stage), especially when limited to the epithelium or lamina propria;
- The lesion is small, clearly defined, and can be completely removed endoscopically;
- There is no evidence of lymph node metastasis;
- The tumor has favorable pathological features, such as good differentiation and low risk of spread.

Common endoscopic treatment methods include:

- Endoscopic mucosal resection (EMR): usually used for smaller and superficial lesions;
- Endoscopic submucosal dissection (ESD): often preferred for larger lesions or when en bloc removal is needed for accurate pathological evaluation.

If the cancer has invaded the submucosal layer (T1b stage), the risk of lymph node metastasis increases. In such cases, endoscopic resection alone may not be sufficient, and additional treatment options such as surgery or combined therapies may need to be considered.

After endoscopic resection, the final pathology report— including invasion depth, resection margins, lymphovascular invasion, and tumor differentiation—determines whether additional treatment or close surveillance is required.

Therefore, not all early esophageal cancers require surgery. Patients who meet appropriate criteria may achieve curative treatment through endoscopic resection, but the decision should be made through multidisciplinary evaluation involving gastroenterologists, endoscopy specialists, thoracic surgeons, and oncologists.

Question17: What are the surgical approaches for esophageal cancer?

Answer:
The choice of surgical approach for esophageal cancer depends on tumor location, cancer stage, patient health condition, and whether additional treatments are required. Common surgical approaches include:

1. Esophagectomy
   - This is the main curative surgical treatment for resectable esophageal cancer.
   - The procedure usually involves removing the tumor-containing portion of the esophagus, performing lymph node dissection, and reconstructing the digestive tract (most commonly by pulling the stomach up and connecting it to the remaining esophagus).
2. Transthoracic Esophagectomy
   - The surgery is performed through the chest and abdomen.
   - It is commonly used for tumors located in the middle or lower thoracic esophagus.
   - It allows extensive lymph node removal in the chest.
   - Common techniques include:
     - Ivor Lewis Esophagectomy: abdominal and right chest approaches with an intrathoracic stomach–esophagus connection.
     - McKeown Esophagectomy (Three-incision Esophagectomy): abdominal, right chest, and neck incisions, often used when a higher anastomosis is needed.
3. Transhiatal Esophagectomy
   - The esophagus is removed through abdominal and neck incisions without opening the chest.
   - It may be suitable for some lower esophageal tumors.
   - It may result in less surgical trauma, although lymph node dissection in the chest may be more limited.
4. Minimally Invasive Esophagectomy (MIE)
   - Includes laparoscopic, thoracoscopic, and robot-assisted esophagectomy.
   - Compared with open surgery, it may provide smaller incisions, less postoperative pain, and faster recovery.
   - Suitability depends on tumor characteristics, cancer stage, and the experience of the surgical team.
5. Endoscopic Resection
   - Mainly used for very early-stage esophageal cancer, such as lesions limited to the mucosa or selected superficial submucosal cancers without significant risk of lymph node spread.
   - Common techniques include:
     - Endoscopic Mucosal Resection (EMR)
     - Endoscopic Submucosal Dissection (ESD)
   - In appropriately selected patients, endoscopic treatment can be curative.
6. Palliative or Symptom-Relieving Procedures
   - For advanced cancers that cannot be completely removed or patients with severe swallowing difficulty, procedures may include:
     - Esophageal stent placement
     - Gastrostomy or jejunostomy for nutritional support
     - Bypass procedures to improve food passage
   - The goal is mainly to relieve symptoms and improve quality of life.

Summary:
Surgical options for esophageal cancer include endoscopic resection, open or minimally invasive esophagectomy, and palliative procedures. Early-stage cancers may be treated with endoscopic methods, while localized resectable cancers are usually treated with esophagectomy plus lymph node dissection. More advanced cases often require a multidisciplinary approach combining surgery with chemotherapy, radiotherapy, or immunotherapy when appropriate.

Question18: Why is chemoradiotherapy needed before surgery for esophageal cancer?

Answer:
Chemoradiotherapy before surgery, also called neoadjuvant chemoradiotherapy, is mainly used to improve treatment outcomes and reduce the risk of recurrence after surgery. Its benefits include:

1. Shrinking the tumor and improving the chance of complete surgical removal
   Radiation and chemotherapy can reduce tumor size and decrease tumor invasion into surrounding tissues or critical structures, making complete resection more achievable.
2. Eliminating microscopic metastatic disease
   Esophageal cancer cells may have already spread in small amounts before they can be detected by imaging. Preoperative chemotherapy may destroy these hidden cancer cells and reduce the risk of distant recurrence.
3. Reducing the risk of local recurrence
   Radiation therapy can target cancer cells around the primary tumor area, lowering the chance that cancer will return locally after surgery.
4. Assessing tumor response to treatment
   The response to preoperative therapy provides information about tumor biology and helps doctors plan further treatment strategies.
5. Improving long-term outcomes in selected patients
   For patients with locally advanced esophageal cancer (such as some stage II and stage III cases), neoadjuvant chemoradiotherapy followed by surgery can provide better chances of disease control and improved long-term survival compared with surgery alone.

It is important to note that not every patient with esophageal cancer requires preoperative chemoradiotherapy. The treatment plan depends on factors such as tumor stage, pathological type (such as squamous cell carcinoma or adenocarcinoma), overall health condition, and evaluation by a multidisciplinary medical team.

Question19: What are the most commonly used chemotherapy, immunotherapy, and targeted therapy drugs for advanced esophageal cancer?

Answer:
Treatment of advanced esophageal cancer usually involves systemic therapies, including chemotherapy, immunotherapy, and targeted therapy. The choice of treatment depends on the tumor type (esophageal squamous cell carcinoma or adenocarcinoma), disease stage, overall health condition, and molecular testing results such as HER2 and PD-L1 status.

1. Commonly used chemotherapy drugs

Common chemotherapy agents for advanced esophageal cancer include:

Platinum-based drugs

- Cisplatin
- Oxaliplatin
- Carboplatin

Fluoropyrimidine drugs

- 5-Fluorouracil (5-FU)
- Capecitabine

Taxane drugs

- Paclitaxel
- Docetaxel

Other chemotherapy drugs

- Irinotecan
- Gemcitabine

Common combination regimens include:

- Cisplatin + 5-FU
- Oxaliplatin + Capecitabine
- Paclitaxel + Platinum-based chemotherapy

2. Commonly used immunotherapy drugs

Immunotherapy works by blocking the PD-1/PD-L1 pathway, allowing the immune system to better recognize and attack cancer cells.

Common immune checkpoint inhibitors include:

- Pembrolizumab
- Nivolumab
- Camrelizumab
- Tislelizumab
- Sintilimab

Immunotherapy is often combined with chemotherapy, especially for:

- Advanced or metastatic esophageal squamous cell carcinoma
- Patients with higher PD-L1 expression
- Selected patients with esophageal adenocarcinoma

3. Commonly used targeted therapy drugs

Targeted therapy is selected based on specific molecular abnormalities in the tumor.

For HER2-positive esophageal adenocarcinoma:

- Trastuzumab
- Trastuzumab combined with chemotherapy
- Other HER2-targeted agents, such as trastuzumab deruxtecan (T-DXd), in selected situations

Anti-angiogenic targeted therapy:

- Apatinib
- Other VEGF/VEGFR pathway inhibitors (depending on regional approval and clinical circumstances)

4. How is treatment selected?

Doctors consider multiple factors, including:

- Tumor type: squamous cell carcinoma or adenocarcinoma
- Presence of distant metastases
- PD-L1 expression level (such as CPS score)
- HER2 status
- MSI/dMMR status
- Patient’s age, nutritional status, and ability to tolerate treatment

Currently, the preferred approach for many patients with advanced esophageal cancer is chemotherapy combined with immunotherapy. For patients with specific molecular targets, targeted therapy may be added to improve treatment outcomes.

Question20: What treatment options are available for esophageal cancer from early to advanced stages?

Answer:
Treatment options for esophageal cancer depend mainly on the cancer stage, tumor location, histological type (such as squamous cell carcinoma or adenocarcinoma), the patient’s overall health, and molecular testing results. Available treatments include endoscopic therapy, surgery, radiotherapy, chemotherapy, immunotherapy, and targeted therapy.

1. Early-stage esophageal cancer (limited to the mucosa or superficial layers)

Main goal: Cure the cancer while preserving esophageal function whenever possible.

- Endoscopic resection:
  - Suitable for selected early cancers confined to the mucosal layer with a low risk of lymph node spread.
  - Common procedures include endoscopic mucosal resection (EMR) and endoscopic submucosal dissection (ESD).
  - Advantages include less trauma, faster recovery, and avoidance of major surgery.
- Surgical resection:
  - For tumors invading deeper layers or with a higher risk of lymph node involvement, esophagectomy with lymph node dissection may be recommended.

2. Locally advanced esophageal cancer (usually stage II–III)

Main goal: Improve the chance of cure and reduce recurrence risk.

- Neoadjuvant chemoradiotherapy followed by surgery:
  - Chemotherapy combined with radiation therapy is given before surgery to shrink the tumor and reduce local recurrence.
  - Commonly used for selected patients with resectable locally advanced disease.
- Neoadjuvant chemotherapy followed by surgery:
  - Preoperative chemotherapy helps control microscopic disease and improve the chance of complete tumor removal.
  - Used in certain patients depending on tumor type and clinical guidelines.
- Definitive chemoradiotherapy:
  - For patients who cannot undergo surgery or are not suitable surgical candidates, combined chemotherapy and radiation may be used as the primary treatment.
- Adjuvant therapy after surgery:
  - Depending on surgical pathology results, some patients may receive additional chemotherapy or immunotherapy to reduce recurrence risk.

3. Advanced or metastatic esophageal cancer (stage IV)

Main goal: Control disease progression, prolong survival, and improve quality of life.

- Systemic drug therapy:

Chemotherapy:

- Common regimens include platinum-based drugs combined with fluoropyrimidines or taxanes.

Immunotherapy:

- PD-1/PD-L1 inhibitors (such as nivolumab and pembrolizumab) may be used in selected patients, especially those with positive PD-L1 expression.

Targeted therapy:

- Patients with HER2-positive esophageal adenocarcinoma may benefit from anti-HER2 therapy.
- Other targeted treatments depend on molecular and genetic testing results.
- Palliative local treatments:
  - Radiotherapy: Helps relieve symptoms such as difficulty swallowing, pain, or bleeding.
  - Esophageal stent placement: Helps restore swallowing when tumors cause severe narrowing.
  - Nutritional support: Includes enteral feeding, feeding tubes, or intravenous nutrition when needed.

4. Recurrent esophageal cancer

Treatment depends on the location of recurrence and previous therapies:

- Repeat surgery in selected patients.
- Radiotherapy or chemotherapy.
- Immunotherapy.
- Targeted therapy.
- Clinical trials.

Summary

| Disease stage | Common treatment options |
| --- | --- |
| Early stage | Endoscopic resection, surgery |
| Locally advanced stage | Neoadjuvant chemoradiotherapy/chemotherapy + surgery, or definitive chemoradiotherapy |
| Advanced metastatic stage | Chemotherapy, immunotherapy, targeted therapy, supportive care |
| Recurrent disease | Individualized multidisciplinary treatment |

Management of esophageal cancer usually requires a multidisciplinary team (MDT), including thoracic surgeons, medical oncologists, radiation oncologists, gastroenterologists, and pathologists, to develop the most appropriate treatment plan for each patient.

Question21: On what basis does the doctor formulate my treatment plan for esophageal cancer?

Answer:
Doctors develop an individualized treatment plan for esophageal cancer based on multiple factors, including:

1. Cancer stage (the most important factor)
   - The treatment decision is mainly guided by the TNM stage:
     - T stage: depth of tumor invasion into the esophageal wall;
     - N stage: whether lymph nodes are involved;
     - M stage: whether there is distant metastasis.
   - Early-stage disease may be treated mainly with endoscopic resection or surgery.
   - Locally advanced disease may require neoadjuvant chemoradiotherapy or chemotherapy followed by surgery.
   - Advanced metastatic disease is usually treated with systemic therapy, such as chemotherapy, immunotherapy, or targeted therapy.
2. Pathological type and tumor characteristics
   The doctor considers the type of esophageal cancer, mainly:
   - Esophageal squamous cell carcinoma (ESCC)
   - Esophageal adenocarcinoma (EAC)

Different pathological types may respond differently to treatments.

3. Molecular and biomarker test results
   Pathology and molecular testing may provide important treatment guidance, including:
   - HER2 status: helps determine whether anti-HER2 targeted therapy may be appropriate;
   - PD-L1 expression (such as CPS score): helps predict the potential benefit from immunotherapy;
   - dMMR/MSI status: helps assess sensitivity to immune checkpoint inhibitors;
   - Other molecular markers when clinically indicated.
4. Tumor location and extent
   Doctors consider:
   - Whether the tumor is located in the upper, middle, or lower esophagus;
   - Whether it has invaded surrounding tissues or critical structures;
   - Whether complete surgical removal is possible.
5. The patient’s overall health condition
   The doctor evaluates:
   - Age;
   - Heart and lung function;
   - Liver and kidney function;
   - Nutritional status and weight loss;
   - Ability to tolerate surgery, radiation therapy, or systemic treatments.
6. The patient’s preferences and treatment goals
   Treatment decisions also depend on:
   - Whether the goal is cure or disease control;
   - The patient’s tolerance for treatment side effects;
   - Quality-of-life considerations;
   - The patient’s personal preferences.
7. Multidisciplinary team (MDT) discussion
   Esophageal cancer treatment is often planned through an MDT approach involving:
   - Thoracic surgeons;
   - Medical oncologists;
   - Radiation oncologists;
   - Gastroenterologists;
   - Radiologists;
   - Pathologists.

Summary:
Doctors formulate an esophageal cancer treatment plan mainly based on cancer stage, pathological type, molecular test results, tumor location, overall health condition, and the patient’s treatment goals. Accurate staging and complete diagnostic evaluation are essential for selecting the most appropriate treatment strategy.

Question22: What is the probability of cure for early-stage esophageal cancer?

Answer:
The chance of cure for early-stage esophageal cancer is relatively high, but it depends on factors such as cancer stage, depth of invasion, tumor type, treatment method, and the patient’s overall health condition.

- Very early esophageal cancer (limited to the mucosal layer without invasion into deeper layers):
  If completely removed by endoscopic treatment, such as endoscopic mucosal resection (EMR) or endoscopic submucosal dissection (ESD), the long-term cure rate can generally be above 90%.
- Early-stage esophageal cancer (Stage I, confined to the esophagus without lymph node metastasis):
  After curative surgery or standard treatment, the 5-year survival rate is usually around 70%–90%, and many patients can achieve long-term disease control or clinical cure.
- Major factors affecting the probability of cure include:
  1. Depth of tumor invasion: More superficial tumors have a better chance of cure.
  2. Presence of lymph node metastasis: Patients without lymph node involvement generally have a much better prognosis.
  3. Pathological type: Prognosis may differ between esophageal squamous cell carcinoma and adenocarcinoma.
  4. Completeness and appropriateness of treatment: Including complete tumor removal and additional treatments such as chemotherapy or radiotherapy when needed.
  5. Overall health status and follow-up care.

It is important to understand that “cure” usually means long-term absence of disease recurrence. In oncology, 5-year recurrence-free survival or long-term survival is often used as an important reference point. Even after successful treatment of early-stage esophageal cancer, regular follow-up examinations are still necessary because a small number of patients may experience recurrence.

Question23: What is the approximate survival rate for my stage of esophageal cancer?

Answer:
The survival rate of esophageal cancer depends mainly on the exact stage (Stage 0, I, II, III, or IV), tumor location, pathological type (squamous cell carcinoma or adenocarcinoma), whether complete surgical removal is possible, treatment response, and overall health condition. Since your exact stage has not been provided, the following are general estimates:

- Stage 0 (carcinoma in situ / very early disease):
  After endoscopic resection or surgery, the 5-year survival rate is usually over 90%, and many patients can achieve long-term cure.
- Stage I (early-stage esophageal cancer):
  When the tumor is confined to the esophageal wall and can be completely removed, the 5-year survival rate is approximately 70%–90%. Some very early cases treated with endoscopic therapy have excellent outcomes.
- Stage II:
  Treatment usually involves surgery combined with chemotherapy and/or radiotherapy. The 5-year survival rate is approximately 40%–70%, depending largely on whether lymph nodes are involved.
- Stage III:
  Many patients require combined treatment, such as neoadjuvant chemoradiotherapy followed by surgery. The 5-year survival rate is approximately 20%–50%. Patients who respond well to treatment and undergo successful surgery may still achieve long-term survival.
- Stage IV (metastatic esophageal cancer):
  Treatment is mainly systemic therapy, including chemotherapy, immunotherapy, and targeted therapy. The overall 5-year survival rate is generally below 10%–20%, although some patients who respond well to newer immunotherapy approaches may experience prolonged disease control.

It is important to understand that survival statistics represent averages from large groups of patients and cannot accurately predict an individual’s outcome. For example, two patients with the same stage III cancer may have very different outcomes depending on lymph node involvement, tumor biology, treatment response, and whether curative surgery is possible.

If you provide:

1. Your exact cancer stage (for example, T2N1M0 or Stage IIIA);
2. Pathological type (squamous cell carcinoma or adenocarcinoma);
3. Whether you have had surgery or are receiving chemoradiotherapy, immunotherapy, or targeted therapy;

I can help estimate a survival range that is more specific to your situation.

Question24: Can esophageal cancer recur after treatment is completed?

Answer:
Yes. Even after completing standard treatments such as surgery, radiotherapy, chemotherapy, or immunotherapy, esophageal cancer can still recur. However, the risk of recurrence varies among patients and depends on several factors:

1. Cancer stage:
   - Early-stage esophageal cancer, which is limited to the mucosa or superficial layers, generally has a lower risk of recurrence after curative treatment.
   - Locally advanced or advanced esophageal cancer has a higher risk because microscopic cancer cells may remain or spread beyond what can be detected.
2. Pathological features:
   - Factors such as depth of tumor invasion, lymph node involvement, surgical margin status (whether the tumor was completely removed), and tumor differentiation affect recurrence risk.
   - More lymph node metastases usually indicate a higher chance of recurrence.
3. Treatment effectiveness:
   - Achieving complete surgical removal (R0 resection), response to preoperative chemoradiotherapy, and completion of recommended postoperative treatments can influence long-term outcomes.
4. Common sites of recurrence:
   - Local recurrence: Tumor returns near the original esophagus or surgical connection site.
   - Regional recurrence: Cancer returns in nearby lymph nodes.
   - Distant metastasis: Cancer spreads to organs such as the liver, lungs, bones, or brain.
5. Treatment after recurrence:
   Recurrence does not always mean that treatment options are exhausted. Doctors will choose treatment based on the location and extent of recurrence and the patient’s overall condition. Options may include additional surgery, radiotherapy, chemotherapy, immunotherapy, or targeted therapy.

Therefore, long-term follow-up is essential after completing treatment for esophageal cancer. Regular endoscopy, imaging studies, and appropriate blood tests can help detect recurrence early and improve the chances of timely treatment.

Question25: What major factors affect the long-term prognosis of esophageal cancer patients?

Answer:
The long-term prognosis of patients with esophageal cancer, including survival, risk of recurrence, and quality of life, is influenced by multiple factors. The major factors include:

1. Cancer stage (the most important factor)
   - The stage at diagnosis is the strongest predictor of long-term outcome.
   - Early-stage esophageal cancer confined to the mucosa or esophageal wall generally has a better chance of long-term survival after treatment.
   - Advanced disease with lymph node involvement or distant metastasis has a higher risk of recurrence and poorer survival.
2. Depth of tumor invasion
   - The deeper the tumor grows into the esophageal wall, the more advanced the disease tends to be.
   - Tumor invasion into the outer esophageal layer or nearby organs is associated with a worse prognosis.
3. Lymph node involvement
   - Whether lymph nodes are involved, as well as the number and extent of involved nodes, are important predictors of recurrence.
   - More extensive lymph node metastasis usually indicates a higher risk of disease recurrence.
4. Presence of distant metastasis
   - Spread to organs such as the liver, lungs, bones, or brain significantly affects long-term outcomes.
   - Patients without distant metastasis generally have more opportunities for curative treatment.
5. Tumor pathology and differentiation
   - Esophageal squamous cell carcinoma and esophageal adenocarcinoma have different biological behaviors.
   - Well-differentiated tumors usually have a better prognosis, while poorly differentiated tumors tend to be more aggressive.
6. Treatment strategy and treatment response
   - Receiving standardized comprehensive treatment, including surgery, chemotherapy, radiotherapy, immunotherapy, or targeted therapy when appropriate, can influence long-term outcomes.
   - Complete tumor removal (such as R0 resection after surgery) and response to additional treatments are important prognostic factors.
7. Molecular and biomarker characteristics
   - Biomarkers such as HER2, PD-L1, MSI/dMMR, and Claudin18.2 may guide the use of targeted therapies or immunotherapies.
   - For some advanced patients, personalized treatment based on these markers may improve disease control.
8. Patient’s overall health condition
   - Age, nutritional status, weight changes, heart and lung function, and other medical conditions can affect treatment tolerance and recovery.
   - Maintaining good nutrition and physical condition can improve the ability to complete treatment.
9. Follow-up care after treatment
   - Regular surveillance helps detect recurrence or metastasis at an earlier stage.
   - Managing swallowing difficulties, nutritional problems, and treatment-related complications can improve quality of life.

Summary:
The major factors affecting long-term prognosis in esophageal cancer include tumor stage, lymph node and distant metastasis status, pathological characteristics, effectiveness of treatment, and the patient’s overall health condition. Early detection, accurate staging, standardized treatment, and long-term follow-up are essential for improving outcomes.

Question26: What are the common side effects during chemotherapy, radiotherapy, targeted therapy, or immunotherapy for esophageal cancer, and how should I manage them?

Answer:
Different treatments for esophageal cancer may cause different side effects. Most treatment-related adverse effects can be prevented or relieved through early monitoring, supportive care, and appropriate medical management.

1. Common Side Effects of Chemotherapy and Management

(1) Nausea, vomiting, and loss of appetite

- Management:
  - Take prescribed anti-nausea medications;
  - Eat small, frequent meals with high-protein and easy-to-digest foods;
  - Avoid greasy foods and strong odors;
  - Maintain adequate fluid intake.

(2) Bone marrow suppression (low white blood cells, red blood cells, or platelets)

- Possible symptoms: Increased infection risk, fatigue, anemia, bruising, or bleeding.
- Management:
  - Regular blood tests;
  - Use growth-factor medications if recommended by the doctor;
  - Practice good hand hygiene and avoid infection exposure;
  - Avoid injury when platelet counts are low.

(3) Hair loss, fatigue, and mouth sores

- Management:
  - Maintain good oral hygiene and use gentle mouth rinses;
  - Balance rest with appropriate physical activity;
  - Hair usually grows back after treatment completion.

2. Common Side Effects of Radiotherapy and Management

(1) Radiation esophagitis (painful swallowing and chest discomfort)

- Management:
  - Eat soft, liquid, or semi-liquid foods;
  - Avoid spicy, very hot, or rough-textured foods;
  - Use pain medication or mucosal protective agents if needed.

(2) Fatigue

- Management:
  - Get adequate sleep;
  - Perform light activities according to physical ability;
  - Arrange treatment and rest periods appropriately.

(3) Skin reactions in the radiation area

- Management:
  - Keep the skin clean and dry;
  - Avoid rubbing, sun exposure, and irritating products;
  - Use recommended skin-care medications when needed.

3. Common Side Effects of Targeted Therapy and Management

(1) Skin rash, dry skin, and hand-foot syndrome

- Management:
  - Apply moisturizers regularly;
  - Avoid excessive heat and friction;
  - Inform the doctor if skin reactions become severe.

(2) Diarrhea

- Management:
  - Maintain hydration and electrolyte balance;
  - Avoid cold, greasy, or irritating foods;
  - Seek medical advice if diarrhea is severe or persistent.

(3) Liver function abnormalities

- Management:
  - Monitor liver function tests regularly;
  - Avoid alcohol and unnecessary medications that may affect the liver;
  - Treatment adjustments may be required based on test results.

4. Common Side Effects of Immunotherapy and Management

Immunotherapy may cause immune-related adverse events (irAEs) because the immune system can attack normal tissues.

(1) Skin reactions (rash and itching)

- Management:
  - Use anti-allergy medications or topical treatments when appropriate;
  - Severe reactions require medical evaluation.

(2) Thyroid or other endocrine disorders

- Possible symptoms: Fatigue, palpitations, weight changes, or temperature intolerance.
- Management:
  - Regular hormone and thyroid function monitoring;
  - Hormone replacement therapy may be needed.

(3) Immune-related inflammation, such as pneumonitis, colitis, or hepatitis

- Possible symptoms: Persistent cough, shortness of breath, severe diarrhea, abdominal pain, or jaundice.
- Management:
  - Contact the healthcare team promptly;
  - Immunotherapy may need to be paused, and corticosteroids or other treatments may be required.

5. When Should I Seek Immediate Medical Attention?

Contact your doctor or go to the hospital promptly if you experience:

- Fever (for example, temperature ≥38°C);
- Significant shortness of breath or chest pain;
- Inability to eat or drink adequately;
- Severe vomiting or diarrhea;
- Unexplained bleeding;
- Confusion or extreme weakness.

Summary:
Side effects are common during esophageal cancer treatment, but most can be effectively managed. Regular monitoring of blood counts and organ function, early reporting of symptoms, and close communication with the medical team can help maintain treatment effectiveness and quality of life.

Question27: How should I maintain my nutrition during treatment for esophageal cancer?

Answer:
Maintaining good nutrition during esophageal cancer treatment (including surgery, radiotherapy, chemotherapy, targeted therapy, or immunotherapy) is very important. Adequate nutrition helps the body tolerate treatment better, reduces complications, supports recovery, and improves quality of life. Because esophageal cancer can affect swallowing and food intake, dietary strategies should be adjusted according to individual symptoms.

1. Ensure adequate calories and protein intake

- Increase protein intake to help maintain muscle, promote wound healing, and support immune function. Good sources include:
  - Fish, shrimp, chicken, lean meat
  - Eggs
  - Milk and yogurt
  - Soy products (tofu, soy milk, etc.)
- If normal food intake is insufficient, oral nutritional supplements may be recommended under the guidance of a doctor or dietitian.

2. Eat small, frequent meals to improve swallowing

Patients with esophageal cancer may experience difficulty swallowing or discomfort while eating. Helpful strategies include:

- Eating 5–8 small meals per day instead of three large meals;
- Choosing soft, easy-to-swallow foods, such as:
  - Porridge
  - Noodles
  - Steamed eggs
  - Minced meat or fish
  - Vegetable puree
- Eating slowly and chewing thoroughly.

3. Adjust diet according to the treatment stage

During radiotherapy:

- Possible side effects include esophagitis, painful swallowing, and dry mouth;
- Choose soft, mild, and non-irritating foods;
- Avoid spicy, very hot, or hard foods.

During chemotherapy:

- If nausea or vomiting occurs:
  - Eat smaller meals more frequently;
  - Avoid greasy foods;
  - Maintain adequate fluid intake.
- If mouth sores develop:
  - Avoid acidic, spicy, or rough-textured foods.

After surgery:

- Resume eating gradually according to medical advice;
- Eat smaller portions to avoid overloading the stomach;
- Some patients may require specialized nutritional support.

4. Prevent weight loss and muscle loss

- Monitor body weight regularly;
- Inform your doctor if you lose significant weight (for example, more than 5% of body weight within one month);
- If your condition allows, maintain light physical activity such as walking or gentle resistance exercises to preserve muscle mass.

5. Maintain adequate hydration

- Drink enough fluids every day;
- If swallowing is difficult, take small sips frequently;
- Pay attention to fluid and electrolyte replacement if you have diarrhea, vomiting, or fever.

6. Avoid unhealthy dietary habits

- Avoid smoking and alcohol;
- Avoid heavily preserved, spoiled, or highly irritating foods;
- Do not take large amounts of supplements or unproven “anti-cancer foods” without medical advice, as some may interfere with treatment.

7. When should nutritional support be considered?

Contact your healthcare team or a dietitian if you experience:

- Difficulty eating or worsening swallowing problems;
- Continued weight loss;
- Severe fatigue or muscle loss;
- Persistent nausea or vomiting;
- Inability to meet nutritional needs through regular meals.

Summary:
The goal of nutrition during esophageal cancer treatment is to “eat enough, absorb enough, and maintain a stable weight.” A high-protein, nutrient-dense, small-and-frequent meal approach is usually recommended. Dietary adjustments should be made according to treatment-related side effects, and professional nutritional support should be considered when needed.

Question28: How should I gradually resume eating after esophageal cancer surgery?

Answer:
After esophageal cancer surgery, eating needs to be restarted gradually because the digestive tract anatomy has changed, the stomach may be used to reconstruct the esophagus, and swallowing function requires time to adapt. The recovery process should follow your surgeon’s and dietitian’s guidance.

1. Early postoperative period: fasting and nutritional support

- Shortly after surgery, patients are usually not allowed to eat by mouth to allow the surgical connection (anastomosis) between the esophagus and stomach or intestine to heal.
- Nutrition may be provided through intravenous nutrition or a feeding tube, such as a jejunostomy tube.
- Oral intake usually begins only after doctors confirm that healing is satisfactory and there are no major complications.

2. First oral intake: start with clear liquids

Once approved by the medical team, patients usually begin with small amounts of liquids, such as:

- Warm water
- Rice water
- Light broth
- Thin porridge
- Nutritional supplement drinks

Tips:

- Drink small amounts at a time.
- Eat slowly.
- Watch for swallowing difficulty, coughing, chest discomfort, or bloating.

3. Progress to soft and semi-liquid foods

If liquids are tolerated well, gradually introduce thicker foods, such as:

- Soft porridge
- Steamed egg custard
- Tofu
- Soft noodles
- Minced meat or fish

Recommendations:

- Choose foods that are soft and easy to digest.
- Chew thoroughly and eat slowly.
- Eat small, frequent meals (often 5–8 times per day).

4. Gradual return to a regular diet

Over weeks to months, many patients can gradually return to a more normal diet:

- Include high-quality protein sources, such as fish, eggs, lean meat, dairy products, and soy products.
- Add vegetables and fruits according to tolerance.
- Increase portion sizes gradually.

5. Important eating habits after surgery

- Eat small, frequent meals: The stomach may have reduced capacity after surgery, so large meals can cause discomfort.
- Chew food well: This helps food pass more easily through the surgical connection.
- Eat slowly: Reduces the risk of choking and swallowing problems.
- Avoid irritating foods: Limit spicy, very acidic, hard, or extremely hot foods.
- Avoid lying down immediately after meals: Stay upright for 30–60 minutes to reduce reflux.
- Limit excessive sugary foods: Some patients develop dumping syndrome, causing symptoms such as rapid heartbeat, sweating, dizziness, abdominal cramps, or diarrhea after eating.

6. When to contact your doctor

Seek medical advice if you experience:

- Persistent or worsening swallowing difficulty
- Repeated coughing, choking, or fever
- Severe chest or abdominal pain
- Persistent vomiting
- Significant weight loss or inability to maintain adequate nutrition

Summary:
Diet recovery after esophageal cancer surgery usually follows the pathway of fasting with nutritional support → liquids → semi-liquid foods → soft foods → gradual return to a regular diet. The recovery speed varies depending on the surgical method, location of the reconstruction, overall health, and presence of complications. A personalized plan from your surgical team and nutrition specialist is recommended.

Question29: How should the pain caused by esophageal cancer be managed?

Answer:
Pain caused by esophageal cancer should be managed through a comprehensive approach based on the cause, severity, and overall condition of the patient. Effective pain control can improve quality of life, sleep, nutritional intake, and tolerance to cancer treatment.

1. Assess the cause and severity of pain

Doctors usually evaluate:

- Location of pain: such as behind the breastbone, upper abdomen, back, or metastatic sites;
- Nature of pain: such as burning, stabbing, pressure-like, or persistent aching pain;
- Pain intensity: often measured using a 0–10 pain scale;
- Possible causes, including:
  - Tumor invasion of nearby tissues or nerves;
  - Lymph node or distant metastases;
  - Pain related to surgery, radiotherapy, or chemotherapy;
  - Esophageal narrowing, inflammation, or difficulty swallowing.

2. Pain-relieving medications

Treatment is usually adjusted according to pain severity:

- Mild pain:
  - Non-opioid pain relievers such as acetaminophen may be used;
  - Non-steroidal anti-inflammatory drugs (NSAIDs) may be considered in selected patients, with attention to risks such as bleeding, kidney problems, and stomach irritation.
- Moderate to severe pain:
  - Opioid medications such as morphine, oxycodone, or fentanyl may be prescribed;
  - Doctors adjust doses according to pain control and monitor side effects such as nausea and constipation.
- Neuropathic pain:
  - If pain is caused by nerve involvement from the tumor, medications such as gabapentin or pregabalin may be used.

3. Treating the cancer itself

Controlling tumor growth can also relieve pain, including:

- Chemotherapy, radiotherapy, targeted therapy, or immunotherapy;
- Local radiotherapy to reduce pain caused by bone metastases or tumor pressure;
- Esophageal stent placement to relieve obstruction and swallowing-related discomfort.

4. Non-medication approaches

Additional supportive methods may include:

- Adjusting eating habits and avoiding hard, very hot, or irritating foods;
- Maintaining appropriate physical activity;
- Heat therapy, relaxation techniques, and psychological support;
- Referral to a pain management or palliative care team when needed.

5. When should medical help be sought urgently?

Contact a doctor promptly if:

- Pain suddenly becomes much worse;
- Current pain medication no longer provides relief;
- Severe chest pain or difficulty breathing occurs;
- Swallowing becomes impossible or food and liquids cannot be tolerated;
- Persistent vomiting, black stools, or significant weight loss develops.

Summary:
Pain from esophageal cancer should be managed with a combination of identifying the cause, using appropriate pain medications, controlling the cancer, and providing supportive care. Patients should not tolerate uncontrolled pain and should report changes in pain promptly so that treatment can be adjusted.

Question30: How often should I have follow-up examinations after treatment for esophageal cancer, and what items are included?

Answer:
The frequency and content of follow-up after esophageal cancer treatment should be individualized based on factors such as the type of treatment received (surgery, chemoradiotherapy, immunotherapy, etc.), cancer stage, pathological findings, risk of recurrence, and overall health condition.

1. Follow-up schedule

- First 2 years after treatment:
  Follow-up is usually recommended every 3–6 months, as this period carries a relatively higher risk of recurrence.
- Years 3–5:
  Follow-up is generally recommended every 6–12 months.
- After 5 years:
  Long-term follow-up may continue depending on the patient’s condition, often annually or as clinically indicated.

Patients with higher recurrence risk (such as those with advanced disease, lymph node involvement, or other high-risk features) may require more frequent monitoring.

2. Common follow-up examinations

(1) Medical history review and physical examination
Doctors will ask about symptoms such as:

- Difficulty swallowing or painful swallowing
- Unintentional weight loss
- Chest or back pain
- Hoarseness
- Cough or shortness of breath
- Loss of appetite or fatigue

They will also assess nutritional status, weight changes, and recovery after treatment.

(2) Imaging examinations
Depending on the situation, doctors may recommend:

- Contrast-enhanced CT of the chest and abdomen: to evaluate local recurrence, lymph nodes, and distant metastases (such as lung or liver metastases).
- PET-CT: sometimes used when recurrence or metastasis is suspected.
- MRI or other imaging tests: when clinically appropriate.

(3) Endoscopic examination
Upper gastrointestinal endoscopy may be used to evaluate:

- The surgical anastomosis site after esophagectomy
- Recurrent or new lesions in the esophageal lining
- Narrowing, inflammation, or other local problems

Endoscopic surveillance is particularly important for patients treated with endoscopic resection or local therapies.

(4) Laboratory tests
Possible tests include:

- Complete blood count
- Liver and kidney function tests
- Nutritional markers (such as albumin)
- Tumor markers (such as CEA or SCC, depending on tumor type and physician judgment)

It is important to understand that no single blood test can reliably detect recurrence of esophageal cancer by itself. Results must be interpreted together with imaging findings and clinical symptoms.

(5) Assessment of function and quality of life
Doctors may also evaluate:

- Swallowing function
- Ability to maintain adequate nutrition
- Weight changes
- Postoperative problems such as reflux or dumping syndrome
- Psychological well-being and quality of life

3. Seek medical attention earlier if the following occur

Do not wait for the next scheduled follow-up if you develop:

- Progressive difficulty swallowing
- Significant unexplained weight loss
- Persistent chest or back pain
- Coughing blood or worsening hoarseness
- Persistent cough or breathing difficulty
- Vomiting blood or black stools

Early evaluation can help detect recurrence or treatment-related complications as soon as possible.

**Deepseek**

**Q1: What are the main risk factors for esophageal cancer?**

**A:** The main risk factors for esophageal cancer can be categorized into lifestyle factors, medical conditions, demographic factors, and others. It's important to note that the risk factor profiles differ significantly between the two main subtypes: **squamous cell carcinoma** and **adenocarcinoma**.

**1. Lifestyle and Habits**

- **Tobacco and Alcohol Use**: These are the most significant risk factors for esophageal squamous cell carcinoma. Any form of tobacco use and heavy alcohol consumption greatly increase the risk. The combination of both has a synergistic effect, further elevating the risk. One analysis found that daily alcohol consumption can increase the risk by **7.81 times**.
- **Dietary Factors**: Long-term consumption of very hot food or beverages (e.g., above **65°C/149°F**) can repeatedly injure the esophageal lining, increasing cancer risk. Studies show that people who regularly consume food above **60°C (140°F)** have a **2-fold** higher risk of esophageal cancer compared to those who consume food below 40°C (104°F). Other dietary risks include a low intake of fresh fruits and vegetables, a diet high in processed meats, and chewing betel quid.
- **Obesity and Physical Inactivity**: Obesity is a major risk factor for esophageal adenocarcinoma. Being overweight or obese increases the likelihood of gastroesophageal reflux, thereby raising cancer risk. A lack of physical exercise is also considered a risk factor.

**2. Medical Conditions**

- **Gastroesophageal Reflux Disease (GERD) and Barrett's Esophagus**: Chronic acid reflux (GERD) is a strong risk factor for esophageal adenocarcinoma. Persistent acid irritation can cause cellular changes in the lower esophagus, leading to Barrett's esophagus, a precancerous condition that significantly increases the risk of adenocarcinoma.
- **Other Esophageal Conditions**: Other esophageal disorders are linked to increased risk, including achalasia, esophageal stricture caused by ingesting corrosive substances (like lye), and esophageal webs (Plummer-Vinson syndrome).
- **Infection**: Human papillomavirus (HPV) infection is considered a risk factor.

**3. Demographic and Genetic Factors**

- **Age and Sex**: Increasing age is a risk factor, with esophageal cancer being more common in people over **50**. Men have a significantly higher risk of developing the disease than women.
- **Genetics and Family History**: Individuals with a family history of esophageal cancer or Barrett's esophagus have a **2 to 4 times** higher risk. Certain genetic syndromes (e.g., Bloom syndrome, Fanconi anemia) also increase the risk.

**4. Other Factors**

- Additional risk factors include previous radiation therapy to the esophagus for other cancers, poor oral hygiene, and exposure to certain chemical carcinogens (e.g., N-nitroso compounds).

**Q2: What are the main differences in causes between esophageal squamous cell carcinoma and adenocarcinoma?**

**A:** Esophageal squamous cell carcinoma (ESCC) and esophageal adenocarcinoma (EAC) are the two main histological types of esophageal cancer, and they differ significantly in their causes and risk factors. In general, ESCC is strongly linked to lifestyle and dietary habits, while EAC is primarily associated with obesity and gastroesophageal reflux.

**I. Main Causes of Esophageal Squamous Cell Carcinoma (ESCC)**

Esophageal squamous cell carcinoma originates from the squamous epithelial cells lining the esophagus. Its primary risk factors include:

1. **Tobacco and Alcohol Use**: These are the most important and well-established risk factors for ESCC. Studies show that smokers have a 9-fold higher risk of developing ESCC compared to non-smokers.
2. **Dietary Habits**: Long-term consumption of very hot beverages, pickled vegetables, and insufficient vitamin intake can cause repeated injury and repair of the esophageal mucosa, increasing the risk of cancer.
3. **Other Factors**: Human papillomavirus (HPV) infection is also considered a risk factor for ESCC.
4. **Geographic Distribution**: ESCC is more common in East Asia, the Middle East, and parts of Africa, accounting for over 90% of esophageal cancer cases in China.

**II. Main Causes of Esophageal Adenocarcinoma (EAC)**

Esophageal adenocarcinoma originates from metaplastic glandular epithelial cells in the lower esophagus that have changed due to chronic irritation. Its primary risk factors include:

1. **Gastroesophageal Reflux Disease (GERD) and Barrett's Esophagus**: Chronic reflux of stomach acid or bile damages the lower esophageal lining, causing the normal squamous epithelium to be replaced by columnar epithelium, a precancerous condition known as Barrett's esophagus. People with GERD have a 3.48-fold higher risk of developing EAC.
2. **Obesity**: Obesity is another major risk factor for EAC, particularly central/visceral obesity.
3. **Geographic Distribution**: EAC is more common in Western developed countries such as North America and Europe, though its incidence in China has been rising in recent years.

**III. Summary of Key Differences**

| Feature | Esophageal Squamous Cell Carcinoma (ESCC) | Esophageal Adenocarcinoma (EAC) |
| --- | --- | --- |
| **Cell of Origin** | Esophageal squamous epithelial cells | Metaplastic columnar glandular cells |
| **Main Risk Factors** | Smoking, alcohol, hot beverages, pickled foods | GERD, Barrett's esophagus, obesity |
| **Common Location** | Upper and middle esophagus | Lower esophagus, near the gastroesophageal junction |
| **Geographic Distribution** | More common in East Asia, Africa | More common in North America, Europe |

**Q3: What are the precancerous lesions of esophageal cancer?**

**A:**

Precancerous lesions of the esophagus are pathological changes that have the potential to develop into esophageal cancer. Based on the two main histological types of esophageal cancer — esophageal squamous cell carcinoma (ESCC) and esophageal adenocarcinoma — the precancerous lesions differ accordingly.

**I. Precancerous Lesions of Esophageal Squamous Cell Carcinoma**

The precancerous lesion of esophageal squamous cell carcinoma primarily refers to **squamous dysplasia** of the esophageal epithelium, which the World Health Organization terms **intraepithelial neoplasia**. It is characterized by abnormalities in cell morphology, size, and structure, including pleomorphic cells, hyperchromatic mitotic figures, immature cells with atypical mitosis, and loss of normal cell polarity.

Based on the degree of dysplasia and the depth of epithelial involvement, intraepithelial neoplasia is classified into two grades:

- **Low-grade intraepithelial neoplasia (LGIN)**: Dysplastic cells are confined to the lower half of the epithelium
- **High-grade intraepithelial neoplasia (HGIN)**: Dysplastic cells involve the upper half or more of the epithelium

**II. Precancerous Lesions of Esophageal Adenocarcinoma**

The most important precancerous lesion for esophageal adenocarcinoma is **Barrett's esophagus with associated dysplasia**. Barrett's esophagus is a pathological condition in which the normal stratified squamous epithelium of the lower esophagus is replaced by metaplastic columnar epithelium.

Barrett's esophagus is the major predisposing factor for esophageal adenocarcinoma, with a cancer risk 30 to 125 times that of the general population. The progression from Barrett's esophagus without dysplasia to low-grade dysplasia, then to high-grade dysplasia, and eventually to esophageal adenocarcinoma is well recognized.

**III. Other Related Precancerous Conditions**

In addition to the above precancerous lesions, there are several benign conditions associated with an increased risk of esophageal cancer, often referred to as **precancerous conditions**:

- Chronic esophagitis / Reflux esophagitis
- Achalasia
- Esophageal leukoplakia
- Esophageal diverticulum
- Esophageal polyp
- Esophageal ulcer
- Esophageal stricture
- Hiatal hernia
- Plummer-Vinson syndrome

**Q4: Is esophageal cancer hereditary?**

**A:** Esophageal cancer is not typically considered a directly inherited disease, but it does show familial aggregation and genetic susceptibility.

**1. Not Directly Inherited**

Current medical consensus holds that esophageal cancer is not a directly inherited disease and will not inevitably be passed on to the next generation like a single-gene disorder. Most gene mutations that lead to esophageal cancer are acquired after birth due to environmental factors such as smoking, alcohol consumption, and acid reflux, rather than being inherited.

**2. Familial Clustering Exists**

Esophageal cancer shows significant familial clustering. If a first-degree relative (parent, sibling, or child) has had esophageal cancer, the individual's risk of developing the disease is higher than that of the general population.

Key statistics:

- In high-incidence areas of China, **25%–50%** of esophageal cancer patients have a family history of the disease.
- A study involving 33,008 participants found that having a first-degree relative with esophageal cancer was associated with a familial risk ratio of **1.65**.
- If the relative was diagnosed **before age 35**, the risk ratio increased to **4.05**.
- Familial aggregation decreases with increasing age.

**3. Mechanisms of Genetic Susceptibility**

The familial clustering of esophageal cancer is attributed to "genetic susceptibility"—meaning that certain inherited genetic variants make individuals more sensitive to carcinogenic factors. The development of esophageal cancer involves changes in multiple genes, including cell cycle regulators, growth factor-related genes, apoptosis-related genes, and others.

In addition, certain rare genetic syndromes are associated with an elevated risk of esophageal cancer, such as tylosis (with a lifetime risk of over 90%), Bloom syndrome, Fanconi anemia, and familial Barrett's esophagus.

**4. Lifestyle Factors Also Play a Key Role**

Part of the familial clustering may also be due to shared lifestyle habits among family members (such as dietary patterns, smoking, and alcohol consumption), rather than being purely genetic.

**Recommendation:** If you have a first-degree relative with esophageal cancer—especially if they were diagnosed at a young age (e.g., under 40)—it is advisable to undergo regular endoscopic screening and maintain a healthy lifestyle, avoiding risk factors such as smoking, heavy alcohol consumption, and consumption of extremely hot foods and beverages.

**Q5: What is the peak age of onset for esophageal cancer?**

**A:** The peak age of onset for esophageal cancer varies by region, sex, and histological type, but it is **most commonly diagnosed in individuals over 50 years of age**.

- **General Age Range**: The risk of esophageal cancer increases significantly from age 40 onwards, with about **80% of patients diagnosed after age 50**. Most statistics indicate a high-incidence age range between **50 and 70 years**.
- **Peak Incidence**: Different studies provide varying peak age ranges. Some data show the highest incidence in the **60–64 years** age group. However, according to the latest Global Burden of Disease study, the peak age for **esophageal cancer deaths in China shifted to the 70–74 years** age group in 2021.
- **Differences by Histological Type**:
  - **Esophageal Squamous Cell Carcinoma (the most common type in China)**: Typically presents at an older age, mostly **after age 60**.
  - **Esophageal Adenocarcinoma (the main type in Western countries)**: The median age at diagnosis is around **68 years**.
- **Trends in Younger Populations**: Although the peak age is in the elderly, there has been a trend toward **younger onset** in recent years, with increasing incidence in the **45–55 years** age group. Furthermore, patients diagnosed with esophageal adenocarcinoma under the age of 50 often present with more advanced stages and have a poorer prognosis.

**Q6: What early symptoms of esophageal cancer should I be alert for?**

**A:** Esophageal cancer typically **causes no signs or symptoms** in its early stages. This is why it is often detected at a more advanced stage, when it becomes more challenging to treat. However, as the cancer begins to grow, some subtle warning signs may appear, including:

1. **Difficulty swallowing (dysphagia) or a feeling of food getting stuck**: This is one of the earliest and most significant warning signs. You may feel that food passes slowly or gets stuck briefly in your chest, especially when eating dry or solid foods like bread, meat, or crackers. The sensation may come and go, disappear on its own, but then return with increasing frequency and severity.
2. **Chest pain, pressure, or burning sensation**: You may experience mild pain, pressure, burning, or discomfort behind the breastbone (sternum) while eating. This pain is often mild and intermittent and can be easily mistaken for heartburn, acid reflux, stomach problems, or even heart-related issues.
3. **A feeling of a foreign body in the throat or chest**: A sensation that something is stuck in the esophagus, as if food is lodged and cannot be swallowed down or coughed up. This feeling may also occur when swallowing saliva.
4. **Dryness or tightness in the throat**: Some people experience a persistent dry or tight feeling in the throat or neck, along with difficulty swallowing and mild pain. This sensation does not go away with drinking water and may last longer than a typical sore throat.
5. **Other nonspecific symptoms**: Some early-stage patients may only have mild loss of appetite, abdominal bloating, or slight belching after meals. These vague symptoms are easily dismissed as minor indigestion or gastritis.

**Important Reminders:**

- Early symptoms are often **mild and intermittent**, making them easy to overlook.
- If you develop **progressive difficulty swallowing** — starting with solid foods, then soft foods, and eventually liquids — this usually indicates the cancer has reached a more advanced stage.
- People over age 40, especially those with risk factors such as long-term smoking, heavy alcohol use, preference for hot or pickled foods, family history of esophageal cancer, Barrett's esophagus, or chronic esophagitis, should pay close attention to these symptoms and seek medical evaluation — including endoscopy screening — promptly when concerned.

**Q7: What are the clinical manifestations of esophageal cancer?**

**A:** The clinical manifestations of esophageal cancer are closely related to its stage. Early-stage symptoms are often subtle and nonspecific, and most patients do not develop typical symptoms until the cancer has reached an advanced stage.

**I. Early Symptoms**

Early esophageal cancer often presents with no obvious symptoms, or only mild, intermittent symptoms. Common presentations include:

- **Sensation of food sticking or foreign body sensation**: When eating coarse or solid foods, patients may feel that food passes slowly, gets stuck temporarily, or experience a foreign body sensation behind the sternum. Symptoms may disappear spontaneously but tend to recur with increasing frequency and severity.
- **Substernal discomfort or pain**: Burning, stinging, or dragging pain behind the sternum during eating. When the lesion is in the lower esophagus, pain may be felt below the xiphoid process or in the upper abdomen, easily confused with gastritis.
- **Other nonspecific symptoms**: Loss of appetite, belching, acid reflux, and a feeling of throat dryness or tightness.

**II. Advanced-Stage Symptoms**

As the tumor grows and the esophageal lumen progressively narrows, symptoms become more typical and persistent.

- **Progressive dysphagia (most classic symptom)**: This is the core symptom of esophageal cancer and the main reason most patients seek medical attention. Initially, patients have difficulty swallowing solid foods (e.g., bread, rice) and need to chew carefully or drink water to help swallow. This progresses to difficulty swallowing soft foods (e.g., noodles, porridge), and eventually, even liquids (milk, soup) and saliva cannot be swallowed. The continuous progression of symptoms is an important feature distinguishing esophageal cancer from esophageal spasm, benign strictures, and other conditions.
- **Pain**: Persistent dull pain or burning sensation in the chest or back is a common symptom in advanced stages, aggravated by eating.
- **Regurgitation and vomiting**: Due to esophageal obstruction, food cannot pass into the stomach and is regurgitated. Vomitus typically consists of undigested food (without acid taste). Nocturnal regurgitation and choking may occur when lying flat, potentially leading to aspiration pneumonia.
- **Weight loss and systemic wasting**: Due to difficulty eating leading to malnutrition, patients gradually develop weight loss, dehydration, and weakness. Cachexia may occur in the terminal stage.
- **Hoarseness and choking**: Hoarseness may occur when the tumor invades the recurrent laryngeal nerve. If an esophagotracheal fistula forms, severe choking may occur during swallowing.
- **Metastasis-related symptoms**: These may include lymphadenopathy, liver metastasis (jaundice, ascites), bone metastasis (bone pain), and brain metastasis (coma).

It is important to note that symptoms of esophageal cancer can also be caused by other, more common conditions. If any of the above symptoms persist for more than one month without improvement, prompt medical evaluation is recommended.

**Q8: How can I tell whether my esophageal cancer symptoms are worsening?**

**Answer:**

**Determining whether your esophageal cancer symptoms are worsening involves closely monitoring the severity and frequency of your core symptoms. Symptoms typically intensify as the tumor grows. Below are key signs of deterioration to watch for.**

**I. Worsening of Core Symptoms**

1. **Progressive Dysphagia (Difficulty Swallowing - The Most Important Indicator): This is the most common and characteristic symptom of esophageal cancer. Signs of worsening include:**
   - **Expanding range: Progressing from difficulty with solid foods (e.g., bread, meat) to semi-solid foods (e.g., porridge, noodles).**
   - **Increasing severity: Eventually, even liquids (e.g., milk, soup) and saliva become difficult to swallow, possibly requiring frequent spitting of mucus. The progressive nature of this difficulty is a hallmark of malignancy.**
2. **Changes in Pain Nature and Intensity:**
   - **Increased pain: Pain behind the breastbone or in the back may change from mild discomfort during eating to persistent dull pain or a burning sensation.**
   - **Pain association: Pain is notably worse during eating, especially with solid foods.**
3. **Unexplained Weight Loss: Approximately 50% of esophageal cancer patients experience this. Continuing to lose weight even without a significant decrease in food intake is a critical sign of progression.**
4. **Emergence of New or Accompanying Symptoms:**
   - **Regurgitation and Vomiting: Vomiting undigested food shortly after eating due to esophageal obstruction.**
   - **Hoarseness or Persistent Cough: The tumor may compress the recurrent laryngeal nerve, causing a hoarse voice that does not improve; or a cough lasting more than two weeks.**
   - **Vomiting Blood or Black Stools: This may indicate bleeding from the tumor.**

**II. When to Seek Immediate Medical Attention**

**You must contact your doctor or go to the hospital immediately if you experience any of the following, as they suggest rapid deterioration:**

- **Significant worsening of dysphagia, e.g., progressing from soft foods to being unable to swallow liquids.**
- **Pain becomes severe and persistent, unresponsive to usual measures.**
- **Development of alarming new symptoms, such as vomiting blood, black stools, severe cough, or fever (which may indicate esophageal perforation or infection).**
- **Rapid and significant weight loss in a short period.**

**Please remember: Early-stage esophageal cancer often causes no symptoms. The appearance of any of the above symptoms, especially progressive worsening after a diagnosis, is a serious warning sign from your body and requires immediate professional medical attention.**

**Q9: Which organs does esophageal cancer most commonly spread to, and what symptoms occur after metastasis?**

**A:** The most common sites of distant metastasis for esophageal cancer are the **liver, lungs, bones, and lymph nodes**.

Metastasis to different organs causes specific symptoms:

- **Liver Metastasis**:
  - Discomfort or pain in the upper right abdomen
  - Abdominal bloating or swelling (ascites)
  - Yellowing of the skin and eyes (jaundice)
  - Nausea and loss of appetite
  - Fever
- **Lung Metastasis**:
  - Persistent cough
  - Shortness of breath
  - Coughing up blood
  - Chest pain
  - Recurrent chest infections
- **Bone Metastasis**:
  - Progressive local bone pain, often described as a continuous gnawing pain
  - Pathological fractures (bones become brittle and break more easily)
  - Backache that worsens despite resting
  - Raised blood calcium, which can cause dehydration, confusion, nausea, and constipation
- **Lymph Node Metastasis**:
  - Hard or swollen lymph nodes, most commonly in the neck
  - Worsening difficulty swallowing if swollen neck lymph nodes press on the esophagus
- **Brain Metastasis**:
  - Headache, confusion, and seizures
  - Dizziness and balance problems
- **Other Sites**:
  - **Peritoneal metastasis** can cause ascites, abdominal pain, and bowel obstruction.
  - **Kidney metastasis** is often asymptomatic.

**Q10: What emergency situations in patients with esophageal cancer require immediate visit to the emergency department?**

**A:** Patients with esophageal cancer may experience life-threatening acute complications during the course of their disease or after treatment, requiring immediate emergency department evaluation. The main emergency indications include:

**1. Severe Dysphagia, Inability to Maintain Hydration and Nutrition**
When the patient cannot swallow even liquids or saliva, it suggests severe esophageal obstruction requiring urgent assessment and management.

**2. Significant Bleeding**
Manifestations include hematemesis (vomiting blood or coffee-ground material), melena (tarry black stools), or severe anemia. Bleeding may result from tumor invasion of blood vessels or vascular rupture following radiotherapy or chemotherapy. Massive bleeding can be life-threatening.

**3. Esophageal Perforation**
Characterized by sudden, severe pain in the neck or chest, accompanied by dysphagia. Esophageal perforation can lead to severe thoracic or abdominal infection and is life-threatening, requiring emergency treatment.

**4. Severe Dyspnea or Airway Obstruction**
When the tumor invades or compresses the trachea or main bronchi, it can cause acute respiratory distress, wheezing, and inspiratory "three-depression sign" (suprasternal, supraclavicular, and intercostal retractions). This is a life-threatening emergency with a high mortality rate and requires immediate medical attention.

**5. Uncontrolled Severe Pain**
Includes severe chest pain, back pain, etc., suggesting possible tumor invasion of nerves, compression of vital structures, or perforation.

**6. Febrile Neutropenia**
A common serious complication after chemotherapy in cancer patients, carrying a high risk of infection due to immunocompromise, requiring emergency management.

**7. Superior Vena Cava Obstruction**
Manifested as facial and neck swelling, dyspnea, and engorged chest wall veins, indicating tumor compression of the superior vena cava — an oncological emergency.

**8. Symptomatic Malignant Hypercalcemia**
Tumor-induced hypercalcemia can cause confusion, nausea, vomiting, and arrhythmias, requiring urgent management.

**9. Postoperative Emergencies**
After esophagectomy, complications such as hemothorax (bleeding into the chest cavity), chylothorax (lymphatic fluid accumulation in the chest), tracheobronchial injury (fistula), pneumothorax, and pyothorax may occur, all requiring emergency management.

**10. Other Conditions Requiring Urgent Evaluation**

- Suspected esophageal or gastric foreign body impaction
- Severe electrolyte disturbances (e.g., hypokalemia, hyponatremia)
- Inability to maintain hydration and nutrition

**Reminder:** If any of the above conditions occur, call emergency services immediately or have the patient transported to the nearest emergency department. If the patient experiences choking due to food obstruction during transport, the Heimlich maneuver may be attempted, but avoid excessive force; if ineffective, call for emergency help immediately.

**Q11: Which department should I visit after being diagnosed with esophageal cancer?**

**A:**
After an esophageal cancer diagnosis, you typically **do not see just one specialty**—you need a **multidisciplinary team (MDT)** to develop a personalized treatment plan. Based on your disease stage, the following core departments are involved:

1. **Thoracic Surgery** – If surgical resection is feasible (early or locally advanced stages), thoracic surgeons perform esophagectomy and related procedures.
2. **Medical Oncology** – Responsible for systemic therapies, including neoadjuvant chemo/immunotherapy, adjuvant chemotherapy, and targeted/immunotherapy or palliative chemotherapy for metastatic disease.
3. **Radiation Oncology** – If radiotherapy is indicated (e.g., locally advanced unresectable disease or preoperative chemoradiation), radiation oncologists design and deliver the radiation plan.
4. **Gastroenterology** – Handles endoscopic diagnosis, staging (EUS), nutritional support, and palliative endoscopic interventions (stent placement, dilation, etc.).
5. **Clinical Nutrition** – Esophageal cancer often causes dysphagia and malnutrition; dietitians are essential for full-course nutritional support.

**Practical advice:**
Your first stop should be a **major tertiary hospital's cancer center or specialized esophageal cancer clinic** that offers a fixed MDT conference. You may start with either **Thoracic Surgery** or **Medical Oncology** as the entry point, and your primary doctor will coordinate the MDT for you. Bring all your diagnostic materials (gastroscopy reports, pathology slides, CT/PET-CT, EUS, etc.) to the visit.

**Q12: What is the gold standard for diagnosing esophageal cancer?**

**A:**

The "gold standard" for diagnosing esophageal cancer is **upper gastrointestinal endoscopy (esophagogastroduodenoscopy, EGD) combined with pathological biopsy**.

Specifically, this standard consists of two key steps:

1. **Endoscopic Visualization**: A flexible tube with a high-definition camera (endoscope) is inserted through the mouth into the esophagus, allowing direct visualization of the esophageal mucosa's morphology, color, and texture. White-light endoscopy is the fundamental method for examining esophageal mucosal diseases. When combined with advanced techniques such as narrow-band imaging (NBI) or chromoendoscopy (e.g., Lugol's iodine staining), the detection rate for early lesions can be further improved.
2. **Pathological Biopsy**: Tissue samples are obtained from suspicious lesions during endoscopy and sent to the pathology laboratory for microscopic examination. **Only the pathological biopsy result can definitively determine the nature of the lesion** (whether it is inflammation, a benign tumor, or cancer) and the specific type of cancer.

**Additional Notes:**

- Endoscopy is an **essential step** for diagnosis because the esophagus is located deep within the chest, and other imaging modalities cannot visualize mucosal details as directly.
- For patients who cannot undergo endoscopic biopsy, biopsy of other metastatic sites may be used to aid in diagnosis.
- It is important to distinguish that the **gold standard for diagnosis (confirmation)** is endoscopy with biopsy, while **staging** (assessing the depth of tumor invasion and extent of metastasis) relies on imaging modalities such as endoscopic ultrasound (EUS), CT, and PET-CT.

**Q13: After being diagnosed with esophageal cancer, what additional tests are needed to determine the severity of the disease?**

**A:**

After an esophageal cancer diagnosis, a series of tests are typically performed to determine the cancer's stage (i.e., its severity and extent). The stage is critical for planning the most effective treatment strategy. These staging tests generally fall into the following categories:

**I. Imaging Tests**

These are the cornerstone of staging, used to look for signs of cancer in lymph nodes or other parts of the body.

- **Contrast-Enhanced CT Scan**: Often the initial staging test, typically covering the neck, chest, and abdomen to evaluate for metastatic disease.
- **PET-CT Scan**: Combines PET and CT for more precise detection of tumor location and distant (stage IV) metastasis.
- **Magnetic Resonance Imaging (MRI)**: Used in certain situations. Some studies suggest that a combination of thoraco-abdominal MRI and PET/CT is highly accurate for initial staging and less invasive.

**II. Endoscopic and Ultrasound Procedures**

- **Endoscopic Ultrasound (EUS)**: An endoscope with an ultrasound probe. It accurately assesses the depth of tumor invasion into the esophageal wall (T stage) and the involvement of nearby lymph nodes (N stage), playing a central role in initial staging.
- **Bronchoscopy**: May be used if the tumor is in the upper esophagus to check if it has invaded the nearby trachea or bronchi.
- **Thoracoscopy, Laparoscopy, or Mediastinoscopy**: In some cases, these minimally invasive surgical procedures allow direct visualization and biopsy of lymph nodes or tissues for precise staging.

**III. Laboratory Tests (Blood Tests)**

- **Routine Blood Tests**: Include complete blood count (CBC), renal and liver function tests to assess overall health and check for conditions like anemia.
- **Tumor Marker Tests**: Detect levels of certain substances in the blood, which can serve as reference.
- **Molecular Pathology Tests**: For specific types of esophageal cancer, tests for PD-L1 expression or NTRK gene fusions may be performed to guide potential targeted or immunotherapy.

**IV. Other Tests**

- **Upper GI Series (Barium Swallow)**: Involves drinking a barium solution followed by X-rays to visualize the esophagus's shape, helping to detect narrowing or filling defects caused by the tumor.
- **Complete Physical Examination and History**: A thorough physical exam and detailed assessment of the patient's overall health and surgical risk are essential.

**Please Note**: Not every test is appropriate for every person. It is crucial to discuss with your doctor which specific tests are needed based on your individual situation.

**Q14: Question: What stage is my esophageal cancer?**

**Answer:**

**I understand your concern. It's important to know that only your medical team can determine your exact cancer stage after a thorough evaluation.**

**However, I can explain how doctors generally determine the stage of esophageal cancer, which can help you understand the process and communicate with your doctors.**

**Doctors use the TNM staging system to describe the extent of cancer. It evaluates three key factors:**

1. **T (Tumor): Describes how deeply the tumor has grown into the esophagus wall. It ranges from T1 to T4:**
   - **T1: Cancer has grown into the submucosa.**
   - **T2: Cancer has grown into the muscle layer.**
   - **T3: Cancer has grown into the outer covering of the esophagus.**
   - **T4: Cancer has grown into nearby structures like the trachea or aorta.**
2. **N (Node): Describes whether cancer has spread to nearby lymph nodes. It ranges from N0 to N3:**
   - **N0: No cancer cells in nearby lymph nodes.**
   - **N1: Cancer cells found in 1-2 nearby lymph nodes.**
   - **N2: Cancer cells found in 3-6 nearby lymph nodes.**
   - **N3: Cancer cells found in 7 or more nearby lymph nodes.**
3. **M (Metastasis): Describes whether cancer has spread to distant parts of the body.**
   - **M0: No distant metastasis.**
   - **M1: Distant metastasis is present.**

**The T, N, and M categories are combined to determine the overall stage, often expressed as Roman numerals 0 to IV. A higher number indicates a more advanced cancer. For example, Stage IV means the cancer has spread to other parts of the body.**

**Doctors use several tests to gather this staging information:**

- **Imaging tests: Such as CT, PET-CT, and MRI scans, to see the tumor's size, location, and if it has spread.**
- **Endoscopic Ultrasound (EUS): A key tool for assessing tumor depth (T stage) and regional lymph nodes (N stage).**
- **Biopsy: Taking a tissue sample during an endoscopy for pathological analysis, which is the gold standard for diagnosis.**

**Your final stage will be determined by your healthcare team after reviewing all your test results.**

**Q15: What do the HER2, PD-L1, dMMR/MSI, and Claudin18.2 indicators mean in an esophageal cancer pathology report?**

**A:**

In an esophageal cancer pathology report, **HER2, PD-L1, dMMR/MSI, and Claudin18.2 are four key biomarkers** that determine whether a patient is eligible for specific targeted therapies or immunotherapies. These test results directly guide the selection of precision treatment plans. Current guidelines recommend routine testing for all four biomarkers in gastroesophageal adenocarcinoma; for esophageal squamous cell carcinoma (ESCC), testing for PD-L1 and dMMR/MSI status is primarily recommended.

Here is what each indicator means:

**1. HER2 (Human Epidermal Growth Factor Receptor 2)**

- **What it is**: HER2 is an oncogene that promotes tumor cell proliferation. In some esophageal cancers (mainly adenocarcinomas), HER2 protein is overexpressed or the gene is amplified.
- **Clinical significance**: **HER2 positivity is the basis for using the targeted drug "Trastuzumab"**. Approximately **20%-30%** of esophageal adenocarcinoma patients are HER2-positive.
- **Testing method**: Usually, **Immunohistochemistry (IHC)** is used first to detect protein expression levels, and positive results are then confirmed for gene amplification using **In Situ Hybridization (ISH)**.

**2. PD-L1 (Programmed Death-Ligand 1)**

- **What it is**: PD-L1 is a protein on the surface of tumor cells that binds to the PD-1 receptor on immune T cells, effectively "turning off" T cell activity and helping cancer cells evade immune system attack.
- **Clinical significance**: **PD-L1 expression level is the most important predictor of response to immunotherapy (PD-1/PD-L1 inhibitors)**. Generally, higher PD-L1 expression (e.g., CPS≥1) is associated with greater likelihood of benefit from immunotherapy.
- **Testing method**: Detected via **Immunohistochemistry (IHC)** on tumor tissue, with results typically reported as **Combined Positive Score (CPS)** or **Tumor Proportion Score (TPS)**.

**3. dMMR/MSI (Deficient Mismatch Repair / Microsatellite Instability)**

- **What it is**: dMMR means the cell's DNA "mismatch repair" system is faulty and cannot properly fix errors that occur during DNA replication. This leads to an accumulation of gene mutations, manifesting as **Microsatellite Instability (MSI)**. dMMR and MSI-H (high instability) generally refer to the same condition.
- **Clinical significance**: **dMMR/MSI-H is another important predictor of response to immunotherapy**. Because these tumors accumulate many genetic errors, they produce many "foreign" proteins, making them更容易被免疫系统识别和攻击. Consequently, patients with dMMR/MSI-H typically respond very well to immune checkpoint inhibitors.
- **Testing method**: Usually detected by **Immunohistochemistry (IHC)** for the presence/absence of MMR proteins (MLH1, PMS2, MSH2, MSH6), or by **Polymerase Chain Reaction (PCR)/Next-Generation Sequencing (NGS)** to assess MSI status.

**4. Claudin18.2 (Claudin 18 Isoform 2)**

- **What it is**: Claudin18.2 is a protein normally found only in the tight junctions of gastric mucosal cells. When cells become cancerous, this protein becomes exposed on the cell surface, creating a targetable site for drugs.
- **Clinical significance**: **Claudin18.2 positivity is the basis for using targeted drugs (e.g., Zolbetuximab)**. Approximately **38%** of HER2-negative advanced gastric/gastroesophageal junction cancers are Claudin18.2-positive. It has limited overlap with other markers like HER2 and PD-L1, providing a new treatment opportunity for HER2-negative patients.
- **Testing method**: Detected via **Immunohistochemistry (IHC)** for Claudin18.2 protein expression on the surface of tumor cells.

**Q16: Is endoscopic resection suitable for early-stage esophageal cancer?**

**A:**

Yes, endoscopic resection (ER) is a suitable and increasingly standard treatment for selected patients with early-stage esophageal cancer, particularly for those with very superficial tumors and no evidence of lymph node metastasis. It is a minimally invasive alternative to traditional esophagectomy (surgical removal of the esophagus).

However, its suitability depends critically on the tumor's depth of invasion and other risk factors. The key is accurate pre-operative staging to determine if the cancer is confined to the layers where the risk of lymph node metastasis is acceptably low.

**Indications for Endoscopic Resection**

The primary criteria for considering ER are based on the depth of tumor invasion into the esophageal wall:

- **Absolute Indications (Standard of Care):**
  - **High-grade intraepithelial neoplasia (dysplasia)**
  - **M1 cancer:** Tumor limited to the epithelial layer
  - **M2 cancer:** Tumor invading the lamina propria
    For these stages, the risk of lymph node metastasis is minimal, making ER a curative-intent treatment.
- **Relative/Expanded Indications (Carefully Selected Cases):**
  - **M3 cancer:** Tumor invading the muscularis mucosa
  - **SM1 cancer:** Tumor invading the superficial third of the submucosa
    These lesions carry a 2%-8% risk of lymph node metastasis. ER may be considered if there is no clinical evidence of lymph node metastasis, but patients must be counseled on the higher risk and require meticulous post-procedure surveillance.

In clinical staging terms, ER is typically recommended for **cTis to cT1aN0M0** esophageal cancer. According to the 2026 CSCO guidelines, for Tis and T1a stages, a comprehensive assessment is required before ER, considering the lesion's size, circumferential extent, degree of differentiation, and presence of lymphovascular invasion.

**Absolute Contraindications**

ER is not suitable if:

- There is **definitive evidence of lymph node metastasis**.
- The tumor invades the **deep submucosa (SM2 or SM3)**, as these cases have a high risk of nodal metastasis and are generally recommended for surgical resection.

**Advantages of Endoscopic Resection**

When compared to esophagectomy, ER offers significant benefits:

- **Minimally Invasive:** Less surgical trauma.
- **Lower Complication Rate:** Reduced risk of perioperative complications.
- **Organ Preservation:** The esophagus is preserved, leading to better quality of life.
- **Shorter Hospital Stay and Faster Recovery**.
- **Comparable Long-term Survival:** For appropriately selected patients (e.g., cT1N0M0 squamous cell carcinoma), studies show that ER is associated with 5-year survival rates **comparable to those following esophagectomy**. One study reported a 5-year survival rate of 53% for ER versus 59% for surgery (p=0.77).

**Risks and Limitations**

While safer than surgery, ER is not without risks, including bleeding, perforation, and esophageal stricture (narrowing). The risk of stricture is particularly high (**88%-100%**) if the resection involves more than 3/4 of the esophageal circumference.

Furthermore, it is crucial to understand that ER is a **local treatment**; it does not remove regional lymph nodes. Therefore, it is only suitable for cancers with a very low probability of having spread to the lymph system.

**Conclusion**

In summary, **endoscopic resection is highly suitable and often the preferred treatment for early-stage esophageal cancer that is strictly confined to the mucosa (Tis, T1a) and has no signs of lymph node spread (N0)**. Its success depends on accurate pre-procedural staging and careful patient selection. For lesions that have invaded deeper into the submucosa (T1b), the decision is more complex and requires a multidisciplinary evaluation to weigh the risks and benefits.

**Q17: What are the surgical approaches for esophageal cancer?**

**A:** Surgical treatment for esophageal cancer primarily involves **esophagectomy**, which aims to completely remove the tumor and affected tissues, dissect regional lymph nodes, and reconstruct the digestive tract. The main approaches can be classified as follows:

**I. By Surgical Technique**

1. **Open Surgery**
   - The traditional approach, requiring large incisions in the chest and/or abdomen.
   - Associated with significant trauma and slower recovery; increasingly replaced by minimally invasive techniques.
2. **Minimally Invasive Esophagectomy (MIE)**
   - Performed through several small incisions using thoracoscopy and laparoscopy.
   - Offers less postoperative pain, fewer pulmonary complications (e.g., infections), and faster recovery compared to open surgery.
   - Has become the mainstream clinical trend.
3. **Robot-Assisted Minimally Invasive Esophagectomy (RAMIE)**
   - Utilizes a robotic surgical system in addition to minimally invasive techniques.
   - Provides 3D high-definition visualization and wristed instruments for greater precision within the confined thoracic cavity.
   - May further reduce operative time and blood loss.

**II. By Surgical Approach**

The choice of approach depends mainly on the tumor location.

| Approach | Indications | Characteristics |
| --- | --- | --- |
| **Left Thoracic Approach** | Middle/lower esophageal cancer | Relatively simple, less traumatic |
| **Right Thoracic + Upper Abdominal Approach (Ivor-Lewis)** | Middle/lower esophageal cancer, gastroesophageal junction cancer | Excellent exposure, enables thorough lymph node dissection |
| **Cervical-Thoracic-Abdominal Tri-Incision (McKeown)** | Upper/mid-upper esophageal cancer | Most extensive lymphadenectomy, but most invasive |
| **Transhiatal Approach** | Middle/lower esophageal cancer | Requires only neck and abdominal incisions; chest is not opened |
| **Thoracoabdominal Approach** | Lower esophageal or gastroesophageal junction cancer | Incision extends from abdomen to the left chest |

**III. By Digestive Tract Reconstruction**

After esophageal resection, other tissues are used to reconstruct the digestive tract.

- **Gastric Conduit (Gastric Pull-up)** : The most common method; the stomach is tubularized and pulled up to anastomose with the remaining esophagus.
- **Colon or Jejunal Interposition**: Used when the stomach is unavailable (e.g., after prior gastrectomy), the colon or jejunum can serve as an替代.

**IV. Special Consideration for Early-Stage Cancer**

For very early-stage esophageal cancer (e.g., T1a) with low risk of lymph node metastasis, **endoscopic resection (ER)** — including endoscopic mucosal resection (EMR) and endoscopic submucosal dissection (ESD) — may be considered. This approach is less invasive and can preserve the esophagus.

**Q18: Why is chemoradiotherapy needed before surgery for esophageal cancer?**

**A:**

Preoperative chemoradiotherapy for esophageal cancer is known as **neoadjuvant therapy** or **neoadjuvant chemoradiation**. For patients with locally advanced (stage II-III) resectable esophageal cancer, neoadjuvant chemoradiation followed by surgery has become the standard of care. The primary purposes and benefits include the following:

**1. Downstaging the Tumor to Enable More Complete Resection**

Neoadjuvant therapy can effectively shrink the primary tumor and clear involved local lymph nodes. Studies have shown that preoperative chemoradiation can downstage 43% of patients in T stage (depth of tumor invasion) and 46% in overall TNM stage. Tumor shrinkage can separate the tumor from surrounding vital organs (such as the trachea or aorta), converting a technically difficult or unresectable tumor into one that can be radically removed.

**2. Increasing the Rate of Successful R0 Resection**

By downstaging the tumor, surgeons can more easily achieve a complete resection with clear margins (R0 resection). Achieving an R0 resection is a critical factor for improving patient prognosis.

**3. Eliminating Micrometastases**

Esophageal cancer can spread systemically at an early stage through micrometastases, which are a major cause of recurrence after surgery. Preoperative chemotherapy can reach these microscopic cancer cells throughout the body via the bloodstream, eradicating them before they can grow.

**4. Leveraging the Synergistic Effect of Combined Chemoradiotherapy**

The combination of radiotherapy and chemotherapy has a synergistic effect. Radiotherapy enhances local tumor control, while chemotherapy not only targets systemic cancer cells but also acts as a "radiosensitizer," making tumor cells more sensitive to radiation and thus enhancing its effect.

**5. Improving Pathologic Complete Response (pCR) Rates and Long-Term Survival**

One of the goals of neoadjuvant therapy is to achieve a pathologic complete response (pCR), meaning no viable cancer cells are found in the resected specimen. Current studies indicate that pCR rates for neoadjuvant chemoradiation range from 20% to 35%. Patients who achieve pCR generally have better long-term survival outcomes. Numerous studies have confirmed that this multimodal approach significantly prolongs survival for patients with locally advanced esophageal cancer compared to surgery alone.

**In summary,** the purpose of preoperative chemoradiotherapy can be divided into two categories:

- **For primarily resectable tumors**: It serves as a "neoadjuvant" therapy to reduce the risk of postoperative recurrence and improve long-term outcomes through the mechanisms described above.
- **For locally advanced, difficult-to-resect tumors**: It serves as a "conversion" therapy, aiming to convert a technically unresectable tumor into a resectable one, thereby offering patients the chance for curative surgery.

**Q19: What are the most commonly used chemotherapy, immunotherapy, and targeted therapy drugs for advanced esophageal cancer?**

**A:**

The treatment of advanced esophageal cancer has evolved into a comprehensive approach based on chemotherapy, combined or sequenced with immunotherapy and targeted therapy. Drug selection varies according to histological type (squamous cell carcinoma vs. adenocarcinoma) and biomarkers (such as PD-L1, HER2, Claudin 18.2, etc.).

**I. Chemotherapy Drugs**

Chemotherapy remains the cornerstone of treatment for advanced esophageal cancer, often administered as two-drug or three-drug combination regimens.

**Commonly used chemotherapeutic agents include:**

- **Platinum agents**: Cisplatin, Oxaliplatin
- **Fluoropyrimidines**: 5-Fluorouracil (5-FU), Capecitabine
- **Taxanes**: Paclitaxel, Nab-paclitaxel, Docetaxel

**Common chemotherapy regimens:**

- **FP regimen**: Cisplatin + 5-Fluorouracil
- **TP regimen**: Paclitaxel + Cisplatin
- **DCF regimen**: Docetaxel + Cisplatin + 5-Fluorouracil

**II. Immunotherapy Drugs**

Immune checkpoint inhibitors (ICIs), particularly PD-1/PD-L1 inhibitors, have reshaped the treatment landscape for advanced esophageal cancer. Immunotherapy combined with chemotherapy is now the standard first-line treatment.

**Commonly used PD-1/PD-L1 inhibitors:**

- **Imported drugs**: Pembrolizumab (Keytruda), Nivolumab (Opdivo)
- **Domestic drugs (China)**: Toripalimab, Sintilimab, Camrelizumab, Tislelizumab, Serplulimab, Sugemalimab

**Common first-line immunotherapy combination regimens:**

- Nivolumab + FP (Fluorouracil + Cisplatin)
- Pembrolizumab + FP
- Toripalimab + TP (Paclitaxel + Cisplatin)
- Sintilimab + TP/FP
- Camrelizumab + TP
- Tislelizumab + TP/FP

**Dual immunotherapy regimens:**

- Nivolumab + Ipilimumab (CTLA-4 inhibitor)
- Cadonilimab (PD-1/CTLA-4 bispecific antibody)

**III. Targeted Therapy Drugs**

Targeted therapy is selected based on specific biomarkers. Currently, the targeted drugs approved globally for advanced esophageal cancer mainly include the following categories:

**1. Anti-HER2 targeted drugs (for HER2-positive esophageal adenocarcinoma/gastroesophageal junction adenocarcinoma)**

- **Trastuzumab**: First-line combination with chemotherapy
- **Trastuzumab Deruxtecan (T-DXd)**: Second-line and subsequent therapy

**2. Anti-angiogenic targeted drugs (multi-target TKIs)**

- **Apatinib**: Third-line and beyond
- **Anlotinib**: Multiple lines of therapy
- **Ramucirumab**: Targets VEGFR2, for previously treated patients

**3. Drugs targeting NTRK gene fusions (for NTRK fusion-positive patients)**

- Larotrectinib, Entrectinib

**4. Drugs targeting Claudin 18.2 (for Claudin 18.2-positive esophageal adenocarcinoma)**

- Zolbetuximab

**5. Novel Antibody-Drug Conjugates (ADCs)**

- **EGFR×HER3 bispecific ADC (Iza-bren/Lunkan Yilongtuomab)**: For recurrent/metastatic esophageal squamous cell carcinoma that has progressed after prior PD-1/PD-L1 inhibitor combined with platinum-based chemotherapy

**Note**: The above information is for reference only. Specific treatment plans should be formulated by professional physicians based on the patient's individual condition, histological type, biomarker status, and overall health status.

**Q20: What treatment options are available for esophageal cancer from early to advanced stages?**

**A:**

Treatment for esophageal cancer is highly dependent on the stage at diagnosis (i.e., the extent of tumor invasion and spread), as well as the tumor's histology (squamous cell carcinoma or adenocarcinoma) and the patient's overall health. Modern oncology has entered an era of "comprehensive treatment," typically requiring a multidisciplinary team (MDT) to devise an individualized plan. The following is a stage-by-stage breakdown of the main treatment strategies:

**1. Very Early Stage (Stage 0)**

- **Endoscopic Therapy**: For very early-stage lesions confined to the superficial layers of the esophageal mucosa (e.g., high-grade dysplasia), **Endoscopic Mucosal Resection (EMR)** or **Endoscopic Submucosal Dissection (ESD)** can be performed to remove the lesion. **Radiofrequency Ablation (RFA)** or **cryotherapy** may also be used to destroy precancerous tissue. These methods are minimally invasive and help preserve the esophagus.

**2. Early Stage (Stage I)**

- **Endoscopic Therapy**: Some eligible patients with early-stage cancer may still qualify for endoscopic resection.
- **Surgery**: **Esophagectomy** is the preferred curative treatment for early-stage disease. For early-stage patients, the 5-year survival rate after surgery can exceed 70%. The surgery can be performed using minimally invasive (thoracoscopic/laparoscopic) or robot-assisted techniques.
- **Chemoradiation**: For patients unfit for surgery, **definitive chemoradiation** may be considered as a curative option.

**3. Locally Advanced Stage (Stage II-III)**

- **Neoadjuvant Therapy + Surgery**: This is the standard of care for patients with resectable locally advanced cancer. **Neoadjuvant chemoradiation** or **neoadjuvant chemotherapy** is given before surgery. The goal is to shrink the tumor, increase the chances of complete surgical removal, and eliminate any microscopic cancer cells that may have spread. Depending on the situation, **adjuvant therapy** (chemotherapy, radiation, or immunotherapy) may be given after surgery to reduce the risk of recurrence.
- **Definitive Chemoradiation**: For patients with unresectable tumors or those who cannot tolerate surgery, **concurrent chemoradiation** is the traditional standard treatment.

**4. Locally Advanced Unresectable & Metastatic Stage (Stage IV)**

- **Systemic Therapy**: This is the cornerstone of treatment for advanced esophageal cancer, with the primary goals of controlling tumor growth and prolonging survival.
  - **Chemotherapy**: Remains a fundamental approach, with common regimens including paclitaxel combined with cisplatin.
  - **Immunotherapy**: **PD-1/PD-L1 inhibitors (e.g., Pembrolizumab, Nivolumab)** represent a major breakthrough in recent years. For previously untreated, advanced or metastatic patients, **immunotherapy combined with chemotherapy** has become one of the standard first-line options.
  - **Targeted Therapy**: For patients with specific genetic mutations (e.g., HER-2 positive), targeted drugs like **Trastuzumab** can be used.
- **Palliative Therapy**: Aimed at relieving symptoms and improving quality of life.
  - **Radiation Therapy**: Can be used to alleviate local symptoms such as bone metastasis pain or brain metastases.
  - **Endoscopic Intervention**: For patients with severe dysphagia, an **esophageal stent** can be placed to keep the esophagus open, or **laser therapy** can be used to burn away the tumor and open the passage.
  - **Nutritional Support**: Ensuring adequate nutritional intake via nasogastric tubes or gastrostomy.

**Q21: On what basis does the doctor formulate my treatment plan for esophageal cancer?**

**A:**

The formulation of an esophageal cancer treatment plan is a rigorous and personalized process. Doctors base their decisions on several core factors, following authoritative clinical guidelines such as those from NCCN and CSCO:

**1. Tumor-Related Factors (Most Critical)**

- **Histological Type**: Determining whether it is esophageal squamous cell carcinoma or adenocarcinoma, as treatment strategies differ.
- **Stage (TNM Staging)**: This is the core determinant of the treatment direction. Staging clarifies:
  - **Depth of Tumor Invasion (T)**: Whether the tumor is confined to the mucosa (very early stage) or has invaded the muscle or deeper tissues.
  - **Lymph Node Involvement (N)**: Whether regional lymph nodes are affected.
  - **Distant Metastasis (M)**: Whether the cancer has spread to other parts of the body (e.g., liver, lungs).
- **Tumor Location and Size**: The position (upper, middle, or lower esophagus) and size influence the surgical approach and radiation planning.
- **Molecular and Biomarker Status**: With the advent of precision medicine, specific genetic mutations or biomarkers (e.g., PD-L1 expression) are considered to guide targeted therapy or immunotherapy.

**2. Patient-Related Factors**

- **Performance Status**: Assessing the function of vital organs (heart, lungs, liver, kidneys) to determine if the patient can tolerate surgery, chemotherapy, or radiotherapy.
- **Age and Comorbidities**: Age and the presence of other serious illnesses (e.g., heart disease, diabetes) affect the safety of treatment options.
- **Patient Preference**: When multiple equally effective options exist, the patient's quality of life goals and personal preferences are crucial factors.

**3. Treatment Goals**

- **Curative Intent**: Aiming to completely eradicate the tumor, typically for early-stage and some locally advanced cancers.
- **Palliative Intent**: When a cure is not possible, the goal shifts to controlling tumor growth, relieving symptoms (like dysphagia), prolonging survival, and maintaining quality of life.

**4. Multidisciplinary Team (MDT) Collaboration**

- The treatment plan is not decided by a single doctor but is discussed and formulated by a **Multidisciplinary Team (MDT)**. This team typically includes:
  - **Surgical Oncologist**: Assesses surgical feasibility.
  - **Medical Oncologist**: Manages chemotherapy, targeted therapy, and immunotherapy.
  - **Radiation Oncologist**: Plans radiation therapy.
  - **Pathologists and Radiologists**: Provide precise pathological and imaging diagnoses.
  - **Other Support Staff**: Such as dietitians and specialist nurses.

**In summary**, doctors integrate all the above information to tailor a personalized and effective treatment plan for you, based on the latest **evidence-based medicine** and **clinical guidelines**

**Q22: What is the probability of cure for early-stage esophageal cancer?**

**A:** The cure probability for early-stage esophageal cancer is relatively high, and is commonly measured by the "5-year survival rate."

- **Overall Data**: After standardized treatment, the 5-year survival rate for early-stage esophageal cancer (where the tumor is confined to the mucosa or submucosa) can reach **70% - over 90%**. Some data even suggest that when the cancer is strictly limited to the esophageal mucosa, the 5-year survival rate can be as high as **83.3% - 92.9%**.
- **Potential for Clinical Cure**: This means the vast majority of early-stage patients have a significant chance of long-term survival and even clinical cure. This contrasts sharply with advanced-stage esophageal cancer, where the 5-year survival rate is typically below 20%-30%.
- **Key Prognostic Factors**:
  - **Depth of Tumor Invasion**: This is one of the most important prognostic determinants. For cancers limited to the mucosa (the superficial layer), the 5-year survival rate can exceed **80%**. However, if the cancer invades the submucosa (a deeper layer), the risk of lymph node metastasis increases significantly, and the prognosis is much poorer.
  - **Lymph Node Involvement**: Patients with early-stage cancer and no lymph node metastasis have a significantly better prognosis.
  - **Treatment Method**: Treatment for early-stage esophageal cancer primarily involves surgery and endoscopic resection. Studies have shown that for eligible early-stage patients, endoscopic resection (e.g., ESD, EMR) offers long-term survival rates comparable to surgery, while being less invasive and allowing for faster recovery.

In summary, early detection and standardized treatment are the keys to improving the cure probability for esophageal cancer.

**Q23: What is the approximate survival rate for my stage of esophageal cancer?**

**A:** Survival rates for esophageal cancer are closely tied to the stage at which it is diagnosed. The following data, derived from large-scale clinical studies, can serve as a general reference. Please remember that these are statistical averages, and your individual prognosis should be discussed with your primary physician.

A study based on hospital registry data from China, involving 2,598 patients who underwent surgery for esophageal cancer, reported the following **5-year overall survival (OS) rates** by pathologic stage (0 to IV):

- **Stage 0:** 94.44%
- **Stage I:** 75.22%
- **Stage II:** 62.94%
- **Stage III:** 38.05%
- **Stage IV:** 12.72%

Another study focusing on Chinese patients with esophageal squamous cell carcinoma, using the AJCC/UICC 8th edition staging, reported the following **5-year OS rates**:

- **Stage IA:** 72.39%
- **Stage IB:** 62.37%
- **Stage IIA:** 51.96%
- **Stage IIB:** 41.26%
- **Stage IIIA:** 31.45%

Furthermore, some physicians cite guidelines indicating that the 5-year survival rate for Stage I patients is about 90%, while for Stage III and IV it is approximately 33% and 26%, respectively. For patients who undergo surgery, an analysis of a large U.S. database showed **10-year survival rates** of 44.7% for Stage I, 32.6% for Stage II, 20.3% for Stage III, and 14.4% for Stage IV disease.

**It is crucial to emphasize** that these figures are primarily based on patients who received surgical treatment. Survival rates can vary significantly based on factors such as age, sex, tumor type (e.g., squamous cell vs. adenocarcinoma), treatment modality, and response to therapy.

**Q24: Can esophageal cancer recur after treatment is completed?**

**A:**
Yes, esophageal cancer can recur even after completion of curative-intent treatment (surgery, chemoradiotherapy, immunotherapy, etc.). The risk depends on tumor stage, histology, treatment modality, molecular markers, and the patient’s general condition.

- **Patterns of recurrence**: Include locoregional recurrence (anastomotic site, residual esophagus, mediastinal or supraclavicular lymph nodes) and distant metastases (liver, lungs, bones, brain, etc.).
- **Timing**: Most recurrences occur within **2–3 years** after treatment; the risk decreases significantly after 5 years, but it never drops to zero.
- **Estimated rates**: For locally advanced esophageal cancer, even after neoadjuvant chemoradiotherapy plus surgery, the 5-year recurrence rate ranges from **30% to 50%**. For early-stage (T1) disease, it is relatively lower, around 10–20%.
- **Surveillance**: Therefore, rigorous and regular follow-up is mandatory (typically every 3–6 months for the first 1–2 years, then with longer intervals), including endoscopy, CT/PET-CT, and tumor markers, to detect recurrence early and enable potential curative re-treatment or palliative management.
- **Treatment after recurrence**: If recurrence is confirmed, options include re-irradiation, chemotherapy, targeted/immunotherapy, or supportive care. For localized recurrences, re-resection or local ablation may still be feasible in selected cases.

**Conclusion**: Recurrence is a real possibility, but regular surveillance and comprehensive management significantly improve outcomes after recurrence. Patients should not neglect long-term monitoring just because initial treatment has ended.

**Q25: What major factors affect the long-term prognosis of esophageal cancer patients?**

**A:** The long-term prognosis of esophageal cancer patients is influenced by a combination of factors, which can be broadly categorized into four main areas:

**1. Tumor-Related Factors**
These are the most critical determinants of prognosis.

- **TNM Stage**: This is the single most important prognostic factor. The more advanced the stage, the poorer the survival. For example, one study reported 5-year survival rates of 94.44%, 75.22%, 62.94%, 38.05%, and 12.72% for stages 0, I, II, III, and IV, respectively.
- **Pathological Characteristics**: This includes histological type (squamous cell carcinoma vs. adenocarcinoma), tumor grade (well-differentiated tumors have a better prognosis), tumor location, and size.
- **Metastasis**: The presence of lymph node or distant metastasis is a strong negative prognostic factor.

**2. Treatment-Related Factors**
Standardized and timely treatment is key to improving outcomes.

- **Treatment Modality**: Whether a patient receives surgery, radiotherapy, chemotherapy, or emerging therapies like immunotherapy, and their combination (e.g., neoadjuvant therapy), significantly impacts survival.
- **Timing of Treatment**: Early detection and treatment at an early stage lead to significantly better prognoses compared to late-stage diagnosis.
- **Surgical Factors**: The surgical approach (e.g., minimally invasive surgery) and margin status are also relevant.

**3. Patient-Related Factors**
The patient's overall health and baseline characteristics are also important.

- **Age and Sex**: Younger patients (e.g., <75 years or <60 years) and female patients generally have a better prognosis.
- **Nutritional and Performance Status**: Nutritional status and performance status (e.g., Karnofsky performance status) are significant prognostic indicators. Markers like Body Mass Index (BMI) and the albumin-to-globulin ratio also have predictive value.
- **Lifestyle**: Unhealthy habits like smoking and heavy alcohol use are well-established risk factors.

**4. Emerging Molecular and Inflammatory/Immune Biomarkers**
Advances in medicine are identifying more biomarkers for precise prognosis prediction.

- **Inflammatory/Immune Markers**: Examples include the neutrophil-to-lymphocyte ratio (NLR), platelet-to-lymphocyte ratio (PLR), systemic immune-inflammation index (SII), and prognostic nutritional index (PNI).
- **Molecular Markers**: Information at the molecular level, such as specific gene mutations or protein expressions, is increasingly being incorporated into prognostic models.

**Q26: What are the common side effects during chemotherapy, radiotherapy, targeted therapy, or immunotherapy for esophageal cancer, and how should I manage them?**

**A:**

Different treatments for esophageal cancer have different side effect profiles, but most are manageable. Below is a breakdown by treatment type:

**I. Chemotherapy – Common Side Effects and Management**

- **Gastrointestinal reactions (nausea, vomiting, loss of appetite)**: This is one of the most common side effects of chemotherapy. Chemotherapy drugs can damage digestive tract cells and affect taste and smell.
  - **Management**: Discuss anti-nausea medications with your doctor; try eating small, frequent meals with easily digestible, nutritious foods; maintain a positive mindset; consider consulting a dietitian.
- **Oral mucositis (mouth sores)**: Chemotherapy can cause painful mouth sores that affect eating.
  - **Management**: Maintain good oral hygiene with mild mouthwashes; choose soft or liquid foods; avoid irritating foods.
- **Myelosuppression (bone marrow suppression)**: Can lead to decreased white blood cells and platelets, increasing infection and bleeding risks.
  - **Management**: Have regular blood count monitoring; practice good personal hygiene to prevent infections; seek immediate medical attention if fever or signs of infection occur.
- **Hair loss**: Chemotherapy drugs affect hair follicle cells.
  - **Management**: Hair typically grows back after treatment ends; use mild hair care products and avoid harsh treatments.
- **Peripheral neuropathy (numbness/tingling in hands and feet)**: Certain chemotherapy drugs (e.g., cisplatin, oxaliplatin, paclitaxel) can damage peripheral nerves.
  - **Management**: Report symptom changes to your doctor; take precautions to avoid burns or falls; doctors may adjust drug dosage if symptoms are severe.
- **Fatigue**: Extreme tiredness is common during chemotherapy.
  - **Management**: Get adequate rest and balance activities; maintain good nutrition.

**II. Radiotherapy – Common Side Effects and Management**

Radiotherapy side effects typically begin about a week after treatment starts and gradually worsen during treatment.

- **Radiation esophagitis (painful swallowing, difficulty swallowing)**: Most patients develop this 2–3 weeks into radiotherapy. Symptoms include swallowing pain and a burning sensation behind the breastbone.
  - **Management**: Eat bland, easily digestible liquid or semi-liquid foods (e.g., congee, noodles); avoid spicy, rough, or very hot foods; for severe cases, use mucosal protectants and pain medications as prescribed; drink about 3 liters of water daily; sit upright while eating; take small bites and chew slowly.
- **Radiation dermatitis (skin reactions)**: Skin in the treated area may show redness, dryness, peeling, etc..
  - **Management**: Keep skin clean and dry; avoid friction and scratching; gently wash with warm water, avoiding soap and alcohol; wear loose, soft cotton clothing.
- **Radiation pneumonitis (cough, shortness of breath)**: Chest radiotherapy can cause lung damage.
  - **Management**: Rest and avoid respiratory infections; for severe symptoms, corticosteroids may be needed as prescribed.
- **Fatigue**: Fatigue is very common during radiotherapy and may persist for weeks or months after treatment ends.
  - **Management**: Rest when needed; gentle exercise like walking may help boost energy.
- **Esophageal stricture**: In rare cases, radiotherapy can cause fibrosis and narrowing of the esophagus.
  - **Management**: If persistent swallowing difficulties occur, doctors may perform endoscopic dilation.

**III. Targeted Therapy – Common Side Effects and Management**

Side effects of targeted therapy depend on the specific drug used; they are generally milder than chemotherapy and usually resolve within weeks after treatment ends.

- **Skin rash**: Common with oral targeted drugs such as anlotinib and apatinib.
  - **Management**: Consult your doctor about topical creams (e.g., urea ointment); keep skin clean and avoid irritants.
- **Hand-foot syndrome**: Redness, peeling, and pain on palms and soles.
  - **Management**: Avoid friction and excessive heat on hands/feet; use mild moisturizers; doctors may recommend traditional Chinese medicine adjuncts.
- **Other possible side effects**: Fever, chills, headache, cough – usually mild.

**IV. Immunotherapy – Common Side Effects and Management**

Immunotherapy works by activating the immune system to attack tumors, but it can also cause immune-related adverse events (irAEs). Most patients experience mild, manageable symptoms.

- **Skin reactions (rash, pruritus)**: Among the most common immune-related side effects.
  - **Management**: Mild rashes can be managed with moisturizers and antihistamines; severe cases require medical attention and may need treatment interruption or corticosteroids.
- **Immune-related pneumonitis (cough, shortness of breath, fever)**: A rare but potentially life-threatening side effect.
  - **Management**: Report any new or worsening respiratory symptoms to your doctor immediately.
- **Immune-related colitis (diarrhea, colitis)**: Immunotherapy can cause intestinal inflammation.
  - **Management**: Mild diarrhea can be managed with dietary adjustments and hydration; moderate to severe diarrhea requires medical attention, possibly with antidiarrheals or corticosteroids.
- **Immune-related hepatitis, myocarditis, endocrinopathies (e.g., thyroid dysfunction)**: Though relatively rare, these can be life-threatening.
  - **Management**: Regular monitoring of liver/kidney function, thyroid function, and cardiac enzymes is essential during treatment; seek immediate care if symptoms like fatigue, jaundice, or palpitations occur.

**Important Reminders**:

1. **Maintain close communication with your medical team**: Report any changes in your condition promptly so treatment plans can be adjusted.
2. **Seek immediate care for severe side effects**: Such as severe anemia, breathing difficulties, severe infections, or heart failure (Grade 3–4 adverse events).
3. **Nutritional support is crucial**: Ensure adequate nutrition during treatment; consult a dietitian if needed.
4. **Most side effects are temporary**: They generally improve after treatment ends

**Q27: How should I maintain my nutrition during treatment for esophageal cancer?**

**A:** Esophageal cancer itself, along with treatments such as surgery, chemotherapy, and radiation therapy, often causes difficulty swallowing, loss of appetite, nausea, and vomiting, making patients highly susceptible to malnutrition. Malnutrition can reduce treatment tolerance, increase complication risks, and negatively impact treatment outcomes and quality of life. Therefore, nutritional support is not merely an adjunct but a core component of treatment, on par with surgery, chemotherapy, and radiation therapy. Below are the core principles and specific methods for maintaining nutrition at different stages of treatment.

**I. General Principles: Whole-Course Management with Individualized Intervention**

Patients should undergo **nutritional risk screening and assessment** immediately after diagnosis, with an **individualized nutritional intervention plan** developed by a multidisciplinary team. Nutritional assessment and management should continue throughout the entire treatment process—before, during, and after treatment.

**Core Dietary Principles:**

- **High-calorie, high-protein**: Prioritize high-protein, high-calorie foods such as lean meat, fish, poultry, eggs, legumes, and dairy products to maintain weight and prevent muscle wasting. For patients at risk of malnutrition, protein intake should be increased to **1.2 to 2 times** the normal requirement.
- **Small, frequent meals**: Avoid eating too much at once; aim for **6 to 8 small meals per day** instead of 3 large meals.
- **Soft, lukewarm, easily digestible foods**: Avoid hard, rough, extremely hot, or spicy foods.

**II. Nutritional Management at Different Treatment Stages**

**1. Before Treatment (Prehabilitation Phase)**

- **Nutritional reserve**: Strengthen nutritional reserves before surgery or chemoradiotherapy. If malnutrition is present (e.g., weight loss ≥10% in 6 months, BMI ≤18.5, etc.), **7 to 14 days of nutritional therapy** is recommended before surgery.
- **Immunonutrition**: The ESPEN guidelines recommend **5 to 7 days of immunonutrition** support before esophageal cancer surgery.
- **Prehabilitation**: A multimodal prehabilitation program including nutrition, exercise, and psychological support is recommended **2 to 4 weeks** before surgery.

**2. During Surgery**

**Early Postoperative Period (Days 1–5):**

- Oral intake is typically **not permitted**; nutrition is provided via intravenous infusion or feeding tube (nasogastric/nasojejunal tube).
- A **enteral feeding pump** is recommended for tube feeding, with an initial rate of **15–20 mL/hour**, gradually adjusted.
- The patient should remain **semi-upright (head elevated ≥30°)** during tube feeding to prevent reflux and aspiration.

**Postoperative Recovery Phase (from approximately Day 6):**

- Diet should progress **gradually**: clear liquids → full liquids → semi-liquids → soft foods → regular foods.
- Start with **50 mL** per meal, gradually increasing to **150–200 mL** as tolerated, with **5 to 6 meals per day**.
- Soft foods are recommended for approximately **3 to 6 months** after surgery, until the anastomotic scar stabilizes.
- Stomach capacity is reduced after surgery, leading to early satiety—special attention should be paid to eating small, frequent meals.
- Food temperature should be **below 50°C (122°F)** to avoid burning the anastomotic site.

**3. During Chemotherapy and Radiation Therapy**

- Nutritional therapy should be selected **in a standardized, individualized manner** based on nutritional assessments (e.g., PG-SGA score) and the grade of acute chemoradiotherapy toxicity before, during, and after treatment.
- **Weight monitoring**: Weigh yourself **at least twice a week** (in the morning, on an empty stomach, after bowel movement, wearing similar clothing). If weight drops **more than 1–2 kg in a week**, or **more than 5% in a month**, notify your doctor or dietitian immediately.
- If swallowing difficulty is severe, a **feeding tube** may be necessary to ensure nutritional intake.
- Radiation-induced damage to the esophageal mucosa can persist for weeks or even months. For **six months after radiotherapy**, continue with a soft, low-residue, easily digestible diet with multiple small meals daily, and avoid hard or fried foods.

**III. Nutritional Support Pathways – The "Five-Step Ladder" Principle**

Standardized treatment of malnutrition follows the **five-step ladder principle**, progressing upward as needed:

| Step | Method | Indication |
| --- | --- | --- |
| Step 1 | Diet + Nutrition Education | For patients with good nutritional status |
| Step 2 | Diet + Oral Nutritional Supplements (ONS) | For mild malnutrition |
| Step 3 | Total Enteral Nutrition (Tube Feeding) | Via nasogastric/nasojejunal tube |
| Step 4 | Partial Enteral + Partial Parenteral Nutrition | When enteral intake is insufficient |
| Step 5 | Total Parenteral Nutrition (IV) | For severe gastrointestinal dysfunction |

**Principle**: As long as the patient has partial gastrointestinal digestive and absorptive function, **enteral nutrition should be the first choice**. If the current step cannot meet **60%** of the target energy requirement for **3 to 5 days**, advancement to the next step is indicated.

**IV. Key Monitoring Indicators**

- **Weight**: The most intuitive and sensitive indicator
- **Body Mass Index (BMI)**: BMI < 18.5 indicates malnutrition risk
- **Handgrip strength**: Reflects muscle strength and physical function
- **Hemoglobin and albumin levels**: Reflect anemia and protein reserves
- **Food intake**: Whether daily intake reaches ≥60% of the target requirement

**V. Special Reminders**

1. **Seek professional help promptly**: Work with a clinical dietitian to develop a dietary plan.
2. **Do not force oral intake**: If swallowing is severely painful or oral intake is significantly insufficient, accept tube feeding or other nutritional support in a timely manner.
3. **Continue nutritional care after treatment ends**: Nutritional support should not stop abruptly when treatment ends; continue to ensure weight stability.
4. **Supplement nutrients as advised**: Under medical guidance, consider supplementation with omega-3 fatty acids, which may help reduce inflammation and improve nutritional status.

**Q28: What are the general stages for gradually resuming eating after esophageal cancer surgery?**

**A:** The recovery of oral intake follows a stepwise progression. The timeline varies per individual, but generally includes the following stages:

1. **Strict Fasting (Approximately Days 1-6 Post-Surgery)**:
   - You will be **nil by mouth**. All nutrition is provided via an **enteral feeding tube** (e.g., jejunostomy tube) to allow the surgical anastomosis to heal.
2. **Clear Liquid Diet (Approximately Days 7-10 Post-Surgery)**:
   - After your doctor confirms no leakage from the anastomosis.
   - **Examples**: Rice water, fat-free clear broths, thin strained fruit juice.
   - **Key Points**: Start with **30-50 ml** per serving, every 2-3 hours. Keep food at body temperature (around 37°C-40°C). Nutrition is still mainly from the feeding tube.
3. **Full Liquid Diet (Approximately Weeks 1-2 Post-Surgery)**:
   - Progress to a **full liquid** diet. Foods should be liquid or pureed, **smooth, and easily pourable**.
   - **Examples**: Milk, soy milk, thin custard, egg drop soup, commercial liquid supplements.
   - **Key Points**: Follow **small, frequent meals** – about **6-8 meals per day**, **100-200 ml** each.
4. **Semi-Solid (Pureed) Diet (Approximately Weeks 2-4 Post-Surgery)**:
   - Transition to a **semi-solid** diet. Foods should be **soft, moist, and require minimal chewing**.
   - **Examples**: Congee, soft noodles, smooth scrambled eggs, meat puree, mashed potatoes.
   - **Key Points**: Gradually increase food thickness every **2-3 days**. Monitor your body's response to each increase.
5. **Soft Diet (Approximately 1 Month Post-Surgery and Beyond)**:
   - Once you tolerate pureed food well, you can start **soft solids** as a transition to a normal diet.
   - **Examples**: Soft rice, steamed buns, steamed fish, soft cooked vegetables.
   - **Key Points**: Foods must be **cooked until very soft and cut into small pieces**.
   - Returning to a relatively normal diet can take **3 to 6 months** or longer.

Regardless of the stage, these principles are crucial:

- **Eat Small, Frequent Meals**: Your stomach capacity is reduced. Aim for **5-8 small meals** a day, stopping when you are about 70-80% full.
- **Chew Thoroughly**: Chew each mouthful **at least 20-30 times** until it becomes a paste before swallowing.
- **Maintain Proper Posture**: Sit **upright or in a semi-recumbent position** (elevate head of bed 30°-45°) while eating. **Remain upright for at least 30-60 minutes** after a meal and **avoid lying flat for 2 hours** to prevent reflux.
- **Optimal Food Temperature**: Consume foods and liquids **warm** (around body temperature) to avoid irritating the digestive tract.
- **Separate Liquids from Solids**: Drink fluids **between meals** rather than with meals to prevent feeling full too quickly.
- **Food Choices**:
  - **Choose**: High-protein (fish, poultry, eggs, lean meat), high-calorie, high-vitamin, and easily digestible foods.
  - **Avoid**: **Hard, coarse, or stringy** foods (nuts, fried foods, gristly meat), **spicy** foods (chili, garlic), and very **sweet or high-fat** foods.
- **Warning Signs**: Seek immediate medical attention if you experience **persistent vomiting, severe chest pain, severe coughing or breathing difficulties** while eating.

**Q29: How should the pain caused by esophageal cancer be managed?**

**A:** The management of esophageal cancer pain should follow the principles of **comprehensive assessment, multidisciplinary collaboration, and individualized treatment**, combining pharmacological and non-pharmacological approaches.

**I. Pain Assessment**

The first step is scientific pain assessment, following the principles of **"routine, quantitative, comprehensive, and dynamic"** evaluation. Standardized scales should be used to assess pain intensity, nature (e.g., dull ache, burning, stabbing), and its impact on physical, psychological, and social function. Esophageal cancer pain can arise from tumor compression, tissue invasion, nerve infiltration, treatment side effects (surgery, radiotherapy, chemotherapy), or complications such as ulcers and obstruction.

**II. Pharmacological Treatment**

Pharmacological therapy is the cornerstone of cancer pain management, following the WHO **three-step analgesic ladder**, with individualized adjustments:

- **Mild pain**: Non-opioid analgesics such as paracetamol (acetaminophen) or NSAIDs (e.g., ibuprofen).
- **Moderate pain**: Weak opioids (e.g., codeine, tramadol), or low-dose strong opioids combined with non-opioids.
- **Severe pain**: **Strong opioids** (e.g., morphine, oxycodone, fentanyl) for systemic analgesia. Individualized dose titration is required, with close monitoring and management of adverse effects such as constipation, nausea, and sedation.
- **Adjuvant medications**: For neuropathic pain (e.g., burning or tingling sensations), anticonvulsants (e.g., gabapentin, pregabalin) or antidepressants (e.g., duloxetine) may be added to enhance analgesic efficacy.

**III. Non-Pharmacological and Interventional Approaches**

- **Non-pharmacological therapies**: Include **relaxation techniques, music therapy, and psychological interventions** to alleviate tension and reduce pain perception. **Acupuncture and acupressure** (traditional Chinese medicine external therapies) can help relieve pain by promoting energy flow. **Positioning adjustments** (e.g., semi-recumbent or sitting positions) can reduce pressure on surrounding tissues.
- **Interventional analgesic techniques**: For refractory pain uncontrolled by medication, **nerve blocks** (e.g., thoracic paravertebral nerve block, epidural analgesia) and other minimally invasive techniques may be considered. If severe uncontrolled pain occurs after **esophageal stent placement**, endoscopic stent removal should be considered.

**IV. Etiological Treatment**

Targeted antitumor therapy itself can effectively relieve cancer pain. For patients with locally advanced or symptomatic esophageal cancer, **palliative radiotherapy** can effectively alleviate pain, dysphagia, and other symptoms. Radiotherapy combined with chemotherapy or immunotherapy may further improve pain control.

**V. Patient and Family Education**

Patients should promptly report their pain to the healthcare team. Family members can assist in keeping a **pain diary** to help clinicians adjust treatment plans. For diet, choose **soft, easily digestible** foods (e.g., porridge, pureed foods, custards) and avoid spicy, rough, extremely hot, or cold foods that may irritate the esophagus. **Chew slowly and sit upright** during meals to reduce eating-related pain.

**Q30: How often should I have follow-up examinations after treatment for esophageal cancer, and what items are included?**

**A:**

Follow-up care after esophageal cancer treatment is essential. The frequency and specific tests depend largely on the cancer's stage, the type of treatment received, and your individual risk of recurrence. The following is a summary based on general guidelines from major cancer organizations.

**I. Follow-up Schedule**

The risk of esophageal cancer recurrence is highest within the first two years after treatment, which is why follow-up is most frequent during this period. A typical schedule is:

- **First 1-2 years after treatment**: Every 3 months. Some guidelines suggest a range of every 3 to 6 months.
- **Years 3-5 after treatment**: Every 6 months. Some guidelines suggest a range of every 6 to 12 months.
- **More than 5 years after treatment**: Once a year.

**Important Notes**:

- If you received adjuvant chemotherapy or radiation after surgery, your first follow-up is usually about one month after all treatments are completed, after which you will follow the regular schedule above.
- **Do not wait** for your scheduled appointment if you experience any new symptoms, such as difficulty swallowing, chest pain, or unexplained weight loss. Contact your doctor immediately.
- For patients who achieve a complete clinical response after neoadjuvant therapy and choose "active surveillance" instead of immediate surgery, follow-up may be more intensive, e.g., with endoscopy and CT scans every 3 months.

**II. Follow-up Tests and Examinations**

The goals of follow-up are to detect any potential recurrence or metastasis early and to monitor and manage long-term side effects of treatment. Routine examinations generally include:

1. **Medical History and Physical Exam**: Your doctor will ask about any new symptoms and perform a physical examination.
2. **Imaging Tests**:
   - **Contrast-Enhanced CT Scan**: This is the most critical routine imaging test, usually covering the neck, chest, and abdomen, to check for recurrence or metastasis.
   - **Ultrasound of the Neck and Abdomen**: Used to check lymph nodes in the neck and organs in the abdomen (like the liver, spleen, and kidneys) for metastasis, complementing the CT scan.
3. **Laboratory Tests (Blood Work)**:
   - **Complete Blood Count and Comprehensive Metabolic Panel**: To assess your overall health, nutritional status, and organ function (e.g., liver and kidneys).
   - **Tumor Markers**: Such as SCC, CEA, and CA19-9. These can be used as supplementary tools to help monitor for changes.
4. **Endoscopy (Gastroscopy)**: Used to directly visualize the anastomosis (surgical connection) and the lining of the esophagus and stomach. It is the gold standard for diagnosing local recurrence or strictures. If there are no specific concerns, it is often recommended once a year. For patients treated with definitive chemoradiation, more frequent endoscopies may be recommended.

**Other Tests Used as Needed:**

- **Upper GI Series (Barium Swallow)**: Used when a stricture or recurrence at the anastomosis is suspected.
- **PET-CT**: Not a routine test, but may be used when suspicious findings are found on other scans or for a more detailed whole-body assessment.
- **Bone Scan**: Used when symptoms like bone pain suggest possible metastasis to the bones.

**Important Note**: This information is for educational purposes and should not replace professional medical advice. Your specific follow-up plan and tests should always be discussed and determined by your primary oncologist.

**Doubao**

Q1: What are the main risk factors for esophageal cancer?

A: The main risk factors include the following aspects:

1. Lifestyle factors: Long-term heavy drinking, smoking, frequent consumption of overly hot food and beverages.
2. Dietary factors: Regular intake of pickled food, mouldy food, food containing nitrosamines; insufficient intake of fresh fruits and vegetables.
3. Disease-related factors: Gastroesophageal reflux disease, Barrett’s esophagus, esophageal leukoplakia, esophageal achalasia and other precancerous lesions or chronic esophageal diseases.
4. Genetic and demographic factors: Family history of esophageal cancer; advanced age; males have a higher incidence than females.
5. Other factors: Malnutrition, deficiency of trace elements such as selenium, poor oral hygiene.

Q2: What are the differences in causes between esophageal squamous cell carcinoma and adenocarcinoma?

A:

1. Esophageal squamous cell carcinoma (ESCC)

The major risk factors include long-term heavy alcohol consumption, tobacco smoking (cigarettes, chewing tobacco), intake of hot food and beverages, poor dietary nutrition (deficiency of vitamins, zinc, selenium), consumption of pickled, mold-contaminated food containing nitrosamines, human papillomavirus (HPV) infection, as well as genetic susceptibility. This tumor mainly arises from the stratified squamous epithelium covering the upper and middle esophagus.

2. Esophageal adenocarcinoma (EAC)

Its primary precursor lesion is Barrett’s esophagus, which develops on the basis of chronic gastroesophageal reflux disease (GERD). Key risk factors consist of chronic acid reflux, obesity (especially central obesity), smoking, a diet high in fat and low in fiber, and low Helicobacter pylori infection rate. It originates from the metaplastic columnar epithelium, mostly located in the lower esophagus near the gastroesophageal junction.

Core causal distinction: ESCC is predominantly induced by exogenous toxic stimuli acting on normal squamous epithelium; EAC stems from long-term acid reflux leading to epithelial metaplasia and subsequent malignant transformation.

Q3: What are the precancerous lesions of esophageal cancer?

A: The main precancerous lesions of esophageal cancer include esophageal squamous intraepithelial neoplasia and Barrett esophagus.

1. Esophageal squamous intraepithelial neoplasia (SIN): It is the primary precancerous lesion of esophageal squamous cell carcinoma, further divided into low-grade squamous intraepithelial neoplasia and high-grade squamous intraepithelial neoplasia. High-grade lesions have a significantly higher risk of progressing to invasive cancer.
2. Barrett esophagus: It is the typical precancerous lesion of esophageal adenocarcinoma, characterized by intestinal metaplasia of esophageal columnar epithelium. It can be accompanied by intraepithelial neoplasia, and the risk of canceration rises with the severity of dysplasia.

Other related high-risk pathological changes include chronic erosive esophagitis, esophageal leukoplakia, esophageal papilloma, esophageal stricture caused by long-term corrosive injury, etc. These lesions have potential malignant transformation tendency and require regular endoscopic follow-up.

Q4: Is esophageal cancer hereditary?

A: Esophageal cancer is not a typical hereditary disease. It does not directly pass from parents to offspring in a simple genetic pattern. However, genetic susceptibility exists. If immediate family members have esophageal cancer, an individual’s risk will be slightly higher. Most cases arise from the combined effect of genetic predisposition and environmental risk factors such as long-term smoking, heavy drinking, unhealthy diet, and chronic esophageal inflammation.

Question5: What is the peak age of onset for esophageal cancer?

Answer: The peak age of onset for esophageal cancer is between 60 and 70 years old.

Q6: What early symptoms of esophageal cancer should I be alert for?

A: Early symptoms of esophageal cancer are often atypical, intermittent and easy to ignore. You should pay close attention to the following manifestations:

1. A sense of obstruction or choking when swallowing food, which is more obvious when eating dry and hard food.
2. Discomfort behind the sternum, including dull pain, burning sensation or foreign body sensation.
3. Feeling that food moves slowly when swallowing, as if sticking to the esophageal wall.
4. Dryness and tightness in the throat, aggravated especially after eating rough food.
5. Intermittent acid regurgitation, belching and upper abdominal distension.

⚠️Important reminder: These symptoms do not mean you have esophageal cancer. Esophagitis and gastroesophageal reflux disease can cause similar discomfort. If symptoms last for more than two weeks or worsen repeatedly, please undergo gastroscopy as soon as possible for a definite diagnosis.

Q7: What are the clinical manifestations of esophageal cancer?

A:

Early-stage manifestations

Patients may have mild, intermittent atypical symptoms: retrosternal discomfort, burning sensation, foreign body sensation in the esophagus, mild dysphagia when eating dry and hard food, or transient food stagnation feeling. Many early patients have no obvious symptoms.

Progressive (middle and late-stage) typical manifestations

1. Progressive dysphagia: The most classic symptom. Initially difficult to swallow solid food, then semi-liquid food, and finally unable to swallow liquid food and saliva.
2. Retrosternal pain or back pain: Persistent dull pain, often suggesting tumor invasion of surrounding tissues.
3. Regurgitation of food and mucus: Undigested food, mucus, or occasional blood may be vomited.

Advanced metastatic and complication-related manifestations

1. Bleeding: Tumor ulceration can cause hematemesis or melena.
2. Hoarseness: Tumor invades or compresses recurrent laryngeal nerve.
3. Persistent cough, aspiration pneumonia: Tracheoesophageal fistula formation.
4. Weight loss, fatigue, cachexia: Long-term insufficient intake and tumor consumption.
5. Signs of distant metastasis: Lymph node enlargement in neck, bone pain, liver mass, etc.

Q8: How can I tell whether my esophageal cancer symptoms are worsening?

A: You can judge from changes in your main symptoms and physical condition.

1. Difficulty swallowing gets worse: It gradually becomes harder to swallow solid food, then semi-liquid food, and finally even liquids and saliva. You may choke frequently.
2. Increased pain: Persistent pain in the chest, behind the sternum or upper abdomen. Pain may occur when swallowing, or exist continuously even without eating.
3. Unintentional weight loss and fatigue: You lose weight rapidly without dieting, accompanied by persistent weakness, lack of energy.
4. New abnormal symptoms: Constant regurgitation, blood in saliva or stool, persistent cough, hoarseness, fever, difficulty breathing.
5. Poor appetite: Severe loss of appetite, feeling full quickly after eating a small amount.

If you notice any of the above changes, contact your oncologist or gastroenterologist promptly for examination.

Q9: Which organs does esophageal cancer most commonly spread to, and what symptoms occur after metastasis?

A: Esophageal cancer mainly spreads through lymphatic metastasis first, followed by hematogenous metastasis.

1. Common metastatic sites

- Lymph nodes: Cervical, mediastinal, abdominal para-aortic lymph nodes are the most frequent metastatic locations.
- Distant hematogenous metastasis organs: Liver, lung, bone, and adrenal glands are the most common sites; brain metastasis can also be seen occasionally.

2. Symptoms caused by metastasis

- Lymph node metastasis: Mediastinal lymph node enlargement may compress the recurrent laryngeal nerve, leading to hoarseness; compression of the trachea can cause cough, shortness of breath and dyspnea.
- Liver metastasis: Right upper abdominal pain, liver enlargement, jaundice, ascites, loss of appetite.
- Lung metastasis: Persistent cough, hemoptysis, chest pain, dyspnea.
- Bone metastasis: Persistent bone pain, pathological fractures, spinal metastasis may cause limb numbness or even paraplegia due to spinal cord compression.
- Brain metastasis: Headache, vomiting, dizziness, limb weakness, visual disturbance, convulsions.
- Adrenal metastasis: Many patients have no obvious symptoms in the early stage; large lesions may cause fatigue and hypertension.

Question10: What emergency situations in patients with esophageal cancer require immediate visit to the emergency department?

Answer:

1. Massive esophageal hemorrhage: Sudden hematemesis, massive melena, accompanied by palpitations, dizziness, cold sweats and decreased blood pressure, with risk of hemorrhagic shock.
2. Esophageal perforation or esophagotracheal fistula: Severe persistent chest pain, fever, violent choking after eating or drinking, subcutaneous emphysema in the neck or chest. Aspiration of food may lead to severe pneumonia.
3. Complete esophageal obstruction: Total inability to take food or water, persistent drooling and severe nausea and vomiting, which may rapidly cause dehydration and electrolyte disorders.
4. Severe airway compression and dyspnea: Tumor compression of the airway resulting in chest tightness, wheezing, cyanosis and inability to lie flat.
5. Uncontrolled severe pain: Persistent severe chest or back pain unrelieved by routine analgesics.
6. Severe vomiting complicated with dehydration: Frequent vomiting, dry mouth, markedly reduced urine output and altered mental status.
7. High fever and infection: Persistent high fever, which suggests life-threatening infections such as mediastinitis secondary to perforation or severe pulmonary infection.

Q11: Which department should I visit after being diagnosed with esophageal cancer?

A: 1. Thoracic Surgery: If the tumor is in the early or middle stage and surgical resection is feasible.

2. Medical Oncology: Suitable for patients needing chemotherapy, targeted therapy, immunotherapy, or advanced patients who cannot undergo surgery.

3. Radiation Oncology: For patients who require radiotherapy or chemoradiotherapy.

4. Gastroenterology: Can be consulted for endoscopic treatment of early esophageal cancer.

You can first consult the thoracic surgery or medical oncology department according to your disease stage.

Question12: What is the gold standard for diagnosing esophageal cancer?

Answer: Endoscopy with biopsy histopathological examination is the gold standard for diagnosing esophageal cancer.

Question13: After being diagnosed with esophageal cancer, what additional tests are needed to determine the severity of the disease?

Answer:

A series of staging examinations are required after the diagnosis of esophageal cancer to evaluate the depth of tumor invasion, lymph node metastasis and distant metastasis so as to judge the severity of the disease. The main examinations are as follows:

1. Contrast-enhanced CT of the chest and abdomen: It assesses the size and extramural invasion of esophageal tumors, detects metastasis of mediastinal and abdominal lymph nodes, and screens metastases in organs such as the liver and adrenal glands.
2. Endoscopic Ultrasound (EUS): It accurately identifies the layers of the esophageal wall invaded by tumors, differentiates early esophageal cancer from locally advanced disease, and evaluates involvement of adjacent lymph nodes.
3. Contrast-enhanced CT of the neck: To screen cervical lymph node metastasis.
4. PET-CT (selected as appropriate): It provides whole-body evaluation to detect occult distant metastatic lesions. It is commonly used for patients with unclear staging or those planned for surgery and comprehensive therapy.
5. Bone scan (if bone metastasis is suspected): To confirm the presence of bone metastasis.
6. Brain imaging examination (if brain metastasis is suspected): To rule out brain metastasis.

In addition, basic laboratory tests including blood routine, liver and kidney function and tumor markers will be completed to evaluate the general physical condition of patients and guide the formulation of subsequent treatment plans.

Q14: What stage is my esophageal cancer?

A: It is impossible to determine the stage of your esophageal cancer directly from this sentence. Cancer staging requires comprehensive results from gastroscopy, enhanced CT, pathological biopsy, and other examinations. You need to consult your attending doctor, who will judge the specific stage according to the tumor size, depth of invasion, lymph node metastasis and distant metastasis.

Q15: What do the HER2, PD L1, dMMR/MSI, and Claudin18.2 indicators mean in the esophageal cancer pathology report?

A:

Q1: What does HER2 mean in esophageal cancer pathology reports?

A1: HER2 (Human Epidermal Growth Factor Receptor 2) is a key therapeutic target, mainly relevant for esophageal adenocarcinoma.

Results are classified as negative, equivocal or positive. Positive status indicates HER2 protein overexpression or gene amplification.

Clinically, patients with HER2-positive advanced esophageal adenocarcinoma are eligible for anti-HER2 targeted therapies such as trastuzumab. Benefits of HER2-targeted drugs are limited in esophageal squamous cell carcinoma.

Q2: What does PD-L1 mean in esophageal cancer pathology reports?

A2: PD-L1 (Programmed Death-Ligand 1) is a critical biomarker for immunotherapy. Its expression is usually reported as the CPS score (Combined Positive Score).

A higher CPS means higher PD-L1 expression in tumor tissues. Patients with high PD-L1 expression are more likely to benefit from PD-1/PD-L1 immune checkpoint inhibitors, which guides immunotherapy selection for esophageal cancer.

Q3: What do dMMR and MSI mean in esophageal cancer pathology reports?

A3: MMR refers to mismatch repair proteins; MSI stands for microsatellite instability. They are correlated biomarkers.

- dMMR: deficient mismatch repair, corresponding to MSI-H (microsatellite instability-high)
- pMMR: proficient mismatch repair, corresponding to MSS (microsatellite stable)

The frequency of dMMR/MSI-H is relatively low in esophageal cancer. Patients with dMMR/MSI-H usually respond well to single-agent PD-1 immunotherapy.

Q4: What does Claudin 18.2 mean in esophageal cancer pathology reports?

A4: Claudin 18.2 is a tight junction protein subtype expressed mainly in gastric and esophageal adenocarcinoma cells.

A positive result means the tumor cell membrane expresses Claudin 18.2, making patients candidates for Claudin 18.2-targeted agents (e.g., zolbetuximab). This biomarker applies primarily to esophageal adenocarcinoma; positive rates are extremely low in esophageal squamous cell carcinoma.

Q5: General summary of these four biomarkers?

A5: All four are predictive biomarkers for precision treatment in advanced esophageal cancer, rather than markers for initial cancer diagnosis. They help screen suitable patients for targeted therapy or immunotherapy.

HER2 and Claudin 18.2 guide targeted medication; PD-L1 and dMMR/MSI predict the efficacy of immune therapy.

Q16: Is endoscopic resection suitable for early-stage esophageal cancer?

A: Endoscopic resection is an important minimally invasive treatment option for selected patients with early-stage esophageal cancer. It is mainly applicable to lesions limited to the mucosa, without obvious lymph node metastasis risk. Typical indications include tumors confined to the epithelial layer or lamina propria, with low differentiation risk and no invasion into deeper submucosa.

If the tumor invades the deep submucosa or carries a high risk of lymph node metastasis, endoscopic resection alone is insufficient, and additional radical esophagectomy or adjuvant therapy should be considered. Comprehensive evaluation of lesion size, depth of invasion, pathological type and differentiation status is required before treatment to select appropriate candidates.

Q17: What are the surgical approaches for esophageal cancer?

A: Surgical approaches for esophageal cancer mainly include open esophagectomy and minimally invasive esophagectomy:

1. Open esophagectomy

It consists of left thoracotomy esophagectomy, right thoracotomy (Ivor-Lewis) esophagectomy, and transhiatal esophagectomy without thoracotomy.

2. Minimally invasive esophagectomy (MIE)

This category covers thoracoscopic/laparoscopic esophagectomy, robotic-assisted minimally invasive esophagectomy. Common surgical procedures include McKeown three-incision minimally invasive esophagectomy, Ivor-Lewis minimally invasive esophagectomy.

In addition, palliative surgical procedures such as esophageal bypass surgery can be adopted for patients with advanced tumors who cannot undergo radical resection to relieve dysphagia.

Question18: Why is chemoradiotherapy needed before surgery for esophageal cancer?

Answer: Preoperative chemoradiotherapy (neoadjuvant chemoradiotherapy) is widely applied for locally advanced esophageal cancer for the following reasons:

1. It can shrink the primary tumor and reduce the volume of regional lymph node metastases, making the tumor easier to resect and improving the chance of achieving complete surgical resection (R0 resection).
2. It eliminates potential micro-metastases around the tumor and invisible distant tiny lesions, lowering the risk of postoperative tumor recurrence and metastasis.
3. It helps assess the tumor’s sensitivity to chemotherapy and radiotherapy, which provides a reference for subsequent postoperative treatment regimens.
4. Downstaging of tumors can increase the possibility of preserving organ function and avoid overly extensive surgical trauma in suitable patients.
5. Compared with postoperative treatment, neoadjuvant therapy delivers drugs and radiation to intact tumor tissue with better blood supply, often achieving superior anti-tumor effects.

Q19: What are the most commonly used chemotherapy, immunotherapy, and targeted therapy drugs for advanced esophageal cancer?

A:

1. Chemotherapy drugs

Common first-line chemotherapy regimens are based on platinum agents. Main drugs include cisplatin, carboplatin, oxaliplatin; combined with fluoropyrimidines such as 5-fluorouracil (5-FU), capecitabine. Paclitaxel, docetaxel are widely used as taxane chemotherapeutic agents, often applied in first-line or second-line treatment.

2. Immunotherapy drugs (PD-1 inhibitors, mainstream for esophageal squamous cell carcinoma)

- Pembrolizumab
- Nivolumab
- Sintilimab
- Camrelizumab
- Toripalimab

These anti-PD-1 monoclonal antibodies are used alone or combined with chemotherapy for first-line and second-line treatment of advanced esophageal squamous cell carcinoma.

3. Targeted therapy drugs

1. Anti-EGFR: Cetuximab
2. Anti-VEGF/VEGFR: Ramucirumab, apatinib
3. HER2-targeted agents (for HER2-positive esophageal adenocarcinoma): Trastuzumab, trastuzumab deruxtecan (T-DXd)

In esophageal squamous cell carcinoma, effective targeted drugs remain limited at present.

Question20: What treatment options are available for esophageal cancer from early to advanced stages?

Answer:

1. Early-stage esophageal cancer (limited to the esophageal wall, no lymph node metastasis)

Endoscopic therapy is the primary minimally invasive option, including endoscopic mucosal resection (EMR) and endoscopic submucosal dissection (ESD). For lesions unsuitable for endoscopy, surgical resection of the esophagus is adopted. Adjuvant treatment is usually unnecessary for most patients.

2. Locally advanced esophageal cancer (tumour invades deep tissues or accompanied by regional lymph node metastasis, no distant metastasis)

The main standard regimens include neoadjuvant chemoradiotherapy followed by surgery, or definitive concurrent chemoradiotherapy for patients who cannot tolerate surgery. Surgery alone is rarely used. Common chemotherapy agents include platinum combined with fluoropyrimidine drugs.

3. Metastatic/advanced esophageal cancer (distant organ metastasis)

Systemic treatment serves as the core. First-line treatment consists of chemotherapy; patients with PD-L1 expression or HER2 positivity can receive immunotherapy or targeted therapy combined with chemotherapy. Second-line and subsequent therapies involve single-agent chemotherapy, immune checkpoint inhibitors, or targeted drugs. Palliative radiotherapy can relieve symptoms such as dysphagia and bleeding. Supportive care including nutritional support is essential throughout treatment.

Q21: On what basis does the doctor formulate my treatment plan for esophageal cancer?

A: Doctors will develop your esophageal cancer treatment plan based on multiple key factors. They mainly consider the pathological type and differentiation degree of the tumor, the location, size, invasion depth and clinical stage of esophageal cancer. In addition, your physical condition, age, underlying diseases, organ function, nutritional status, as well as your personal willingness and expected treatment goals will also be taken into comprehensive consideration. Multidisciplinary consultation opinions will also be referenced when necessary.

Q22: What is the probability of cure for early-stage esophageal cancer?

A: The cure rate of early-stage esophageal cancer is relatively high. For patients with carcinoma in situ and stage I esophageal cancer without lymph node metastasis, the 5-year survival rate (commonly used clinical indicator to judge curative effect) can reach over 85%. Many patients can achieve radical cure after standardized endoscopic resection or surgical treatment. However, the specific prognosis is affected by tumor differentiation degree, lesion range, treatment timing and postoperative follow-up management. Regular reexamination is still required after treatment to prevent recurrence.

Question23: What is the approximate survival rate for my stage of esophageal cancer?

Answer: The survival rate of esophageal cancer varies greatly depending on the specific cancer stage, pathological type, treatment plan, physical condition and other individual factors. Survival statistics are only general population data and cannot predict your personal prognosis accurately.

You need to provide your exact esophageal cancer stage (Stage I / II / III / IV) to get corresponding reference data. It is strongly recommended that you consult your attending doctor, who can evaluate your situation comprehensively and give the most targeted judgment.

Q24: Can esophageal cancer recur after treatment is completed?

A: Yes, esophageal cancer may recur after treatment. Recurrence risk varies depending on cancer stage, pathological type, treatment method, tumor differentiation degree and individual physical condition. Early esophageal cancer has a relatively low recurrence probability after radical treatment, while patients with middle and advanced esophageal cancer face higher recurrence risks. Regular long-term follow-up examinations are essential to detect recurrent lesions at an early stage.

Question25: What major factors affect the long-term prognosis of esophageal cancer patients?

Answer:

1. Tumor stage at diagnosis is the most decisive factor. Early-stage esophageal cancer has a far better long-term survival rate, while locally advanced or metastatic disease usually leads to poor prognosis.
2. Pathological type and differentiation degree. Esophageal squamous cell carcinoma and adenocarcinoma have different biological behaviors; poorly differentiated tumors are more prone to invasion and metastasis.
3. Complete resection status. Patients who achieve radical surgical resection have significantly better long-term outcomes than those with residual tumors.
4. Lymph node metastasis status. The number and location of metastatic lymph nodes are strong predictors of long-term prognosis.
5. Treatment modality and compliance. Standardized comprehensive treatment (surgery, radiotherapy, chemotherapy, immunotherapy) and persistent treatment compliance help improve long-term survival.
6. Patient’s general physical condition, age and comorbidities. Severe underlying diseases may restrict anti-tumor treatment and increase mortality risks.
7. Postoperative complications, tumor recurrence and distant metastasis. Recurrence is the leading cause of unsatisfactory long-term prognosis.

Question26: What are the common side effects during chemotherapy, radiotherapy, targeted therapy, or immunotherapy for esophageal cancer, and how should I manage them?

Answer:

Q1: What common side effects occur with chemotherapy for esophageal cancer, and how to manage them?

A1:

Common side effects:

1. Gastrointestinal reactions: nausea, vomiting, loss of appetite, diarrhea or constipation.
2. Myelosuppression: decreased white blood cells, platelets and hemoglobin, raising infection and bleeding risks.
3. Mucosal injury: oral mucositis, esophageal inflammation.
4. Fatigue, hair loss, peripheral neuropathy (numbness/tingling in hands and feet).

Management:

- Antiemetic drugs are given prophylactically before chemotherapy; eat light, small frequent meals.
- Regular blood tests. Use granulocyte-stimulating factors or thrombopoietic agents when blood counts drop; avoid crowded places to prevent infection.
- Maintain oral hygiene; avoid spicy, hot, rough food.
- Get adequate rest; keep limbs warm for neuropathy; avoid cold stimulation for platinum-related numbness.

Q2: What common side effects occur with radiotherapy for esophageal cancer, and how to manage them?

A2:

Common side effects:

1. Radiation esophagitis (most typical): painful swallowing, dysphagia, chest pain, usually appearing 2–3 weeks after starting radiation.
2. Radiation pneumonitis: dry cough, shortness of breath.
3. Fatigue, dry mouth, loss of appetite.
4. Late adverse events: esophageal stricture in some patients.

Management:

- Consume soft, moist, finely chopped food; avoid irritant, hard, high-temperature food. Doctors may prescribe mucosal protectants and analgesics for painful swallowing.
- Report cough or dyspnea promptly for early screening of radiation pneumonitis; avoid smoking and dust exposure.
- Sip water frequently to relieve dry mouth; arrange rest periods every day.
- Follow regular post-radiation follow-up to detect esophageal stricture early.

Q3: What common side effects occur with targeted therapy for esophageal cancer, and how to manage them?

A3:

Common side effects vary by targets (HER2, VEGFR etc.):

1. Anti-HER2 agents: diarrhea, fatigue, cardiotoxicity (decreased left ventricular ejection fraction), rash.
2. Anti-angiogenic targeted drugs (anti-VEGFR): hypertension, proteinuria, hand-foot skin reaction, bleeding, gastrointestinal perforation risk.

Management:

- Monitor cardiac function regularly during anti-HER2 treatment; report chest tightness, palpitations immediately.
- Measure blood pressure daily for anti-angiogenic therapy; use antihypertensives as instructed. Check urine routine regularly to watch for proteinuria.
- Protect hands and feet: avoid friction, hot water soaking; apply moisturizer to relieve hand-foot reactions.
- Seek medical help quickly for black stool, hematemesis or severe abdominal pain to rule out bleeding/perforation.

Q4: What common side effects occur with immunotherapy (ICIs) for esophageal cancer, and how to manage them?

A4:

Side effects are immune-related adverse events (irAEs), which can affect any organ and may arise weeks to months after treatment initiation.

Common manifestations:

1. Fatigue, thyroid dysfunction (hypothyroidism or hyperthyroidism).
2. Pneumonitis: dry cough, chest tightness, shortness of breath.
3. Enterocolitis: persistent diarrhea, abdominal pain.
4. Skin reactions: rash, pruritus.
5. Less commonly: hepatitis, myocarditis, adrenal insufficiency.

Management:

- Regular thyroid function, liver function and imaging examinations.
- Immediately inform your oncologist once persistent cough, diarrhea, dyspnea or unexplained fatigue appears. Do not self-medicate.
- Mild irAEs may be controlled with symptomatic treatment; moderate-severe cases require hormone therapy and temporary suspension of immunotherapy under doctor guidance.
- Do not arbitrarily stop immunotherapy without physician evaluation.

Question27: How should I maintain my nutrition during treatment for esophageal cancer?

Answer:

1. Adjust eating habits

Eat small, frequent meals, 5–6 times a day or more. Do not wait until you feel hungry. Chew food thoroughly to ease the burden on the esophagus. Eat in a sitting or semi-upright position. Avoid lying down immediately after meals to prevent reflux and choking.

2. Choose food with appropriate texture

Select diets in the order of liquid, semi-liquid and soft food according to your swallowing ability. Prefer mushy and tender food. Avoid dry, rough, high-fiber and fried food that may injure the narrowed esophagus. You can blend meals into homogenized food with a blender if needed.

3. Consume enough high-quality protein and calories

Protein supports mucosal repair and physical strength: eggs, yogurt, fish, tofu, minced lean meat and nutritional supplements.

Add healthy calorie sources such as olive oil, butter, protein powder and oral nutritional supplements to prevent unintended weight loss caused by long-term fasting.

4. Avoid irritating food

Stay away from overly hot, spicy food, alcohol, strong tea and carbonated drinks. Avoid highly acidic food and food with bones or sharp edges to lower the risk of mucosal irritation and bleeding.

5. Drink water properly

Drink fluids between meals rather than drinking large amounts of soup or water while eating, which may cause early fullness and reduce overall food intake.

6. Use medical nutritional supplements in time

If you cannot eat enough and keep losing weight, take oral enteral nutrition powder or liquid under the guidance of doctors or clinical dietitians. For severe swallowing difficulty, follow medical advice for tube feeding instead of forcing oral intake.

7. Monitor body weight and symptoms

Weigh yourself regularly and inform your oncologist immediately if you keep losing weight. Seek medical help promptly and adjust your diet plan if you experience painful swallowing, obstruction or vomiting.

Question28: How should I gradually resume eating after esophageal cancer surgery?

Answer:

1. Early postoperative period (NPO stage)

You will be kept nil per os (no food or liquid) for a period after surgery. Nutrition will be provided via enteral feeding or intravenous infusion to reduce tension on the surgical anastomosis and lower the risk of anastomotic leakage. Follow your surgeon’s exact schedule.

2. Liquid diet phase

You may start thin, residue-free liquids once your doctor confirms proper healing of the anastomosis. Suitable options include rice water, strained vegetable broth, clear soup and commercial enteral nutrition formulas.

Eat small amounts frequently: 50–100 ml each time, with an interval of 2–3 hours. Keep food lukewarm; avoid overly hot drinks, carbonated beverages and rich creamy soups. Do not swallow quickly.

3. Semi-liquid diet phase

Transition 1–2 weeks after tolerating liquid food. Choose smooth, mushy foods: thin congee, steamed egg custard, soft tofu, fruit puree and well-cooked soft noodles. Keep frequent small meals and chew thoroughly. Avoid coarse, fibrous ingredients.

4. Soft food phase

After adapting to semi-liquid meals, slowly introduce soft foods such as soft rice, flaky fish, tender minced meat and well-steamed vegetables. Stay away from nuts, fried food, tough fibrous vegetables and bony, hard foods.

5. Transition to regular diet (long-term management)

Progress slowly; do not rush back to your preoperative diet. Maintain small, frequent meals (5–6 meals per day) long-term. Do not lie down immediately after eating; stay upright or walk gently for 30–60 minutes to prevent gastroesophageal reflux. Stop smoking and drinking alcohol; avoid spicy, extremely hot and irritating food.

Key general guidelines

- The speed of dietary progression varies individually. Always follow instructions from your surgeon and clinical dietitian.
- Stop eating and seek medical care promptly if you experience swallowing difficulty, chest pain, fever or vomiting.

Question29: How should the pain caused by esophageal cancer be managed?

Answer:

Pain management for esophageal cancer follows standardized comprehensive treatment principles, mainly including drug therapy, non-drug interventions, treatment targeting the tumor, and supportive care.

1. Pharmacological treatment (three-step analgesic ladder recommended by WHO)
   - Mild pain: Non-opioid analgesics such as paracetamol and nonsteroidal anti-inflammatory drugs.
   - Moderate pain: Weak opioids combined with non-opioid analgesics and adjuvant drugs.
   - Severe pain: Strong opioids as the core medicine, with auxiliary medications when necessary. Drugs should be taken regularly on schedule instead of only when pain occurs.
2. Anti-tumor treatment to relieve the source of pain

Radiotherapy, chemotherapy, targeted therapy or immunotherapy can shrink tumors, reduce compression, invasion and ulceration, and fundamentally ease cancer-related pain. Local radiotherapy is effective for pain from tumor invasion of surrounding tissues.

3. Non-pharmacological auxiliary measures

Psychological counseling, relaxation training, local palliative care, nutritional support, diet adjustment to avoid stimulating the esophageal mucosa. Interventional treatments such as nerve block can be adopted for refractory pain.

4. Prevention and management of complications

Esophageal inflammation, ulcer, stenosis and perforation will aggravate pain. Soft or liquid diet, acid suppression and mucosal protective agents are used to reduce esophageal irritation.

5. Individualized assessment

Continuously evaluate pain degree, adjust treatment plans timely, and pay attention to preventing and controlling side effects of analgesic drugs such as constipation, nausea and vomiting.

Question30: How often should I have follow-up examinations after treatment for esophageal cancer, and what items are included?

Answer:

Follow-up Frequency

1. Within 1–2 years after surgery or curative treatment: every 3 months
2. From the 3rd to 5th year: every 6 months
3. More than 5 years after treatment: once a year

*Note: For patients with advanced stage or high risk of recurrence, doctors may shorten the follow-up interval appropriately.*

Routine Follow-up Items

1. Medical inquiry and physical examination: assessment of swallowing function, body weight, pain, palpation of superficial lymph nodes.
2. Blood tests: routine blood test, liver and kidney function, tumor markers (SCC, CEA, etc.).
3. Imaging examination: contrast-enhanced CT of chest and abdomen (core examination).
4. Endoscopy: gastroscopy (esophagoscopy) to check anastomosis and local recurrence.
5. Optional examinations: cervical ultrasound, upper gastrointestinal radiography, whole-body PET-CT (applied when recurrence is highly suspected).
