# Supplementary material for: Comparative evaluation of large language models for esophageal cancer patient education: quality, reliability, readability, understandability, and actionability
Source: Front Med (Lausanne). 2026 Oct 14;13:1973075. doi: 10.3389/fmed.2026.1973075 (PMC13713412; doi:10.3389/fmed.2026.1973075)
Supplement: Supplementary file 2 [file Table2.docx]

**Supplementary Table2** The Global Quality Score (GQS) quality criteria.

| **Item features** | **Points** |
| --- | --- |
| Poor quality, poor flow, most information missing; not at all useful for patients | 1 |
| Generally poor quality and poor flow, some information listed, but many important topics missing; of very limited use to patients | 2 |
| Moderate quality; suboptimal flow, some important information is adequately discussed, but others poorly discussed, somewhat useful for patients | 3 |
| Good quality and generally good flow, most of the relevant information is listed, but some topics are not covered, useful for patients | 4 |
| Excellent quality and flow; very useful for patients | 5 |
