# Supplementary material for: Comparative evaluation of large language models for esophageal cancer patient education: quality, reliability, readability, understandability, and actionability
Source: Front Med (Lausanne). 2026 Oct 14;13:1973075. doi: 10.3389/fmed.2026.1973075 (PMC13713412; doi:10.3389/fmed.2026.1973075)
Supplement: Supplementary file 3 [file Table3.docx]

**Supplementary Table3** The Modified DISCERN (mDISCERN) quality criteria.

**Reliability Score**

1. Is the aim clear, concise, and understandable?

2. Are the sources of information reliable?

3. Is the information presented balanced and unbiased?

4. Are additional sources of information listed for patient reference?

5. Are areas of uncertainty mentioned?
