# Supplementary material for: Comparative evaluation of large language models for esophageal cancer patient education: quality, reliability, readability, understandability, and actionability
Source: Front Med (Lausanne). 2026 Oct 14;13:1973075. doi: 10.3389/fmed.2026.1973075 (PMC13713412; doi:10.3389/fmed.2026.1973075)
Supplement: Supplementary file 4 [file Table4.docx]

**Supplementary Table4 Domains and definitions of the Quality Analysis of Medical Artificial Intelligence(QAMAI) tool scoring system.**

| **Domain** | **Definition** |
| --- | --- |
| Accuracy | The information provided is accurate and up to date. |
| Clarity | The answer is clear and comprehensible in terms of language and scientific terminology. |
| Relevance | The information provided is relevant and directly answers the question posed. |
| Completeness | The response adequately covers all aspects of the question and provides sufficient information, including areas of uncertainty. |
| Provision of sources and references | The response provides reliable sources and references to support the health information presented. |
| Usefulness | The response meets the user's health information needs. |
