# Supplementary material for: Comparative evaluation of large language models for esophageal cancer patient education: quality, reliability, readability, understandability, and actionability
Source: Front Med (Lausanne). 2026 Oct 14;13:1973075. doi: 10.3389/fmed.2026.1973075 (PMC13713412; doi:10.3389/fmed.2026.1973075)
Supplement: Supplementary file 5 [file Table5.docx]

**Supplementary Table5 EQIP (Ensuring Quality Information for Patients) Checklist.**

| Item | EQIP item (short label) | Operational definition (score Yes if…) |
| --- | --- | --- |
| 1 | Source/origin stated | The response identifies a credible source type (e.g., guideline, society, hospital/clinic, peer-reviewed evidence) . |
| 2 | References provided | At least one verifiable reference cue is provided (e.g., guideline title/year, society guideline, PMID/DOI, named organization document, or a clearly identifiable citation). |
| 3 | Adequate background/context | Defines the topic and provides sufficient context (definitions, who it applies to, key concepts) before recommendations. |
| 4 | Accurate and unambiguous | Statements are clinically coherent and avoid internal contradictions; key recommendations are qualified where needed (e.g., ‘depends on severity/phenotype’) rather than presented as absolute when conditional. |
| 5 | Evidence-based/scientific | Recommendations are framed as evidence-based (e.g., aligns with guideline logic; avoids unproven claims; clearly distinguishes evidence-supported options from speculation). |
| 6 | Currency addressed | Includes an explicit ‘as of’ date/year, acknowledges that recommendations can change over time, or references a dated guideline/consensus (year stated). |
| 7 | Plain language | Uses patient-friendly wording and defines unavoidable medical terms in-line. |
| 8 | Helpful visuals/format aids | Uses an easy-to-follow table, stepwise algorithm, checklist, or other structured formatting that functions as a ‘visual aid’ in text (e.g., markdown table). |
| 9 | Key points easy to find | Highlights the most important content via headings, bullets, summary box, or an opening ‘key points’ section. |
| 10 | Clear structure | Logical organization (e.g., overview → key points → details → next steps), with coherent sectioning. |
| 11 | Practical usefulness | Provides actionable, applicable information for a patient (what to do/ask/monitor), not purely descriptive. |
| 12 | Bias/neutrality | Presents options fairly and avoids promotional tone, product/brand pushing, or one-sided framing. |
| 13 | Clear action guidance | Gives concrete guidance appropriate to the question (e.g., ‘when to seek urgent care’, ‘what to discuss with your doctor’, ‘typical monitoring’). |
| 14 | Privacy respected | Does not request identifiable personal information; if asking for context, uses non-identifying prompts (e.g., general symptoms) and includes a privacy caveat when relevant. |
| 15 | Minimizes jargon | Avoids unnecessary technical terms; when used, explains them in plain language. |
| 16 | Balanced perspective | Discusses benefits/risks/alternatives or uncertainty where relevant, rather than only benefits. |
| 17 | Engaging/presented well | Readable presentation that encourages continued reading (short paragraphs, bullets, headings; avoids wall-of-text). |
| 18 | Cultural/language needs considered | Acknowledges that recommendations/resources may differ by region and suggests seeking local guideline/clinician advice or provides culturally/regionally neutral phrasing. |
| 19 | Supports informed decisions | Explicitly supports decision-making (e.g., clarifies choices, tradeoffs, questions to ask, red flags, and decision criteria). |
| 20 | Clear conclusion/summary | Ends with a succinct conclusion or summary that reiterates key takeaways and next steps. |

According to the EQIP criteria, full compliance is scored as 1 point ("yes"), partial compliance as 0.5 points ("partly"), and non-compliance as 0 points ("no"). The final score is expressed as a percentage. The EQIP total score was calculated as ((Yes×1+Party×0.5+No×0)/20 - Does not apply)×100. A higher EQIP score indicates better quality of information presentation. The quality classification is as follows: 0%–25%, severe quality problems; 26%–50%, serious quality issues; 51%–75%, good quality with minor issues; 76%–100%, well written.
