# Supplementary material for: Comparative evaluation of large language models for esophageal cancer patient education: quality, reliability, readability, understandability, and actionability
Source: Front Med (Lausanne). 2026 Oct 14;13:1973075. doi: 10.3389/fmed.2026.1973075 (PMC13713412; doi:10.3389/fmed.2026.1973075)
Supplement: Supplementary file 6 [file Table6.docx]

**Supplementary Table6 Flesch Reading Ease Score(FRES)–Flesch-Kincaid Grade Level(FKGL) readability categories and grade-level estimates.**

| **FRES** | **Readability category** | **FKGL** | **Estimated grade** |
| --- | --- | --- | --- |
| 91–100 | Very easy | 5.0–5.9 | Fifth grade |
| 81–90 | Easy | 6.0–6.9 | Sixth grade |
| 71–80 | Fairly easy | 7.0–7.9 | Seventh grade |
| 61–70 | Standard | 8.0–9.9 | Eighth-Ninth grade |
| 51–60 | Fairly difficult | 10–12.9 | Tenth-Twelfth grade |
| 31–50 | Difficult | 13.0–16.9 | College |
| 0–30 | Very difficult | >17.0 | University graduate |
