# Supplementary material for: Comparative evaluation of large language models for esophageal cancer patient education: quality, reliability, readability, understandability, and actionability
Source: Front Med (Lausanne). 2026 Oct 14;13:1973075. doi: 10.3389/fmed.2026.1973075 (PMC13713412; doi:10.3389/fmed.2026.1973075)
Supplement: Supplementary file 7 [file Table7.docx]

**Supplementary Table7 Formula for calculating the readability score of an online calculator.**

| **Index** | **Full name** | **Formula** |
| --- | --- | --- |
| ARI | Automated Readability Index | ARI = 4.71×(characters/words)+0.5×(words/sentences)-21.43 |
| CLI | Coleman-Liau Index | CLI = 0.0588L-0.296S-15.8 |
| FKGL | Flesch-Kincaid grade level | FKGL = 0.39×ASL+11.8×ASW-15.59 |
| GFI | Gunning Fog Index | GFI = 0.4×[(words/sentences)+100×(complex words/words)] |
| SMOG | Simple measure of gobbledygook | SMOG = 1.0430×√[30×(polysyllables/sentences)]+3.1291 |
| FRES | Flesch Reading Ease Score | FRES = 206.835-1.015×ASL-84.6×ASW |

Characters, number of letters/numbers; Words, number of words; Sentences, number of sentences; L, average number of letters per 100 words; S, average number of sentences per 100 words; ASL, average sentence length (words / sentences); ASW, average syllables per word (syllables/words); Complex words, words with three or more syllables; Polysyllables, words with three or more syllables; ASL, average sentence length; ASW, average syllables per word.
